# Global Epidemiological Transition of Atrial Fibrillation/Flutter (1990–2021): Multidimensional Burden Dynamics and Socioeconomic Health Gradients Across 204 Countries and Territories

**DOI:** 10.31083/RCM45091

**Published:** 2025-12-18

**Authors:** Jingmei Sun, Yingying Lu, Yifan Bao, Dechun Yin, Xiufen Qu

**Affiliations:** ^1^Department of Cardiology, the First Affiliated Hospital, Harbin Medical University, 150001 Harbin, Heilongjiang, China

**Keywords:** atrial fibrillation, atrial flutter, prevalence, incidence, disability-adjusted life years

## Abstract

**Background:**

This study aimed to decode the spatiotemporal trajectory of atrial fibrillation/flutter (AF/AFL) burden (1990–2021) through hierarchical quantification of socioeconomic health gradients and Bayesian projection modeling across 204 countries and territories until 2036.

**Methods:**

This study, based on data from the 2021 Global Burden of Disease (GBD) study, systematically investigates the geopolitical and temporal dynamics of AF/AFL from 1990 to 2021. This study quantified the impact of population structure, age distribution, and disease rates on the disease burden, assessed the inequality of burden among different countries, and predicted the disease trends for the next 15 years.

**Results:**

From 1990 to 2021, when the age-standardized death rate (ASDR) was the only indicator showing an upward trend (estimated annual percentage change (EAPC) = 0.1 (0.06–0.13)), the absolute number of AF/AFL cases continued to rise. Decomposition analysis revealed that population growth (43.17%) and aging (56.31%) were the primary drivers of the global AF/AFL burden in 2021. The study found that from 1990 to 2021, inequality in the social indicators index (SDI) worsened, whereas the slope index of inequality (SII) values for AF/AFL incidence (41.68 vs. 81.71), prevalence (499.54 vs. 1076.65), mortality (3.23 vs. 8.50), and disability-adjusted life years (DALYs) (82.36 vs. 189.81) all increased. Notably, the global AF/AFL burden is projected to continue rising through 2036. The age-standardized incidence rate (ASIR) (52.36 vs. 56.07) for AF/AFL is expected to increase annually, while the ASDR (4.12 vs. 3.93) and age-standardized DALYs rate (ASDAR) (107.45 vs. 90.87) are projected to decline. However, the number of cases is expected to maintain growth.

**Conclusions:**

This study shows that the global burden of AF/AFL has an overall increasing trend from 1990 to 2021, primarily driven by population growth and aging. Countries with a high SDI bear a disproportionately high burden of AF/AFL, while SDI-related inequalities among countries have worsened over time. This study highlights the significant challenges in the prevention and management of AF/AFL, including the rising number of cases and the unequal distribution of the subsequent burden worldwide. These findings may be instructive for developing more effective public health policies and reasonably allocating medical resources.

## 1. Introduction

Atrial fibrillation (AF)/atrial flutter (AFL) is the most common type of sustained arrhythmia, leading to significant morbidity and mortality. Driven by demographic changes and the iteration of non-invasive electrocardiographic monitoring technologies, the public health priority of AF/AFL continues to rise. Evidence-based medical research indicates that AF/AFL elevated risk of all-cause mortality (the hazard ratio (HR) 2.17; 95% Confidence Interval (CI), 1.89–2.49) and substantially amplifies composite cardiovascular endpoint events (HR = 5.43 for stroke; HR = 3.82 for heart failure hospitalization) [1].

From a global distribution perspective, there is a significant geographical imbalance in the management of AF/AFL. Low-income countries, constrained by shortages in medical resources and inadequate prevention and control systems, have low patient awareness and insufficient standardized treatment rates, resulting in higher risks for complications [2]. In contrast, high-income countries, despite having more mature diagnostic and treatment networks, face continuously rising incidence rates due to rapid aging, leading to an annual increase in related medical expenditures [3]. With changes in population structure and epidemiological patterns, the disease spectrum of AF/AFL may undergo dynamic adjustments. Existing research, although partially revealing the characteristics of its disease burden, often focuses on specific indicators or regional analyses [4, 5], and lacks a systematic perspective.

Leveraging the Global Burden of Disease (GBD) 2021 database, this study presents a comprehensive, multi-dimensional analysis of the disease burden of AF/AFL, its spatiotemporal evolution, and associated health equities. The research framework encompasses five core modules: (1) constructing a global-regional-national three-tier epidemiological map; (2) conducting comprehensive analyses of overall trends, local characteristics, and multi-dimensional associations; (3) decomposing studies combining demographic and epidemiological indicators; (4) assessing transnational health inequalities using World Health Organization (WHO) standard methods; (5) establishing predictive models to forecast disease burden trends up to 2036. This systematic approach aims to provide comprehensive evidence-based insights for optimizing global AF prevention and control strategies.

## 2. Methods

### 2.1 Data Sources

This study is based on the standardized methodology of the GBD 2021 collaborative network, integrating multi-source data to establish a global health database. Through the Global Health Data Exchange platform (GHDx), indicators such as incidence, prevalence, mortality, and disability-adjusted life years (DALYs) for AF/AFL were obtained. Bayesian meta-regression algorithms were used for age, sex, and regional stratification estimates, providing 95% uncertainty intervals (UI). All rate values were standardized to per 100,000 population to ensure transnational and intertemporal comparability. The study introduced the Social Indicators Index (SDI) as a covariate, dividing national development levels into five tiers to analyze social determinants of disease burden. In data preprocessing, diagnostic technology differences were specifically corrected to reduce detection rate bias in high SDI regions due to advanced technology.

Data Source: All study data are publicly accessible via the Global Health Data Exchange: http://ghdx.healthdata.org/gbd-results-tool. The Institutional Review Board (IRB) of The First Affiliated Hospital, Harbin Medical University waived formal ethics approval, as this study utilized publicly available, de-identified datasets.

### 2.2 Trend Analysis

#### 2.2.1 Overall Trend Modeling 

A log-linear regression model was constructed based on Age-standardized rate (ASR): In(ASR) = α + βX + e. X stands for the calendar year under consideration. The y-intercept is denoted by α, while β indicates the slope, which represents the trend over time. Any error in the model is accounted for by e. The estimated annual percentage change (EAPC) and its 95% confidence interval were calculated through the regression coefficient β(EAPC = [exp(β) – 1] × 100%) [6].

#### 2.2.2 Segmented Trend Identification

The Joinpoint regression model was used to identify inflection points in the ASR time series, dividing the overall trend into multiple linear subsegments. The annual percentage change (APC) was calculated for each subsegment, and the average annual percentage change (AAPC) for 1990–1999, 2000–2009, 2010–2021, and the entire period (1990–2021) was derived by weighting the regression coefficients of each subsegment. Monte Carlo simulation (4499 permutation tests) was used to calculate the AAPC confidence intervals, with Bonferroni correction to control for multiple testing errors [7, 8].

#### 2.2.3 Time Effect Deconstruction

The age-period-cohort (APC) model’s intrinsic estimator method was used to separate the three effects: Model structure: ln(Yi,j,k) = µ + αi (age effect) + βj (period effect) + γk (cohort effect) + εi,j,k, where Yi,j,k represents the incidence, prevalence, and mortality for the i-th age group (5-year intervals: 30–34 to 95+ years), the j-th period group (1992–1996 to 2017–2021), and the k-th birth cohort group (1897–1901 to 1987–1991) [9, 10]; εi,j,k denoted the residual of the model. Effect quantification: estimated coefficients were converted to exponential relative risks (RR), reflecting the degree of risk deviation of specific age-period-cohorts from the overall mean. This method eliminates model collinearity through principal component regression, achieving unbiased estimates of the three effects. This analytical system captures both global long-term trends and local fluctuations, while revealing the interactive impact of population aging, era changes, and generational differences on disease burden.

### 2.3 Decomposition Analysis

This study employed the Das Gupta factor decomposition method to analyze the drivers of the AF/AFL disease burden from 1990 to 2021, attributing changes in DALYs to the effects of population an aging, population growth, and epidemiological evolution through the establishment of a three-variable interaction model. The DALYs calculation model was constructed based on the following theoretical framework:

${\mathrm{DALYs}}_{\gamma }=\sum_{i=1}^{20}\left(a_{i,\gamma }\times p_{\gamma }\times e_{i,\gamma }\right)$

In the equation, the core parameters include the age structure for year γ (aγ = {ai,γ}), the total population (pγ), and the age-specific DALYs incidence rates (eγ = {ei,γ}). By using the control variable method to systematically isolate confounding factors, the independent contribution of each driving factor responsible for the increase in DALYs was precisely quantified [11].

### 2.4 Inequality Analysis

This study employs a dual-dimensional quantitative framework to analyze the socioeconomic differences in the burden of AF/AFL. First, countries are ranked based on the SDI, and a robust weighted least squares regression model is used to calculate the Slope Index of Inequality (SII), representing the absolute difference in disease burden. Second, the Concentration Index (CI) is calculated by plotting the health burden-population distribution curve, indicating the relative distribution of the disease burden. This method considers both the absolute magnitude and relative distribution of health inequalities, utilizing non-parametric sorting and robust estimation techniques to enhance the statistical significance and comparability across time periods [12, 13].

### 2.5 Predictive Analysis

Based on WHO population statistics, construct a burden model for AF/AFL was constructed to calculate the ASR of incidence, mortality, and DALYs for AF/AFL. We employed Bayesian age-period-cohort (BAPC) models to project trends in the burden of AF/AFL disease up to 2036. These models utilize the integrated nested Laplace approximations (INLAs) method to derive marginal posterior distributions, thereby effectively overcoming the challenges of mixing and convergence commonly encountered with traditional Bayesian methods, such as Markov chain Monte Carlo sampling [14]. The analysis was conducted using the BAPC and INLAs packages in R version 4.3.2 (R Foundation for Statistical Computing, Vienna, Austria).

## 3. Results

### 3.1 AF/AFL Burden at Global, Region, and Nations

**Fig. 1.**
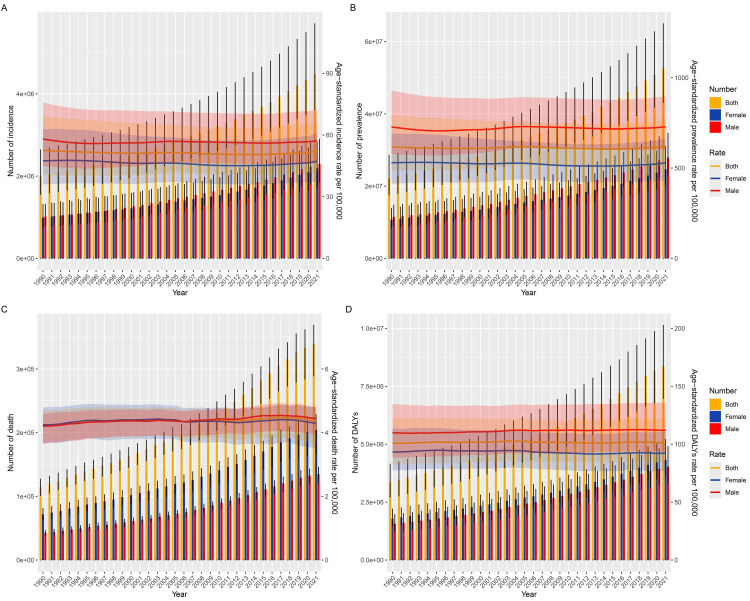
**Age-standardized rates and numbers of incidence, prevalence, deaths and DALYs in patients with AF/AFL from 1990 to 2021**. (A) Incidence. (B) Prevalence. (C) Deaths. (D) DALYs. ASR, age-standardized rate; DALYs, disability-adjusted life years; AF/AFL, Atrial fibrillation/atrial flutter.

Globally, the incident cases of AF/AFL nearly doubled from 2,006,571 (95% UI: 1,554,971–2,640,379) in 1990 to 4,484,926 (95% UI: 3,610,620–5,706,019) in 2021. Besides, the age-standardized incidence rate (ASIR) remained stable at 52.51(95% UI: 40.39–69.01) in 1990 versus 52.12 (95% UI: 41.85–66.23) in 2021 per 100,000 population, with the EAPC being –0.07 (95% CI: –0.01–0.04) (Table 1 and Fig. 1A).

**Table 1.** **Incidence of AF/AFL between 1990 and 2021 at the global and regional level**.

Location		Incidence in 1990		Incidence in 2021		1990–2021 EAPC
		Number (95% UI)	Rate (95% UI)	Number (95% UI)	Rate (95% UI)	EAPC_95% CI
Global		2,006,571 (1,554,971, 2,640,379)	52.51 (40.39, 69.01)	4,484,926 (3,610,620, 5,706,019)	52.12 (41.85, 66.23)	–0.07 (–0.10, 0.04)
Sex						
	Males	1,015,287 (786,876, 1,319,475)	58.00 (44.89, 75.8)	2,295,811 (1,853,679, 2,899,436)	57.11 (46.19, 72.14)	–0.01 (–0.04, 0.02)
	Females	991,284 (754,261, 1,318,501)	47.43 (35.96, 62.83)	2,189,116 (1,723,110, 2,821,648)	47.26 (37.38, 60.87)	–0.14 (–0.19, 0.09)
SDI region						
	High SDI	722,978 (553,815, 943,982)	64.54 (50.18, 83.44)	1,334,730 (1,139,377, 1,572,638)	65.10 (56.11, 76.05)	–0.07 (–0.13, 0.02)
	High-middle SDI	476,340 (365,294, 628,545)	48.86 (37.88, 63.69)	934,174 (740,417, 1,198,977)	47.16 (37.67, 60.25)	–0.17 (–0.22, 0.13)
	Middle SDI	454,020 (351,991, 596,801)	48.88 (37.12, 64.96)	1,330,376 (1,031,639, 1,752,211)	51.11 (39.20, 67.85)	0.13 (0.11, 0.16)
	Low-middle SDI	268,077 (206,734, 354,728)	49.52 (37.34, 65.82)	682,885 (523,053, 912,036)	50.99 (38.44, 67.86)	0.10 (0.09, 0.10)
	Low SDI	82,742 (63,353, 109,675)	41.74 (31.59, 55.83)	198,494 (153,240, 261,939)	43.25 (32.70, 57.75)	0.12 (0.11, 0.14)
GBD region						
	Andean Latin America	10,658 (8333, 13,962)	54.40 (41.63, 72.43)	33,004 (25,562, 43,750)	56.44 (43.34, 75.08)	0.20 (0.16, 0.24)
	Australasia	17,291 (15,419, 19,399)	73.00 (65.35, 81.38)	38,348 (29,260, 50,108)	73.34 (57.30, 94.35)	0.07 (0.02, 0.12)
	Caribbean	15,317 (11,699, 20,439)	60.10 (46.18, 79.67)	31,921 (24,658, 42,125)	59.14 (45.45, 78.13)	–0.05 (–0.06, 0.04)
	Central Asia	20,049 (15,184, 26,387)	44.05 (33.40, 58.03)	36,231 (27,486, 47,288)	44.53 (33.54, 57.85)	0.02 (0.01, 0.03)
	Central Europe	76,656 (57,337, 101,380)	50.47 (38.22, 65.71)	122,626 (94,472, 154,039)	55.16 (44.12, 68.47)	0.09 (0.03, 0.16)
	Central Latin America	49,656 (38,646, 65,616)	62.71 (48.02, 83.50)	154,316 (119,073, 205,022)	62.38 (47.92, 83.05)	0.01 (0.00, 0.03)
	Central Sub-Saharan Africa	7721 (5859, 10,151)	40.17 (30.36, 53.20)	19,101 (14,928, 25,118)	39.56 (29.91, 52.82)	–0.07 (–0.08, 0.06)
	East Asia	321,766 (246,730, 424,271)	42.98 (32.71, 56.93)	953,898 (737,314, 1,249,815)	45.11 (35.12, 59.61)	0.15 (0.06, 0.23)
	Eastern Europe	130,200 (98,553, 170,809)	46.52 (35.77, 60.37)	176,794 (133,418, 231,758)	50.47 (38.83, 65.64)	0.30 (0.24, 0.37)
	Eastern Sub-Saharan Africa	24,732 (19,155, 32,329)	36.74 (28.10, 49.06)	62,823 (49,401, 81,796)	39.66 (30.44, 52.91)	0.23 (0.20, 0.27)
	High_income Asia Pacific	86,518 (66,803, 113,257)	43.22 (33.54, 56.82)	152,471 (116,375, 199,323)	36.27 (29.16, 46.41)	–0.57 (–0.81, 0.33)
	High_income North America	278,728 (205,978, 367,024)	77.60 (59.70, 101.77)	586,320 (534,188, 649,281)	88.24 (80.92, 97.25)	0.41 (0.36, 0.46)
	North Africa And Middle East	49,894 (38,440, 65,909)	33.93 (25.04, 45.30)	145,164 (114,585, 186,086)	35.04 (26.72, 45.54)	0.03 (0.01, 0.05)
	Oceania	1295 (1006, 1696)	51.68 (39.37, 68.62)	3484 (2741, 4545)	52.33 (39.94, 69.52)	0.04 (0.02, 0.06)
	South Asia	249,150 (190,686, 331,288)	50.08 (37.35, 66.50)	698,920 (527,866, 934,818)	51.01 (38.09, 67.89)	0.08 (0.07, 0.08)
	Southeast Asia	135,279 (105,015, 176,227)	58.77 (44.77, 78.09)	369,518 (285,921, 483,602)	59.66 (45.60, 79.28)	0.07 (0.06, 0.08)
	Southern Latin America	19,573 (14,786, 26,223)	42.83 (32.53, 57.25)	28,852 (23,283, 35,802)	33.00 (26.86, 40.72)	–0.93 (–1.14, 0.73)
	Southern Sub-Saharan Africa	11,862 (9126, 15,604)	47.32 (35.92, 63.20)	25,556 (19,614, 33,834)	47.60 (36.27, 63.27)	0.00 (–0.01, 0.01)
	Tropical Latin America	60,437 (46,588, 78,717)	68.71 (52.98, 90.29)	172,830 (133,612, 224,369)	67.55 (52.09, 88.35)	–0.24 (–0.29, 0.19)
	Werstern Europe	410,476 (310,302, 539,191)	69.97 (54.22, 90.22)	600,735 (491,661, 740,671)	68.19 (56.60, 82.27)	–0.25 (–0.31, 0.18)
	Werstern Sub-Saharan Africa	29,311 (22,484, 38,784)	36.54 (27.78, 49.07)	72,014 (56,373, 94,024)	39.82 (30.36, 53.28)	0.31 (0.29, 0.34)

AF/AFL, Atrial fibrillation/atrial flutter; CI, confidence interval; EAPC, estimated annual percentage change; UI, uncertainty interval; SDI, socio-demographic index; GBD, Global Burden of Disease.

Meanwhile, the global prevalent cases of AF/AFL surged from 22,214,495 (95% UI: 17,526,215–28,522,555) in 1990 to 52,552,045 (95% UI: 43,137,876–64,963,854) in 2021. The age-standardized prevalence rate (ASPR) showed a minimal variation of EAPC = –0.01 (95% CI: –0.04–0.01) from 616.58 (95% UI: 485.22–795.26) in 1990 compared to 620.51 (95% UI: 511.36–768.88) in 2021 per 100,000 population (**Supplementary Table 1** and Fig. 1B).

AF/AFL-attributable deaths tripled from 114,540 (95% UI: 101,326–127,155) in 1990 to 338,947 (95% UI: 288,954–368,613) in 2021 globally. The age-standardized death rate (ASDR) exhibited a slight increased from 4.24 (95% UI: 3.69–4.71) in 1990 to 4.36 (95% UI: 3.69–4.75) in 2021 per 100,000 population with the EAPC being 0.1 (95% CI: 0.06–0.13) (**Supplementary Table 2** and Fig. 1C).

The global DALYs cases of AF/AFL increased 2.5-fold from 3,358,708 (95% UI: 2,715,430–4,141,735) in 1990 to 8,358,894 (95% UI: 6,970,688–10,133,489) in 2021. The age-standardized DALYs rate (ASDAR) remained constant at 100.81 (95% UI: 82.82–122.62) in 1990 versus 101.4 (95% UI: 84.89–122.41) in 2021 per 100,000 population (**Supplementary Table 3** and Fig. 1D).

### 3.2 AF/AFL Burden in Region

In terms of regional distribution, East Asia dominated in incident cases [953,898 (95% UI: 737,314–1,249,815)], prevalent cases [11,215,156 (95% UI: 8,885,909–14,572,495)], and DALYs cases [1,723,468 (95% UI: 1,364,758–2,143,575)], while Western Europe recorded highest number of deaths [72,184 (95% UI: 58,846–79,292)] in 2021. Oceania exhibited minimal cases across all metrics: [3484 (95% UI: 2741–4545)], prevalent cases [34,994 (95% UI: 27,519–45,179)], deaths [185 (95% UI: 132–238)], and DALYs cases [6606 (95% UI: 5044–8584)] in 2021. High-income North America was the region with the highest ASIR [88.24 (95% UI: 80.92–97.25)] and ASPR [1031.17 (95% UI: 952.3–1117.88)], and Australasia topped the ASDR [6.58 (95% UI: 5.39–7.25)] and ASDAR [147.83 (95% UI: 121.51–179.59)] in 2021. Among regions in 2021, Southern Latin America had the lowest ASIR [33 (95% UI: 26.86–40.72)], North Africa and Middle East had the lowest ASPR [366.92 (95% UI: 291.62–468.4)], Central Asia had the lowest ASDR [2.37 (95% UI: 2.09–2.6)], and High-income Asia Pacific had the lowest ASDAR [69.03 (56.4–84.74)]. In terms of growth rate, High-income North America saw the fastest increase in ASIR [0.41 (95% UI: 0.36–0.46)], followed by East Asia in ASPR [0.46 (95% UI: 0.36–0.55)]. South Asia experienced the most rapid growth in ASDR [1.58 (95% UI: 1.38–1.78)] and ASDAR [0.74 (95% UI: 0.66–0.82)] (Table 1 and **Supplementary Tables 1–3**).

### 3.3 AF/AFL Burden in Nations

**Fig. 2.**
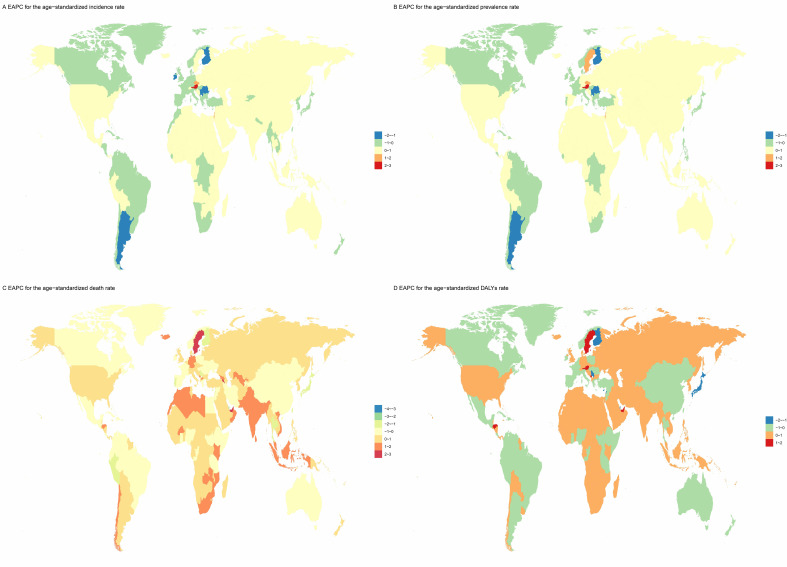
**Geographic heat map**. (A) EAPC of AF/AFL patient incidence from 1990 to 2021. (B) EAPC of AF/AFL patient prevalence from 1990 to 2021. (C) EAPC of AF/AFL patient death from 1990 to 2021. (D) EAPC of AF/AFL patient DALYs from 1990 to 2021. EAPC, estimated annual percentage change.

**Fig. 3.**
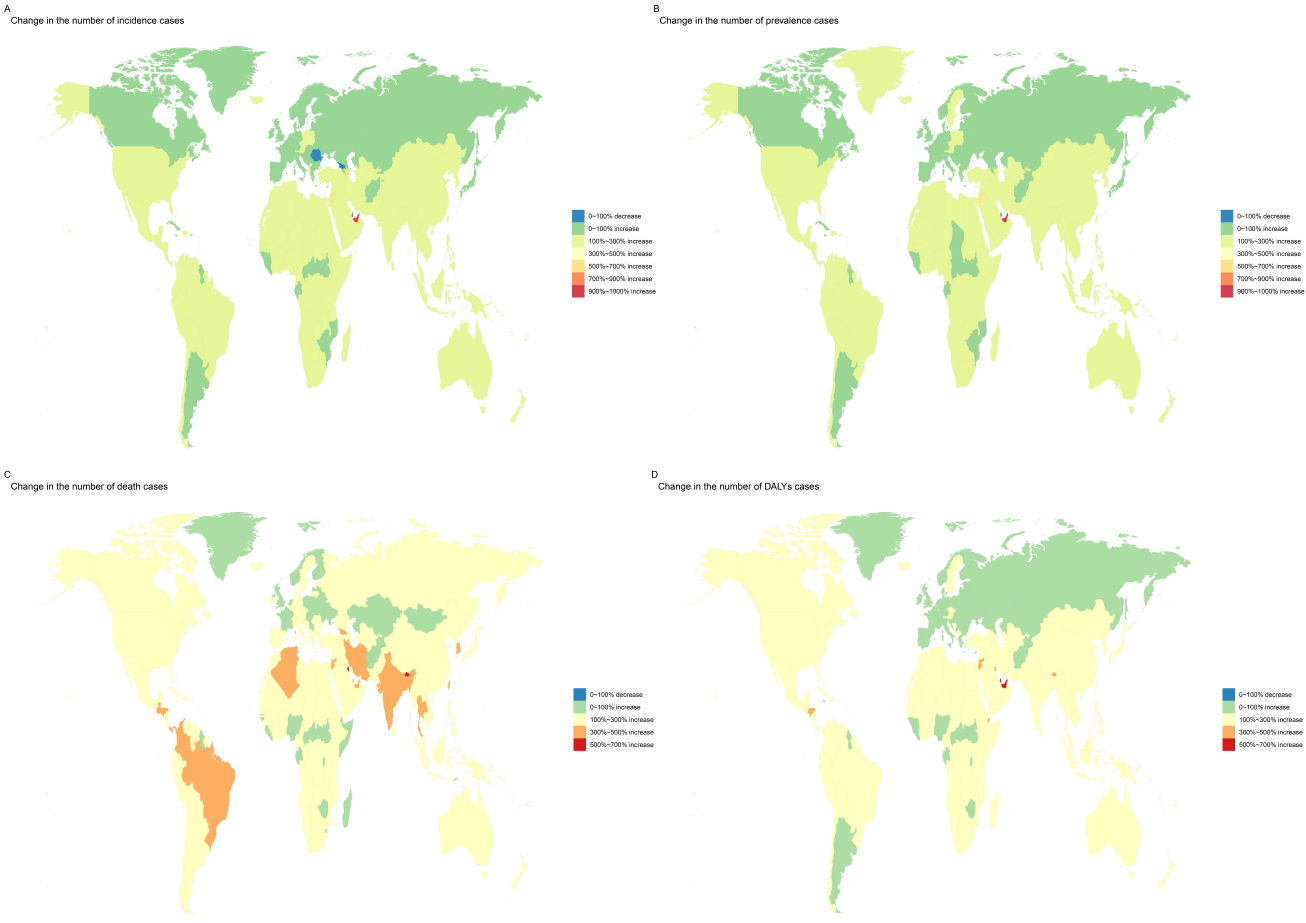
**Geographic heat map**. (A) Change in numbers of AF/AFL patient incidence from 1990 to 2021. (B) Change in numbers of AF/AFL patient prevalence from 1990 to 2021. (C) Change in numbers of AF/AFL patient death from 1990 to 2021. (D) Change in numbers of AF/AFL patient DALYs from 1990 to 2021.

In 2021, China had the highest number of incident cases [916,180 (95% UI: 707,384–1,201,381)], the highest number of prevalent cases [10,775,721 (95% UI: 8,531,627–14,014,036)], the highest number of death cases [64,728 (95% UI: 51,765–77,729)], and the highest number of DALYs cases [1,653,117 (95% UI: 1,303,681–2,056,459)] (**Supplementary Tables 4–7**; **Supplementary Fig. 1**). In 2021, the highest ASIR [123.84 (95% UI: 92.51–159.67)] and ASPR [1529.82 (95% UI: 1166.57–1943.26)] of AF/AFL was observed in Sweden, and the highest ASDR [17.26 (95% UI: 13.44–21.44)] and ASDAR [266.09 (95% UI: 221.18–320.05)] were found in Montenegro. In contrast, the lowest ASIR [27.35 (95% UI: 24.06–31.31)], and ASPR [282.89 (95% UI: 252.21–314.68)] were found in Turkey, the lowest ASDR [1.15 (95% UI: 0.92–1.44)] were found in Tajikistan, the lowest ASDAR [50.52 (95% UI: 38.39–65.59)] were found in Singapore (**Supplementary Tables 4–7**; **Supplementary Fig. 2**). Austria had the highest EAPC for ASIR [2.21 (95% CI: 2.07–2.34)] and ASPR [2.33 (95% CI: 2.19–2.48)], while the Uganda led in ASDR [2.97 (95% CI: 1.9–4.04)] and ASDAR [1.96 (95% CI: 1.34–2.58)] EAPC. Argentina had the lowest EAPC for ASIR [95% CI: –1.31 (–1.62 – –0.99)], Gambia for ASPR [95% CI: –1.17 (–1.44 – –0.9)], Guinea-Bissau for ASDR [95% CI: –3 (–3.56 – –2.45)], and Korea for ASDAR [95% CI: –1.71 (–1.91 – –1.5)] (**Supplementary Tables 4–7**; Fig. 2). The United Arab Emirates led in the multiplication of cases for incidence (971%), prevalence (942%), and DALYs (660%), while Kuwait had the highest multiplication in the number of death cases (606%) (**Supplementary Tables 4–7**; Fig. 3).

### 3.4 AF/AFL Burden in SDI Regions

**Fig. 4.**
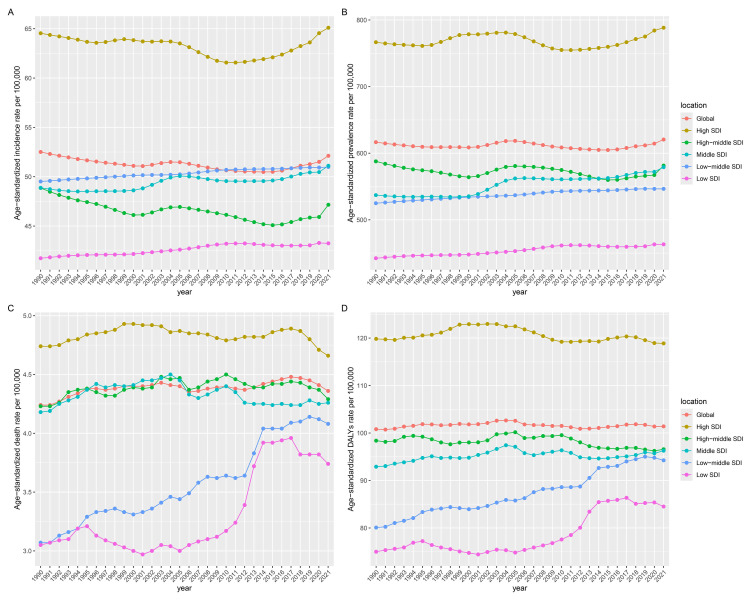
**Temporal trends of the burden of AF/AFL from 1990 to 2021 by SDI quintiles**. (A) Incidence. (B) Prevalence. (C) Deaths. (D) DALYs. SDI, sociodemographic index.

Among the five SDI regions in 2021, high-SDI regions demonstrated the highest ASIR [65.1 (95% UI: 56.11–76.05)], ASPR [788.35 (95% UI: 690.97–910.9)], ASDR [4.66 (95% UI: 3.88–5.08)], and ASDAR [118.88 (95% UI: 99.51–141.23)], and low-SDI regions consistently showed the lowest ASIR [43.25 (95% UI: 32.7–57.75)], ASPR [463.23 (95% UI: 362.02–602.71)], ASDR [3.74 (95% UI: 2.57–4.9)], and ASDAR [84.52 (95% UI: 63.65–108.24)]. The former maintained stable ASRs for all indicators, while the latter exhibited an increase across all metrics (Table 1 and **Supplementary Tables 1–3**; Fig. 4).

### 3.5 Local Trends in AF/AFL Burden

**Fig. 5.**
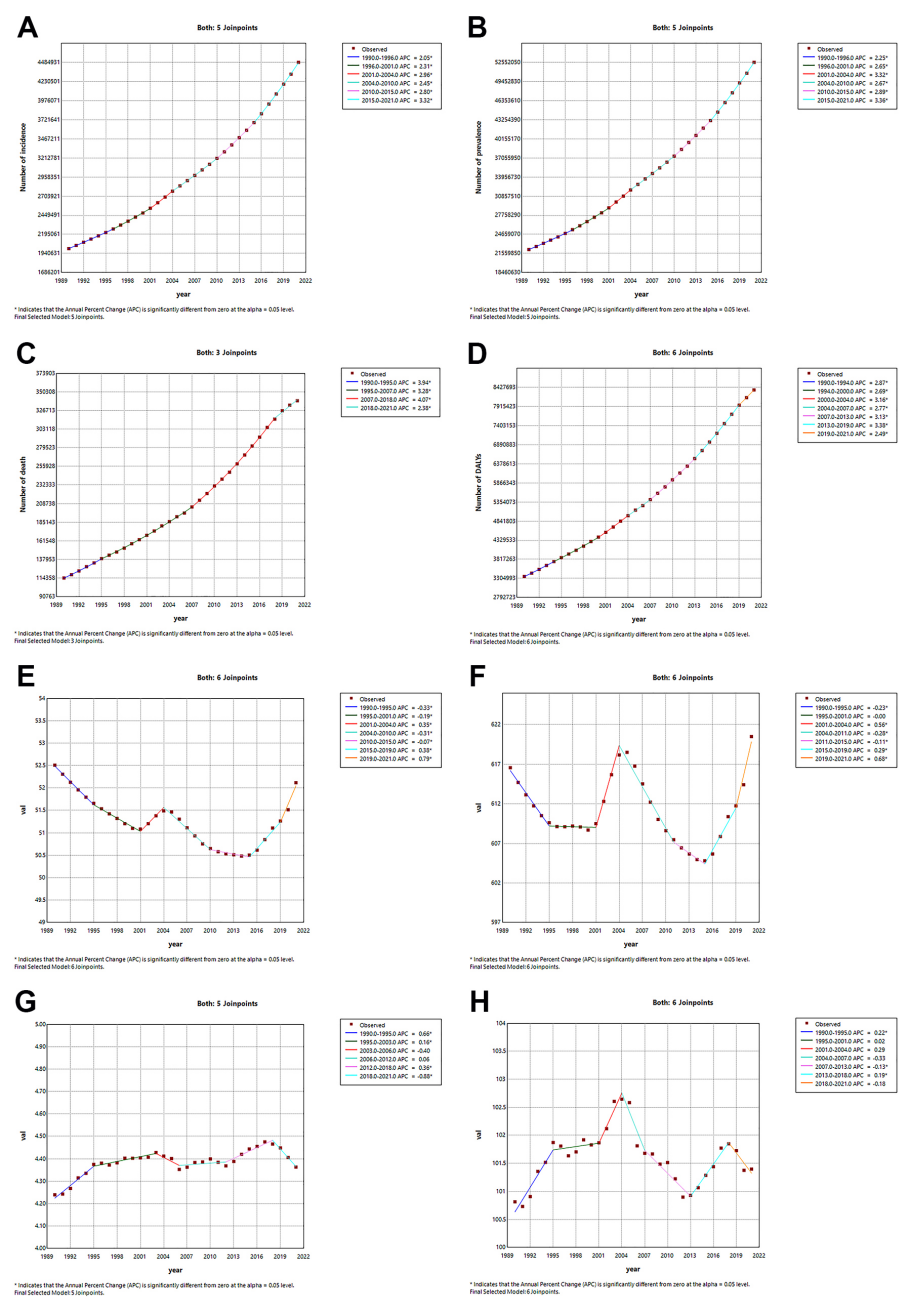
**Temporal trends of case numbers and ASRs for AF/AFL from 1990 to 2021: A Joinpoint regression analysis**. (A) Number of incidence. (B) Age-standardized incidence rate. (C) Number of prevalence. (D) Age-standardized prevalence rate. (E) Number of death. (F) Age-standardized death rate. (G) Number of DALYs. (H) Age-standardized DALYs rate. APC, annual percentage change. * indicates that the annual percent change (APC) is significantly different from zero at the alpha = 0.05 level.

Between 1990 and 2021, AF/AFL demonstrated sustained growth across all burden metrics: incidence [AAPC = 2.624 (95% CI: 2.599–2.648)], prevalence [AAPC = 2.817 (95% CI: 2.798–2.837)], mortality [AAPC = 3.578 (95% CI: 3.519–3.638)], and DALYs [AAPC = 2.987 (95% CI: 2.934–3.04)] attributed to AF/AFL (**Supplementary Table 8**). Peak increase for incidence [APC = 3.324 (3.294–3.356)] (Fig. 5A) and prevalence [APC = 3.363 (95% CI: 3.337–3.389)] (Fig. 5B) was observed from 2015 to 2021, while the mortality cases [APC = 4.070 (95% CI: 4.006–4.133)] (Fig. 5C) grew most rapidly from 2007 to 2018, and the DALYs [3.381 (95% CI: 3.3–3.462)] (Fig. 5D) saw their fastest growth from 2013 to 2019. The ASIR generally showed a decreasing trend [AAPC = –0.027 (95% CI: –0.034 – –0.02)], but there were periods of local increasing trends, specifically rising between 2001 and 2004 [APC = 0.348 (95% CI: 0.297–0.4)], 2015 and 2019 [APC = 0.384 (95% CI: 0.359–0.409)], and 2019 and 2021 [APC = 0.787 (95% CI: 0.735–0.838)], while decreasing between 1990 and 1995 [APC = –0.331 (95% CI: –0.344 – –0.318)], 1995 and 2001 [APC = –0.192 (95% CI: –0.205 – –0.18)], and 2004 and 2010 [APC = –0.307 (95% CI: –0.318 – –0.296)](**Supplementary Table 9**; Fig. 5E). The ASPR generally showed an increasing trend [AAPC = 0.019 (95% CI: 0.004–0.034)], decreasing between 1990 and 1995 [APC = –0.229 (95% CI: –0.256 – –0.201)], 2004 and 2011 [APC = –0.285 (95% CI: –0.301 – –0.268)], and 2011 and 2015 [APC = –0.113 (95% CI: –0.163 – –0.064)], and increasing between 2001 and 2004 [APC = 0.564 (95% CI: 0.46–0.668)], 2015 and 2019 [APC = 0.291 (95% CI: 0.241–0.342)], and 2019 and 2021 [APC = 0.682 (95% CI: 0.578–0.787)] (**Supplementary Table 9**; Fig. 5F). The ASDR generally showed an increasing trend [AAPC = 0.106 (95% CI: 0.031–0.181)], decreasing between 2018 and 2021 [APC = –0.878 (95% CI: –1.218 – –0.536)], and increasing between 1990 and 1995 [APC = 0.665 (95% CI: 0.517–0.813)], 1995 and 2003 [APC = 0.162 (95% CI: 0.083–0.242)], and 2012 and 2018 [APC = 0.365 (95% CI: 0.225–0.506)] (**Supplementary Table 9**; Fig. 5G). The ASDAR generally remained stable [AAPC = 0.022 (95% CI: –0.034–0.078)], increasing between 1990 and 1995 [APC = 0.219 (95% CI: 0.13–0.307)] and 2013 and 2018 [APC = 0.189 (95% CI: 0.074–0.304)], and decreasing between 2007 and 2013 [APC = –0.135 (95% CI: –0.216 – –0.054)] (**Supplementary Table 9**; Fig. 5H). When examining 10-year intervals, the incidence [APC = 3.086 (95% CI: 3.057–3.116)] and prevalence [APC = 3.149 (95% CI: 3.125–3.173)] number increased most rapidly between 2010 and 2021, the number of mortality [APC = 3.646 (95% CI: 3.542–3.75)] grew more rapidly between 1990 and 1999 and DALYs [APC = 3.15 (95% CI: 3.075–3.224)] between 2010 and 2021 (**Supplementary Table 8**). Similarly, the ASIR [APC = 0.253 (95% CI: 0.239–0.266)] and ASPR [APC = 0.162 (95% CI: 0.134–0.191)] grew the fastest between 2010 and 2021, and the ASR for mortality [APC = 0.441 (95% CI: 0.359–0.523)] and DALYs [APC = 0.130 (95% CI: 0.075–0.186)] rose faster between 1990 and 1999 (**Supplementary Table 9**).

### 3.6 Age-period-cohort Analysis on AF/AFL

**Fig. 6.**
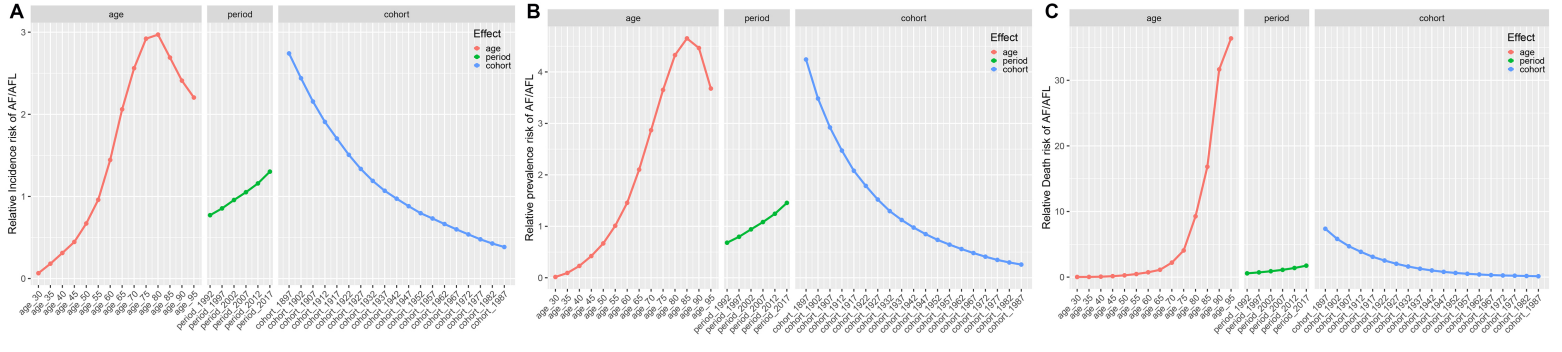
**The effects of age, period, and birth cohort on the relative risk of AF/AFL incidence, prevalence, and death**. (A) Incidence; (B) Prevalence; (C) Death.

The age-period-cohort (APC) modeling framework revealed distinct epidemiological trajectories (Fig. 6). Furthermore, the period effect analysis shows that after adjusting for period and cohort confounding factors, the relative risks of both incidence and prevalence exhibit an inverted U-shaped distribution, with peaks at 80–84 years (RR = 2.97) and 85–89 years (RR = 4.65), respectively. In contrast, the relative risk of mortality continues to increase with age, with peaks at 95+ years (RR = 36.381) (**Supplementary Table 10**). The gender-stratified subgroup analysis (**Supplementary Fig. 3**) further reveals that females aged 65 and above systematically have higher risks of incidence, prevalence, and mortality compared to males (**Supplementary Table 11**).

The period effect analysis indicates a significant temporal cumulative effect of AF/AFL burden from 1992 to 2017, with the relative risks of incidence, prevalence, and mortality increasing by 170%, 210%, and 300%, respectively, reaching a historical peak in 2017 (**Supplementary Table 10**). It is noteworthy that this period-driven risk increase does not show gender disparity (**Supplementary Table 11**).

The cohort effect analysis reveals that earlier birth cohorts (e.g., the 1897–1901 cohort) have a higher exposure to AF/AFL risk compared to later birth cohorts (e.g., the 1987–1991 cohort), with the RR values showing a stepwise decline across generations (**Supplementary Table 10**). The gender-specific cohort patterns indicate that females born in multiple cohorts between 1897 and 1946 consistently have higher risks of incidence (up to the 1937–1941 cohort), prevalence (up to the 1922–1926 cohort), and mortality (up to the 1942–1946 cohort) compared to their male counterparts in the same periods (**Supplementary Table 11**).

### 3.7 Decomposition Analysis on AF/AFL DALYs

**Fig. 7.**
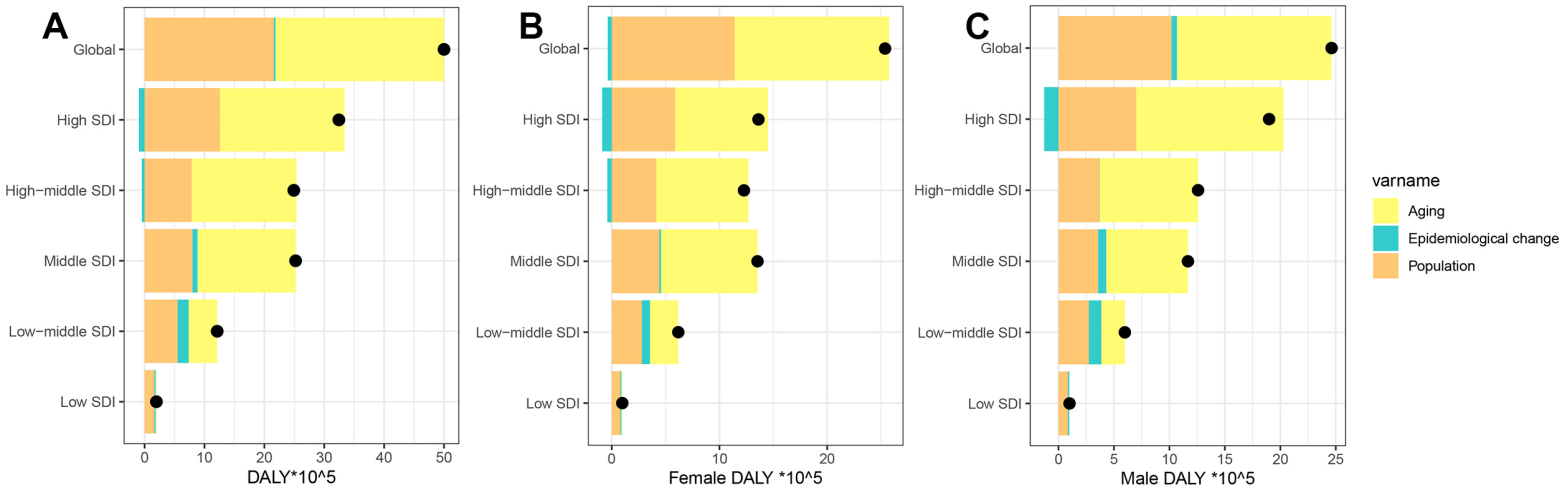
**Temporal trends of AF/AFL DALYs attributable to population-level determinants by gender and SDI quintile, globally and regionally, from 1990 to 2021**. (A) Both, (B) Female, (C) Male. The black dot represents the overall value of change contributed by all 3 components. For each component, the magnitude of a positive value indicates a corresponding increase in AF/AFL DALYs attributed to the component; the magnitude of a negative value indicates a corresponding decrease in AF/AFL DALYs attributed to that same component.

During the last 32 years, there has been a significant rise in global DALYs attributed to AF/AFL, with the most pronounced increase seen in regions with high SDI (Fig. 7). Decomposition analysis indicates that the primary factors driving this global increase are an aging population (accounting for 56.31%) and population growth (43.17%). The effects of aging, population growth, and epidemiological shifts on the growth of DALYs are particularly notable in high-middle SDI regions (70.27%), low SDI regions (83.62%), and low-middle SDI regions (15%). Gender difference analysis further reveals that males are significantly more affected by epidemiological factors than females (1.98% vs. –1.47%) (**Supplementary Table 12**). At the national/regional level, the interaction between population structure and disease prevalence patterns shows high heterogeneity ageing, population growth and epidemiology contribute most in Turkmenistan (1915.93%), Paraguay (1084.04%) and Nauru (360.54%) (**Supplementary Table 13**).

### 3.8 Cross-national AF/AFL Health Inequality

**Fig. 8.**
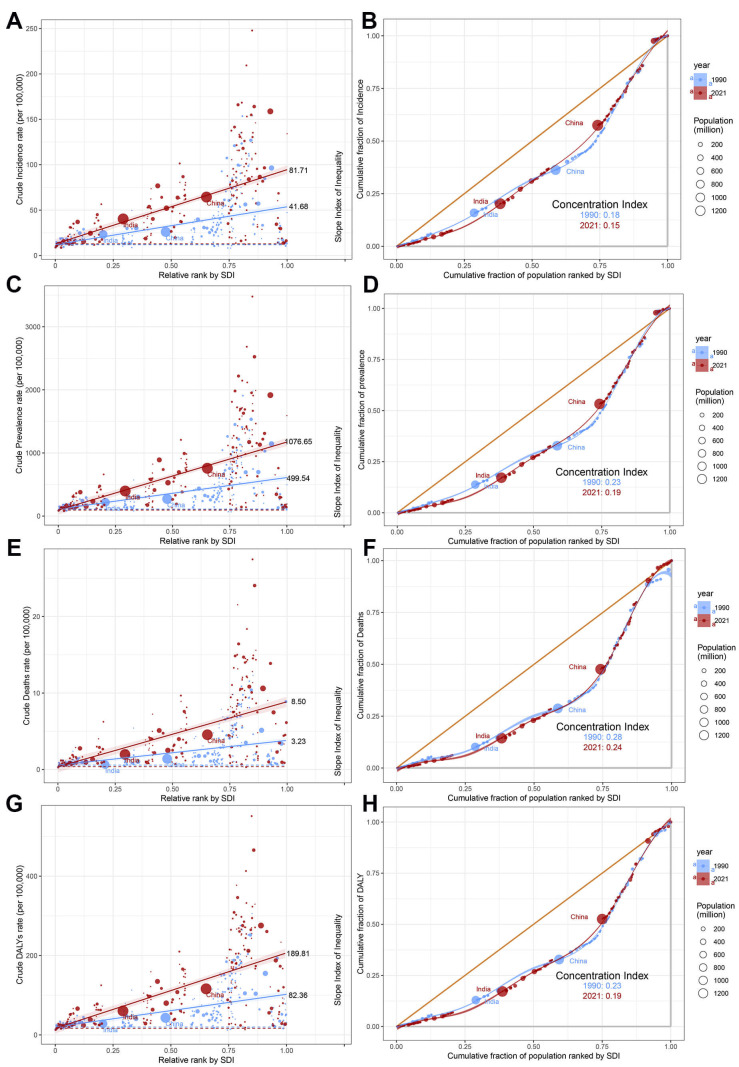
**Slope indices inequality and concentration indices of AF/AFL in 1990 and 2019**. (A) Slope Index of Inequality for incidence. (B) Concentration indices for incidence. (C) Slope Index of Inequality for prevalence. (D) Concentration indices for prevalence. (E) Slope Index of Inequality for death. (F) Concentration indices for death. (G) Slope Index of Inequality for DALYs. (H) Concentration indices for DALYs. Each country or region is represented by a solid dot, with larger dots indicating a higher population.

Worldwide, substantial disparities persist in AF/AFL burden when measured through both absolute (Slope Index of Inequality, SII) and relative (concentration index) metrics, with high-SDI countries experiencing disproportionately greater burden over time (Fig. 8). Comparative analysis between 1990 and 2021 reveals divergent trends, SII showed marked increases across all indicators-incidence (41.68 vs. 81.71), prevalence (499.54 vs. 1076.65), mortality (3.23 vs. 8.50), and DALYs (82.36 vs. 189.81), and the concentration index demonstrated consistent decreases for the same metrics: incidence (0.18 vs. 0.15), prevalence (0.23 vs. 0.19), mortality (0.28 vs. 0.24), and DALYs (0.22 vs. 0.19) (Fig. 8). This indicates that the absolute health inequality of the AF/AFL burden has increased, but the relative inequality has actually diminished.

### 3.9 Predictive Analysis on AF/AFL Burden to 2036

**Fig. 9.**
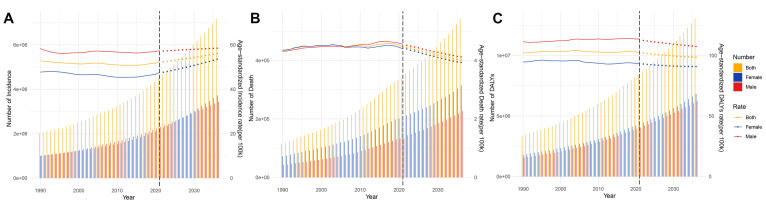
**Projects the ASRs and numbers of AF/AFL by gender from 1990 to 2036 based on the BAPC model**. (A) Incidence. (B) Deaths. (C) DALYs. BAPC, Bayesian age–period–cohort.

Global AF/AFL burden from 2022 to 2036 reveals persistent growth in absolute disease burden. Specifically, the ASIR demonstrates a progressive increase from 52.36 to 56.07 per 100,000 population, with distinct gender divergence—female ASIR shows accelerated growth compared to males. Despite current male predominance in ASIR (58.53 vs. 53.57 in females), case number projections indicate an impending gender crossover around 2028 (2,808,185 male cases vs. 2,819,358 female cases) (Fig. 9A). Concurrently, while ASDR exhibit overall decline, male mortality burden remains significantly elevated (4.12 vs. 3.93 in female). It is significant to observe that the absolute number of female deaths will continue to be high (227,048 vs. 314,089) (Fig. 9B). The ASDARs show consistent decline, with male ASDAR remaining significantly higher than females (107.45 vs. 90.87 per 100,000), while the absolute number of female DALYs will always be dominant (6,206,523 vs. 6,817,798) (Fig. 9C, **Supplementary Fig. 4**). These results highlight two key public health contradictions: the paradox of improving standardized rates versus growing absolute burden, and the decoupled characteristics of disease risk across gender dimensions (**Supplementary Table 14**).

## 4. Discussion

This study conducted a multi-layered spatiotemporal analysis of the evolving disease burden of AF/AFL. The research indicates that in 2021, there were 4.48 million new cases of AF/AFL and 330,000 deaths worldwide. Six regions, including Central Asia and Eastern Europe, showed an upward trend in the ASR for all four indicators, necessitating heightened vigilance regarding disease dynamics in these areas. Sweden had the highest ASR for incidence and prevalence, potentially linked to an aging population and increased life expectancy [15]. China led the world in the number of cases for incidence, prevalence, deaths, and DALYs, a phenomenon likely closely related to its large population base. Among the five SDI regions, the high SDI region had significantly higher case numbers and ASRs for all four indicators compared to the low SDI region. This disparity reflects a gap in epidemiological surveillance capacity between the two regions—he low SDI region may underreport cases due to insufficient diagnostic coverage, while the high SDI region, equipped with advanced technologies such as dynamic electrocardiogram monitoring and wearable devices, has significantly enhanced the detection rate of paroxysmal or asymptomatic subclinical cases. Prediction models suggested that the absolute number of cases will continue to grow from 2022 to 2036. These findings indicate an urgent need to establish a three-tier prevention system in response to the shift of the AF/AFL disease spectrum from hidden to overt: strengthening community-based control of cardiovascular risk factors at the primary prevention level; promoting AI-based AF screening technologies at the secondary prevention level; and improving the quality control system for anticoagulation therapy at the tertiary management level. These strategies are crucial for alleviating the pressure of misallocated medical resources and reducing the risk of complications such as stroke.

### 4.1 Joinpoint Regression

To analyze the overall trends of AF/AFL burden, we divided the study period into multiple sub-periods. This approach allowed us to capture detailed trajectories of change. Surprisingly, the ASR of incidence and prevalence showed a rapid upward trend from 2019 to 2021. This finding serves as a warning that the development of prevention and management strategies for AF/AFL remains an urgent task. The temporal trends of disease burden are influenced by changes in the occurrence and progression of the disease, as well as by how governments and individuals respond to these changes. Aging, as the most prominent risk factor for AF/AFL, has a particularly significant impact. Historically, the launch of “The International Year of Older Persons 1999” on October 1, 1998, marked the official entry into a new era of longevity and comprehensive aging [16]. This major shift may be an important reason for the rapid increase in the incidence of ASR and prevalence of AF/AFL from 2001 to 2004. Improvements in medical conditions and diagnostic technologies have also had a significant impact on the changes in the AF/AFL disease burden. A study published in The Lancet in July 2015 found that with these advancements, more patients with atrial fibrillation are being detected and diagnosed earlier [17], which may explain the increase in AF/AFL cases after 2015. Additionally, in the field of atrial fibrillation management, the comprehensive management pathway “Atrial Fibrillation Better Care” (ABC), first proposed in 2017, marks another important milestone [18]. This pathway has been confirmed by numerous clinical studies to provide significant clinical benefits [19]. It may be one of the key factors contributing to the decline in AF/AFL mortality since 2018.

### 4.2 Cohort Effects

Utilizing the APC model, this study examined the epidemiological trends of AF/AFL, identifying a pronounced age-related trend in its risk of incidence and prevalence. The age-stratified analysis demonstrated a steady rise in relative risk from adulthood, peaking twice in the 80–89 age group, and then decreasing among individuals aged 95 years and older. This non-linear age-risk trajectory not only reflects the dominant role of age-related pathophysiological mechanisms but also suggests the presence of potentially unrecognized protective factors in the ultra-elderly population. These findings emphasize the need to optimize the allocation of targeted intervention measures based on the risk factors patterns across different age groups, particularly focusing on the peak risk age windows. Notably, the relative risk of incidence exceeded the baseline value (RR >1) in males aged 55–59 and females aged 60–64, indicating the necessity for enhanced health interventions in the middle age populations [20]. Although lifelong risk exposure related to aging and cardiac remodeling remain the core pathogenic mechanisms [21], the decline in risk among the ultra-elderly (≥95 years) may be attributed to survival selection effects (i.e., cohort bias due to premature death of high-risk individuals) or insufficient diagnostic sensitivity, a phenomenon that requires validation through longitudinal cohort studies. The period for the relative risk of AF/AFL disease burden showed a continuous increasing trend, which may reflect two driving factors: the long-term accumulation of cardiovascular risk factors at the population level (such as hypertension and obesity), and the upgrading of disease registration systems and diagnostic technologies globally [2]. GBD 2021 data further indicate that high-income countries significantly improved case detection rates through the enhancement of medical infrastructure, objectively amplifying the statistical estimation of their disease burden. Cohort effect comparisons revealed that recent birth cohorts (e.g., 1987–1991) had significantly lower disease risk compared to earlier cohorts (e.g., 1897–1901), possibly due to generational differences—where the former was exposed to more severe cardiovascular risk factors throughout their lifetime, while the latter benefited from the widespread adoption of public health interventions and advancements in medical technology. These results offer crucial insights for the formulation of age-specific preventive and control measures and the enhancement of disease surveillance systems.

### 4.3 Gender Disparities

After the age of 65, females have a higher incidence, prevalence, and mortality rates of AF/AFL than males, which may be due to several reasons: hormonal changes and loss of cardiovascular adaptation to estrogen protection: postmenopausal females experience a significant decline in estrogen levels, losing its protective effects on vascular endothelial function, lipid metabolism, and the inflammatory response, leading to increased risks of atherosclerosis, left atrial fibrosis, and electrical remodeling, thereby promoting the occurrence of atrial fibrillation [22]. Differences in progesterone and androgen levels: females have lower androgen levels, which may affect myocardial electrical stability, while the potential arrhythmogenic effects of androgens in men may decrease in old age, highlighting gender differences [23]. Hypertension and obesity: the prevalence of hypertension in elderly females is higher than in males, and females are more sensitive to hypertension (atrial fibrillation risk increases more significantly at the same blood pressure level) [24]; differences in visceral fat distribution (women are more prone to abdominal fat accumulation) may exacerbate atrial remodeling. Diabetes and thyroid diseases: females have a higher risk of diabetes combined with atrial fibrillation than males [25], and thyroid dysfunction (such as hyperthyroidism) is more common in females [26], both of which are independent risk factors for atrial fibrillation. Atypical symptoms and delayed diagnosis: females with atrial fibrillation more often present with fatigue, dizziness, or are asymptomatic, while males are more likely to experience typical symptoms like palpitations, leading to delayed diagnosis in females (average delay of 1–2 years) and increased risk of complications such as thromboembolism [27]. Treatment differences and drug responses: Insufficient anticoagulation therapy: females have a higher risk of bleeding (especially intracranial hemorrhage) when using warfarin or new oral anticoagulants (NOACs), leading to more conservative prescribing by doctors and increased stroke risk due to undertreatment [28]. Low acceptance of invasive treatments: females are less likely to undergo catheter ablation (possibly due to concerns about complications or referral bias), and may have higher recurrence rates postoperatively (related to smaller atrial size and different degrees of fibrosis) [29]. Differences in cardiac structure and electrophysiology: Atrial remodeling characteristics: females have smaller left atrial diameters but higher stiffness, are prone to reentrant arrhythmias [30]; differences in ion channel expression (such as potassium channels) may increase female’s sensitivity to maintaining atrial fibrillation [31]. Autonomic nervous system influence: females have higher vagal tone, which may make them more susceptible to triggering atrial fibrillation in certain situations (such as at night or after meals) [32]. Social behavior and healthcare resource utilization: Inadequate comorbidity management: elderly females are more likely to live alone and may have poorer long-term management compliance for comorbidities such as hypertension and diabetes [33]. Heart failure comorbidity: females with atrial fibrillation have a higher proportion of heart failure, and female heart failure is predominantly characterized by preserved ejection fraction (HFpEF), with limited treatment options and poorer prognosis [34]. The higher risk of atrial fibrillation in elderly females compared to males is the result of a combination of physiological, pathological, and social factors. Targeted strategies should include: strengthening cardiovascular risk management in postmenopausal females, promoting screening for asymptomatic atrial fibrillation, optimizing anticoagulation therapy decisions (such as gender-specific dose adjustments), and improving the accessibility of invasive treatments. Future research needs to further reveal gender-specific mechanisms of atrial fibrillation to guide precision medicine. Additionally, in earlier birth cohorts, females had higher incidence, prevalence, and mortality than males, which may be related to survival bias. Females typically have a longer life expectancy than males and are more likely to survive to the age when atrial fibrillation is more prevalent (over 80 years), leading to higher prevalence statistics [35].

### 4.4 Regional Inequalities

The disease burden of AF/AFL exhibits significant geographical global heterogeneity. Through a quantitative examination of the cross-national disparities in the AF/AFL burden along various SDI levels, we can clarify the distribution patterns of the burden and accurately identify countries in urgent need of improving AF/AFL prevention and control measures. Traditionally, it was assumed that individuals residing in nations with elevated SDI levels possessed enhanced access to superior healthcare services, therefore, they might experience a comparatively reduced disease burden [36]. However, our observations reveal an unusual phenomenon: the AF/AFL burden is predominantly concentrated in high SDI countries, which is consistent with previous research findings [37]. This inequality may stem from three aspects: First, high SDI countries may experience a “survivor effect”, where individuals live longer and thus have more opportunities to be diagnosed with AF/AFL or suffer from its severe sequelae [38]. Second, in low SDI countries, due to relatively weak healthcare systems and limited availability of electrocardiograms, the diagnosis rate of AF/AFL may be lower, and individuals may die from other diseases before reaching the age of developing AF/AFL [39]. Furthermore, while residents in high SDI countries have extended their life expectancy to the age range where AF/AFL is more prevalent, their healthcare systems have not simultaneously matched the accessibility of standardized diagnostic and treatment pathways and advanced surgical interventions. Additionally, it is noteworthy that 10%–40% of atrial fibrillation patients do not exhibit obvious symptoms [40], a proportion that varies with increased public awareness, different types of atrial fibrillation, and updates in detection methodology. When discussing the phenomenon of AF/AFL mortality being concentrated in high SDI countries, we must be cautious of the ecological fallacy. The ecological fallacy refers to the mistake of drawing conclusions about individual behavior based on group data, while ignoring the differences between individuals. For example, at the national level, residents in affluent areas may have a longer average life expectancy [41], but inferring that every individual in affluent areas is healthier than those in poor areas is simplistic. This conclusion does not consider that there may be individuals with poor health even in affluent areas; similarly, there may be very healthy individuals in poor areas.

When exploring the relationship between AF/AFL-related mortality and SDI, we uncovered a revealing insight: higher income does not necessarily equate to a higher likelihood of dying from AF/AFL-related complications. In fact, evidence suggests that at the individual level, this correlation may be the opposite [42]. Our proposed explanation primarily focuses on the national level, such as the survivor effect mentioned above. The divergence between absolute and relative inequality trends further highlights the complex interplay of demographic and technological factors. Additionally, another explanation is the observed divergence between absolute and relative health inequalities—evidenced by rising SII alongside declining CI—which can be attributed to the differential drivers of disease burden across SDI strata. While high-SDI countries exhibit slower growth in ASR due to advanced healthcare systems, their aging populations and stable diagnostic capacities lead to gradual increases in absolute case numbers. Conversely, low-SDI regions experience rapid population expansion coupled with delayed epidemiological transition, resulting in accelerated growth of absolute burden. Mathematically, the decline in CI reflects a reduction in the proportional concentration of AF/AFL burden among high-SDI populations. As low-SDI countries account for an increasing share of global cases, the relative distribution becomes less skewed, even as absolute disparities widen. This paradox underscores the limitations of relying on a single inequality metric: while SII highlights urgent needs for resource allocation to address expanding case numbers in low-SDI regions, CI’s improvement signals progress in reducing the relative disadvantage of these populations. Additionally, another explanation is that in high-income countries, certain risky behaviors (such as poor dietary habits) may simultaneously lead to obesity, sleep apnea, and diabetes, which are risk factors for AF/AFL. These factors are interrelated and collectively contribute to the occurrence and progression of AF/AFL [1]. However, this relationship at the national level does not necessarily hold true at the individual level. High-income individuals within a country may be more effective at avoiding risky behaviors that lead to AF/AFL-related complications [42]. Since 1990, despite significant advancements in the management of AF/AFL, the global rise in age-standardized mortality rates indicates that AF/AFL-related mortality may be an inevitable consequence of improved survival rates for other diseases. Although our data have been adjusted for age, they may not fully capture the impact of comorbidities related to aging and the survival rates of other diseases. In less developed countries, these diseases may be more common causes of death. For example, in developed countries, cancer patients undergoing cardiotoxic chemotherapy may experience myocardial damage and an AF/AFL-related death. From a national perspective, while cancer mortality rates have declined, AF/AFL-related mortality rates have shown an upward trend.

### 4.5 Predictive AF/AFL Burden

Based on disease burden prediction models and decomposition analysis, this study reveals the following core conclusions: Disease Evolution Trends: By 2036, the ASR for AF/AFL incidence is expected to continue increasing, while the ASR for mortality and DALYs may show a marginal decline. However, it is noteworthy that the absolute number of cases for the incidence, mortality, and DALYs will all significantly increase, reflecting a sustained exacerbation of the actual public health burden of the disease. Analysis of Driving Factors: Through the application of a decomposition model for data spanning from 1990 to 2021, our investigation revealed that the worldwide rise in case numbers is primarily due to population expansion and the advancing age of the population. This dual demographic driver effect will continue to dominate the evolution of the disease burden over the next 15 years. Policy Intervention Directions: The study emphasizes the need for countries to establish dynamic response mechanisms to address two core challenges: first, accelerating the development of age-friendly healthcare systems, with a focus on strengthening cardiovascular health management capabilities for the elderly; and second, optimizing the allocation of medical resources based on population structure prediction data, particularly enhancing early screening and tiered diagnosis and treatment capabilities for AF/AFL at the primary care level. These measures are strategically significant for alleviating the future pressure of chronic disease management.

### 4.6 Methodological Limitations

This study has several limitations. First, the GBD database lacks fine-grained classification of AF/AFL subtypes (such as paroxysmal, persistent, and permanent), which may weaken the impact of clinical heterogeneity on the assessment of disease burden. Second, the study did not deeply analyze the influence of intra-national health resource allocation disparities on disease distribution, which is crucial for revealing health equity. Third, data completeness issues due to underdiagnosis, misdiagnosis, and missing case registration systems in some regions (especially in less developed countries in Africa and Asia) could result in an underestimation of the disease burden, although statistical modeling has been used for correction. Fourth, the heterogeneity of cross-national data is prominent, with technical biases such as differences in diagnostic criteria, reporting delays, and classification errors being difficult to completely eliminate. Fifth, prediction model bias: The prediction models may overestimate the true disease burden in high-SDI regions. Advanced diagnostic technologies in these areas, such as ambulatory electrocardiogram monitoring, may enhance case detection rates; but they also can introduce detection bias by capturing subclinical or paroxysmal cases. As a result, sensitivity analyses suggest that the incidence of age-standardized rates (ASR) in high-SDI regions may be overestimated. Future research should incorporate the WHO Health Technology Access Index (HTAI) to correct for regional differences in the probability of detection. Sixth, sensitivity of decomposition analysis: The Das Gupta decomposition method assumes independent effects of aging, population growth, and epidemiological changes, which may underestimate the interaction effects in regions with extreme demographic shifts (such as Turkmenistan, where the contribution rate of aging reached 1915.93%). Such outliers indicate the limitations of the model in scenarios of rapid demographic transformation. It is recommended that subsequent research adopt nonlinear methods such as the Logarithmic Mean Divisia Index (LMDI) to optimize the decomposition logic. Seventh, prediction models based on historical trends and covariate parameters inevitably suffer from limitations in limitations due to the lag in the GBD data update cycle. Nevertheless, by integrating multi-layered methods including descriptive analysis, spatiotemporal trend modeling, multivariate decomposition, health equity assessment, and future forecasting, this study systematically reveals the epidemiological characteristics of AF/AFL. Our findings offer crucial insights for enhancing worldwide preventive and control measures, as well as for refining the allocation of medical resources.

## 5. Conclusions

The disease burden of AF/AFL, due to the continuous rise in the incidence, prevalence, mortality, and DALYs, has become an important public health challenge worldwide. Epidemiological analysis indicates significant heterogeneity in the AF/AFL burden among countries, with its growth trend primarily driven by multi-dimensional factors such as population expansion and an aging population, suggesting a potential exponential increase in case numbers over the next few decades. It is noteworthy that while high SDI countries have more comprehensive healthcare systems, they bear a disproportionately heavy disease burden relative to their population size, and the trend of SDI-related health inequalities across countries is widening annually. These findings not only reveal the dual dilemma faced in the prevention and control of AF/AFL—the global contradiction between surging cases and imbalanced resource allocation—but also provide critical insights for optimizing public health decision-making. There is an urgent need to establish a differentiated intervention framework, implementing precise strategies and dynamically adjusting healthcare resource allocation to address the specific needs of countries at different levels of development in disease management.

## Data Availability

The datasets analyzed during the current study are available in the https://vizhub.healthdata.org/gbd-results/.

## Abbreviations

AF, atrial fibrillation; AFL, atrial flutter; DALYs, disability-adjusted life years; ASR, age-standardized rate; CI, confidence interval; EAPC, estimated annual percentage change; SDI, socio-demographic index; UI, uncertainty interval; APC, annual percentage change; AAPC, average annual percentage change; APC, Age-Period-Cohort; RR, relative risk.

## Supplementary Material

Supplementary material associated with this article can be found, in the online version, at https://doi.org/10.31083/RCM45091.
