# Supplementary material for: Global Epidemiological Transition of Atrial Fibrillation/Flutter (1990–2021): Multidimensional Burden Dynamics and Socioeconomic Health Gradients Across 204 Countries and Territories
Source: Rev Cardiovasc Med. 2025 Dec 18;26(12):45091. doi: 10.31083/RCM45091 (PMC12781011; doi:10.31083/RCM45091)

A
Age−standardized incidence rate

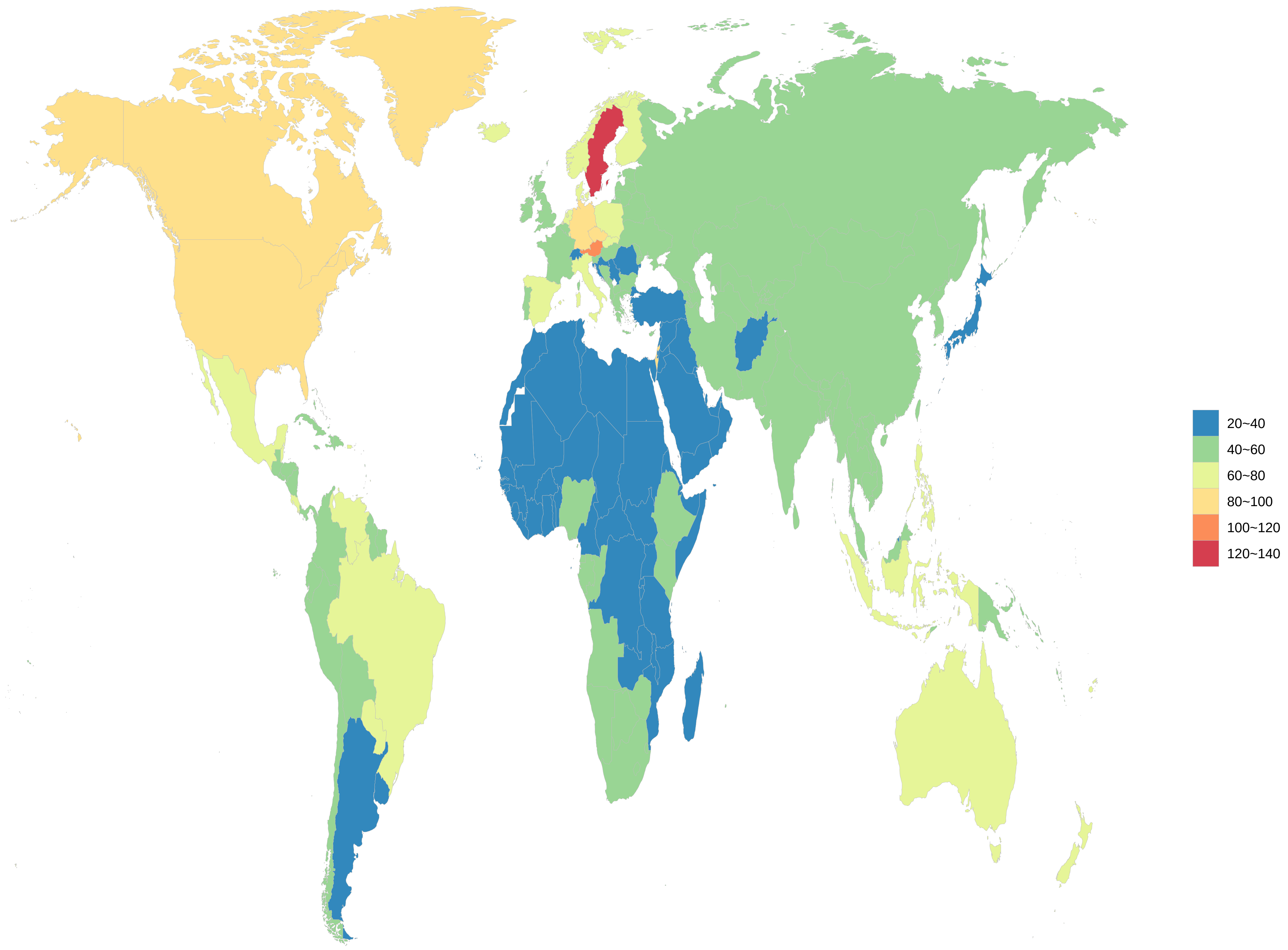

B
Age−standardized prevalence rate

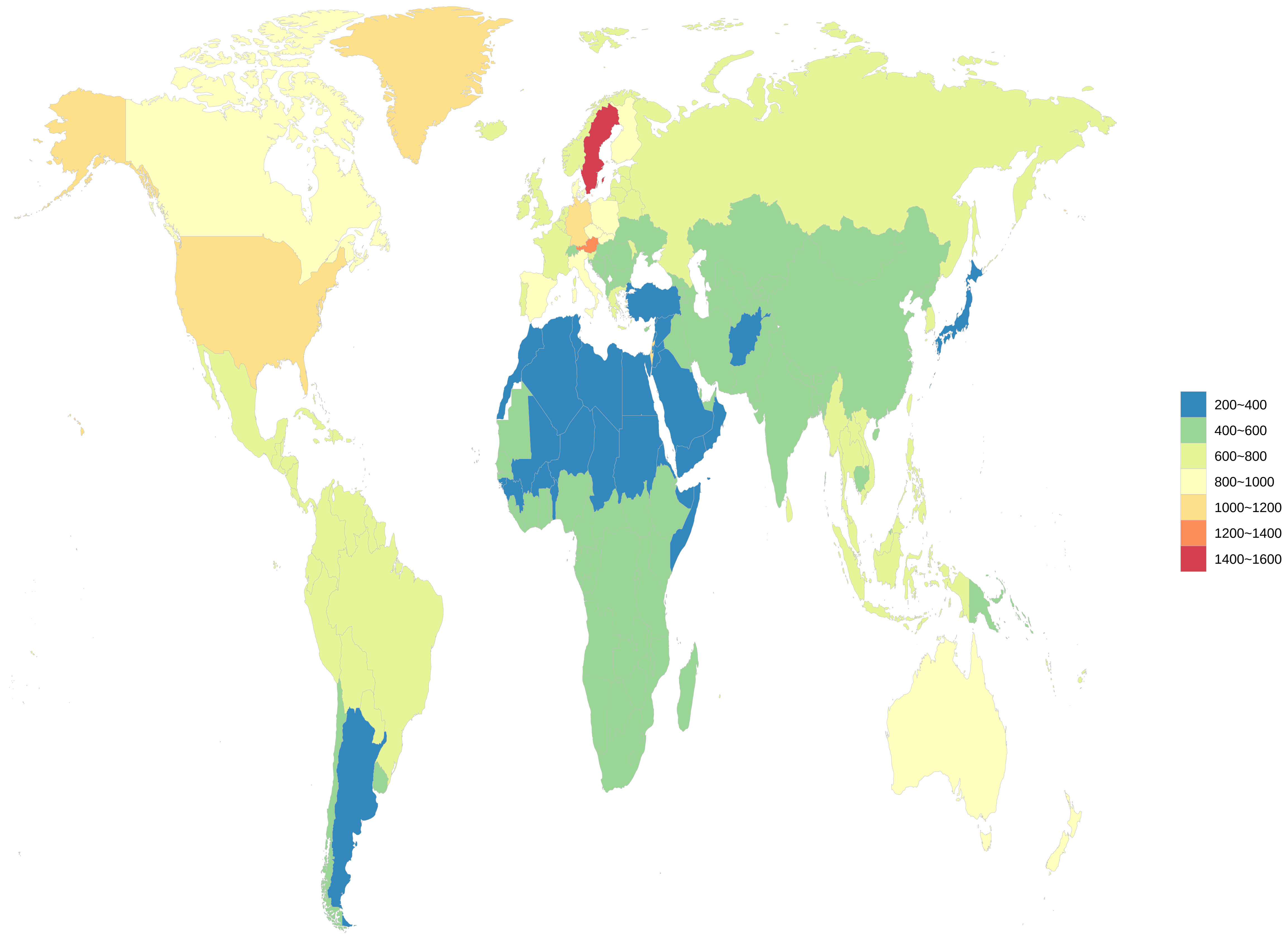

C
Age−standardized death rate

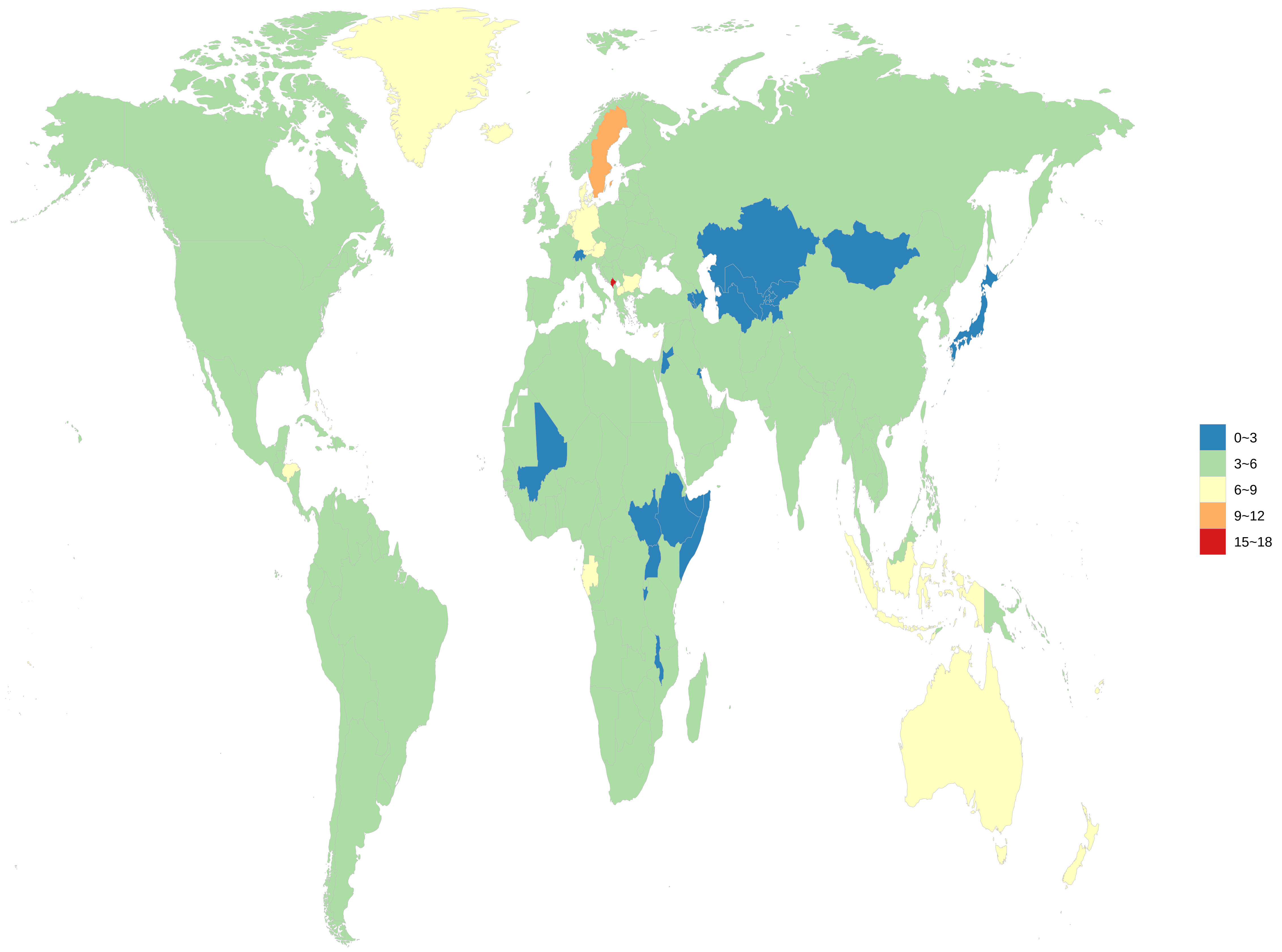

D
Age−standardized DALYs rate

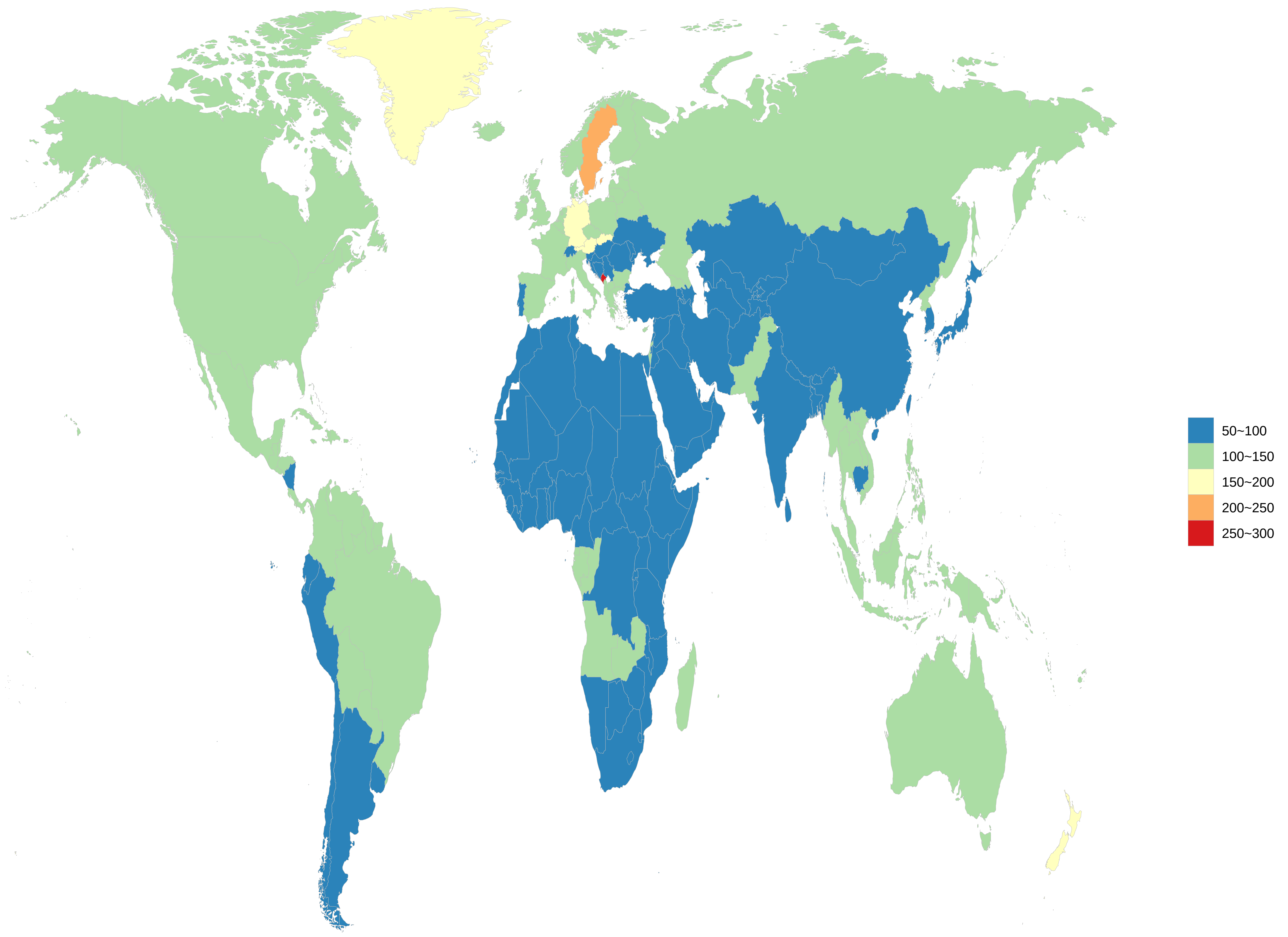

Supplement: Supplementary file 1 [file 2153-8174-26-12-45091-s1.zip › Supplementary Fig. 2.pdf]
