# Supplementary material for: Global Epidemiological Transition of Atrial Fibrillation/Flutter (1990–2021): Multidimensional Burden Dynamics and Socioeconomic Health Gradients Across 204 Countries and Territories
Source: Rev Cardiovasc Med. 2025 Dec 18;26(12):45091. doi: 10.31083/RCM45091 (PMC12781011; doi:10.31083/RCM45091)
Supplement: Supplementary file 1 [file 2153-8174-26-12-45091-s1.zip › Supplementary Material.docx]

Supplementary Table 1 Prevalence of AF/AFL Between 1990 and 2021 at the Global and Regional Level

| **Location** | **Prevalence in 1990** | | **Prevalence in 2021** | | **1990-2021 EAPC** |
| --- | --- | --- | --- | --- | --- |
|  | **Number(95%UI)** | **Rate(95%UI)** | **Number(95%UI)** | **Rate(95%UI)** | **EAPC 95%CI** |
| Global | 22214495 (17526215,28522555) | 616.58 (485.22,795.26) | 52552045 (43137876,64963854) | 620.51 (511.36,768.88) | "-0.01  (-0.04,0.01)" |
| Sex |  |  |  |  |  |
| Males | 11492470 (9169280,14778457) | 727.79 (578.43,929.67) | 27899046 (23026328,34646024) | 728.88 (601.91,895.81) | 0.05 (0.01,0.08) |
| Females | 10722025 (8387109,13889787) | 529.53 (413.11,690.99) | 24652999 (20031485,30931399) | 529.12 (430.79,663.14) | -0.11 (-0.14,0.07) |
| SDI region |  |  |  |  |  |
| High SDI | 8631675 (6800989,11018130) | 766.6 (608.09,974.16) | 17401068 (15107026,20202156) | 788.35 (690.97,910.9) | "0.01  (-0.04,0.06)" |
| High-middle SDI | 5443470 (4254251,7001406) | 587.77 (464.15,754.8) | 11539353 (9330859,14532463) | 581.39 (473.47,731.22) | -0.07(-0.11,0.03) |
| Middle SDI | 4673673 (3692430,6104440) | 537.07 (422.34,692.7) | 14670503 (11595532,19210983) | 579.06 (457.58,748.76) | "0.27  (0.23,0.3)" |
| Low-middle SDI | 2632144 (2062576,3472861) | 524.91 (409.36,683.63) | 6929826 (5413491,9033562) | 546.49 (425.68,711.16) | "0.14  (0.13,0.14)" |
| Low SDI | 806009 (629010,1060706) | 442.34 (345.89,576.61) | 1959344 (1535182,2587655) | 463.23 (362.02,602.71) | "0.16  (0.14,0.17)" |
| GBD region |  |  |  |  |  |
| Andean Latin America | 112466 (88857,146494) | 599.92 (471.7,773.74) | 371258 (290314,478820) | 647.36 (505.19,835.55) | "0.33  (0.29,0.37)" |
| Australasia | 208038 (190582,225387) | 885.17 (810.92,960.4) | 508534 (400838,650591) | 913.63 (725.73,1163.47) | "0.2  (0.14,0.25)" |
| Caribbean | 168732 (130817,218385) | 677.46 (527.46,877.57) | 367640 (289060,476682) | 678.59 (533.41,881.12) | "0  (-0.01,0.01)" |
| Central Asia | 222460 (171711,290357) | 513.48 (395.59,666.95) | 392829 (305783,509896) | 536.72 (410.76,698.74) | "0.14  (0.13,0.15)" |
| Central Europe | 901372 (699705,1154305) | 616.69 (478.07,790.3) | 1585129 (1281858,1949452) | 679 (554.31,827.23) | "0.07  (-0.01,0.15)" |
| Central Latin America | 524834 (413226,681703) | 702.85 (548.24,910.96) | 1711299 (1340757,2226156) | 707.02 (552.56,921.13) | "0.05  (0.04,0.07)" |
| Central Sub-Saharan Africa | 73688 (57051,95882) | 427.06 (330.92,558.11) | 182594 (142529,241993) | 424.57 (331.96,559.66) | -0.05(-0.06,0.04) |
| East Asia | 3359584 (2649140,4376801) | 462.71 (363.3,601.04) | 11215165 (8885909,14572495) | 526.44 (420.6,683.56) | "0.46  (0.36,0.55)" |
| Eastern Europe | 1601348 (1245865,2080668) | 584.41 (454.97,753.25) | 2327313 (1805306,3029889) | 648.92 (507,836.25) | "0.39  (0.32,0.47)" |
| Eastern Sub-Saharan Africa | 253297 (198232,333959) | 407.55 (320.71,534.37) | 645690 (507531,839253) | 448.76 (354.06,589.55) | "0.31  (0.27,0.35)" |
| High_income Asia Pacific | 1052102 (825876,1364552) | 533.61 (423.32,688.79) | 2165450 (1737905,2727227) | 465.22 (383.84,577.04) | -0.35(-0.57,0.12) |
| High_income North America | 3293625 (2543345,4240128) | 900.45 (701.18,1153.05) | 7106415 (6544296,7755026) | 1031.17 (952.3,1117.88) | "0.45  (0.4,0.51)" |
| North Africa And Middle East | 474266 (369341,613686) | 345.93 (267.05,451.62) | 1417367 (1140557,1788911) | 366.92 (291.62,468.4) | 0.1(0.07,0.14) |
| Oceania | 12769 (10040,16711) | 558.23 (439.17,726.94) | 34994 (27519,45179) | 578.44 (455.22,750.19) | "0.11  (0.09,0.12)" |
| South Asia | 2357498 (1827475,3099820) | 519.49 (401.88,683.87) | 6882583 (5295961,9026139) | 530.9 (410.19,696.69) | 0.09(0.08,0.1) |
| Southeast Asia | 1377746 (1089207,1799890) | 637.21 (502.03,823.86) | 3898608 (3091004,5118881) | 662.33 (519.61,854.36) | "0.15  (0.13,0.16)" |
| Southern Latin America | 212016 (165906,278675) | 475.26 (372.44,622.31) | 348813 (291741,425439) | 391.08 (328.22,476.31) | -0.7(-0.89,0.52) |
| Southern Sub-Saharan Africa | 121142 (94906,158838) | 510.12 (398.24,662.2) | 258590 (201941,342437) | 512.78 (398.19,671.03) | -0.01 (-0.02,0) |
| Tropical Latin America | 661730 (521046,861787) | 802.54 (631.12,1040.53) | 2004719 (1580577,2599905) | 794.78 (625.65,1025.18) | -0.07(-0.09,0.06) |
| Werstern Europe | 4930276 (3885223,6301409) | 817.69 (649.57,1042.91) | 8410140 (7063579,10013004) | 844.93 (717.15,992.46) | -0.03 (-0.11,0.05) |
| Werstern Sub-Saharan Africa | 295505 (230745,390715) | 393.31 (305.99,515.67) | 716915 (561805,945355) | 434.84 (339.54,567.05) | 0.36 (0.34,0.39) |

AF/AFL, Atrial fibrillation (AF)/atrial flutter (AFL); CI, confidence interval; EAPC, estimated annual percentage change; UI, uncertainty interval; SDI, socio-demographic index.

Supplementary Table 2 Death of AF/AFL Between 1990 and 2021 at the Global and Regional Level

| **Location** | **Death in 1990** | | **Death in 2021** | | **1990-2021 EAPC** |
| --- | --- | --- | --- | --- | --- |
|  | **Number(95%UI)** | **Rate(95%UI)** | **Number(95%UI)** | **Rate(95%UI)** | **EAPC_95%CI** |
| Global | 114540 (101326,127155) | 4.24 (3.69,4.71) | 338947 (288954,368613) | 4.36 (3.69,4.75) | 0.1(0.06,0.13) |
| Sex |  |  |  |  |  |
| Males | 42677 (37233,46516) | 4.2 (3.65,4.59) | 134700 (120296,145862) | 4.44 (3.94,4.81) | 0.21(0.18,0.24) |
| Females | 71862 (63274,81196) | 4.25 (3.66,4.79) | 204247 (167703,228405) | 4.29 (3.53,4.8) | 0.02(-0.02,0.06) |
| SDI region |  |  |  |  |  |
| High SDI | 50188 (44165,53072) | 4.74 (4.14,5.03) | 125622 (103230,137669) | 4.66 (3.88,5.08) | -0.01(-0.06,0.04) |
| High-middle SDI | 28089 (25067,30559) | 4.23 (3.69,4.63) | 79214 (67067,88012) | 4.29 (3.62,4.77) | 0.08(0.03,0.13) |
| Middle SDI | 22063 (19086,25973) | 4.18 (3.6,4.92) | 83853 (70992,94818) | 4.26 (3.57,4.83) | -0.06(-0.13,0.02) |
| Low-middle SDI | 10458 (7579,14095) | 3.07 (2.26,4.06) | 39263 (32069,46278) | 4.08 (3.34,4.81) | 1(0.91,1.08) |
| Low SDI | 3568 (2077,5030) | 3.05 (1.79,4.32) | 10623 (7284,13876) | 3.74 (2.57,4.9) | 0.87(0.63,1.12) |
| GBD region |  |  |  |  |  |
| Andean Latin America | 740 (634,855) | 4.61 (3.97,5.3) | 2079 (1699,2494) | 3.81 (3.11,4.57) | -0.78(-0.94,0.61) |
| Australasia  Caribbean | 1436 (1281,1525)  1098 (985,1194) | 6.99 (6.16,7.45)  5.59 (4.99,6.07) | 4337 (3529,4789)  2731 (2349,3048) | 6.58 (5.39,7.25)  4.86 (4.2,5.42) | 0(-0.14,0.14)  -0.42(-0.51,0.33) |
| Central Asia | 684 (596,799) | 1.87 (1.61,2.2) | 1386 (1241,1519) | 2.37 (2.09,2.6) | 0.69(0.43,0.95) |
| Central Europe | 5931 (5508,6253) | 5.1 (4.66,5.4) | 11104 (9859,11914) | 4.55 (4.03,4.88) | -0.21(-0.41,0.02) |
| Central Latin America | 2816 (2613,2927) | 4.98 (4.56,5.21) | 10354 (8980,11386) | 4.55 (3.95,5.01) | -0.32(-0.38,0.26) |
| Central Sub-Saharan Africa | 439 (278,633) | 4.11 (2.55,5.93) | 1311 (886,1982) | 4.67 (3.16,7.09) | 0.36(0.19,0.54) |
| East Asia | 17272 (14013,21292) | 4.94 (3.92,6.07) | 67666 (54232,80814) | 4.3 (3.42,5.18) | -0.63(-0.8,0.45) |
| Eastern Europe | 8133 (7308,8953) | 3.87 (3.44,4.3) | 15697 (14042,17056) | 4.33 (3.87,4.71) | 0.26(0.15,0.37) |
| Eastern Sub-Saharan Africa | 1263 (687,1765) | 3.17 (1.73,4.55) | 3166 (1927,4731) | 3.28 (1.97,4.89) | 0.06(-0.08,0.2) |
| High_income Asia Pacific | 5018 (4465,5373) | 3.06 (2.68,3.3) | 17112 (13415,19331) | 2.46 (2.01,2.74) | -1.27(-1.61,0.93) |
| High_income North America | 14544 (12556,15549) | 3.96 (3.41,4.24) | 39066 (32116,42759) | 5.15 (4.27,5.61) | 0.79(0.72,0.87) |
| North Africa And Middle East | 3374 (2613,4241) | 3.5 (2.68,4.4) | 11182 (9280,12594) | 3.92 (3.19,4.44) | 0.53(0.34,0.72) |
| Oceania | 65 (44,82) | 4.48 (3.27,5.61) | 185 (132,238) | 4.29 (3.16,5.46) | -0.19(-0.25,0.13) |
| South Asia | 7594 (4655,11542) | 2.38 (1.46,3.62) | 36165 (27041,46073) | 3.69 (2.75,4.71) | 1.58(1.38,1.78) |
| Southeast Asia | 6120 (5116,7566) | 4.24 (3.5,5.3) | 22401 (18974,25759) | 5.27 (4.39,6.12) | 0.61(0.49,0.74) |
| Southern Latin America | 1317 (1195,1407) | 3.61 (3.22,3.88) | 3299 (2853,3562) | 3.53 (3.06,3.81) | 0.74(0.38,1.11) |
| Southern Sub-Saharan Africa | 511 (417,626) | 2.82 (2.26,3.48) | 1413 (1228,1551) | 4 (3.39,4.44) | 1.14(0.82,1.46) |
| Tropical Latin America | 2727 (2419,2896) | 4.99 (4.29,5.36) | 11540 (9640,12679) | 4.86 (4.05,5.34) | -0.12(-0.21,0.03) |
| Werstern Europe | 31335 (27682,33261) | 5.43 (4.75,5.77) | 72184 (58846,79292) | 5.52 (4.56,6.04) | 0.31(0.24,0.38) |
| Werstern Sub-Saharan Africa | 2121 (1585,2724) | 4.51 (3.37,5.78) | 4568 (3662,5235) | 4.46 (3.65,5.13) | -0.23(-0.32,0.14) |

AF/AFL, Atrial fibrillation (AF)/atrial flutter (AFL); CI, confidence interval; EAPC, estimated annual percentage change; UI, uncertainty interval; SDI, socio-demographic index.

Supplementary Table 3 DALYs of AF/AFL Between 1990 and 2021 at the Global and Regional Level

| **Location** | **DALYs in 1990** | | **DALYs in 2021** | | **1990-2021 EAPC** |
| --- | --- | --- | --- | --- | --- |
|  | **Number(95%UI)** | **Rate(95%UI)** | **Number(95%UI)** | **Rate(95%UI)** | **EAPC_95%CI** |
| Global | 3358708 (2715430,4141735) | 100.81 (82.82,122.62) | 8358894 (6970688,10133489) | 101.4 (84.89,122.41) | 0 (-0.02,0.02) |
| Sex |  |  |  |  |  |
| Males | 1569927 (1231979,1964377) | 109.93 (88.5,134.72) | 4032121 (3310673,4901830) | 112.05 (93.3,135.28) | 0.09(0.08,0.11) |
| Females | 1788781 (1483399,2202805) | 93.29 (77.72,113.71) | 4326773 (3603522,5211317) | 92.24 (76.84,111.24) | -0.1(-0.12,0.07) |
| SDI region |  |  |  |  |  |
| High SDI | 1327542 (1075909,1630068) | 119.83 (97.47,146.18) | 2788952 (2345598,3317970) | 118.88 (99.51,141.23) | -0.05(-0.09,0.01) |
| High-middle SDI | 827405 (668171,1021693) | 98.39 (80.58,119.12) | 1871378 (1545511,2273392) | 96.58 (79.91,116.81) | -0.07(-0.11,0.03) |
| Middle SDI | 703287 (554668,874920) | 92.91 (76.32,114.46) | 2256889 (1828561,2776292) | 96.28 (79.25,117.4) | 0.06 (0.02,0.1) |
| Low-middle SDI | 371209 (283400,482922) | 80.09 (61.96,102.79) | 1110930 (893165,1385764) | 94.25 (76.45,116.71) | 0.55 (0.52,0.59) |
| Low SDI | 124604 (87734,165979) | 75 (53.23,99.5) | 321963 (242299,416819) | 84.52 (63.65,108.24) | 0.48 (0.36,0.6) |
| GBD region |  |  |  |  |  |
| Andean Latin America | 19240 (15208,23493) | 106.95 (85.46,130.46) | 56583 (44608,69532) | 99.93 (78.68,122.31) | -0.27(-0.36,0.19) |
| Australasia | 34608 (29287,40036) | 153.78 (131.23,177.04) | 87583 (72120,105463) | 147.83 (121.51,179.59) | 0 (-0.07,0.07) |
| Caribbean | 28429 (23264,34542) | 122.84 (102.16,147.29) | 63053 (51870,76838) | 115.24 (94.57,140.74) | -0.19(-0.22,0.15) |
| Central Asia | 27964 (21497,36702) | 66.28 (51.57,86.71) | 52600 (41314,67337) | 75 (59.9,95.5) | 0.36 (0.27,0.45) |
| Central Europe | 157482 (132449,188984) | 115.47 (98.44,136.8) | 265917 (225064,316547) | 112.31 (94.56,134.27) | -0.11 (-0.25,0.02) |
| Central Latin America | 80232 (64872,98986) | 115.99 (95.04,141.04) | 269931 (218012,327388) | 113.57 (91.93,137.77) | -0.08(-0.1,0.06) |
| Central Sub-Saharan Africa | 14316 (10216,19085) | 91.59 (64.75,123.72) | 37999 (27202,52014) | 98.45 (70.85,138.18) | 0.19 (0.09,0.3) |
| East Asia | 533837 (416978,668275) | 93.83 (76.48,115.88) | 1723468 (1364785,2143575) | 89.83 (72.42,109.59) | -0.23(-0.32,0.13) |
| Eastern Europe | 242292 (195940,303226) | 95.36 (78.74,118) | 388580 (318353,470379) | 107.73 (88.25,130.33) | 0.36 (0.27,0.44) |
| Eastern Sub-Saharan Africa | 42708 (29866,56564) | 75.84 (52,99.79) | 103373 (74839,140257) | 79.25 (57.05,108.27) | 0.1 (0.01,0.19) |
| High_income Asia Pacific | 156515 (125660,193831) | 83.08 (67.38,102.03) | 364512 (301067,443767) | 69.03 (56.4,84.74) | -0.84(-1.05,0.63) |
| High_income North America | 443449 (346576,559226) | 121.41 (94.78,152.92) | 1015978 (854240,1202900) | 144.37 (121,171.39) | 0.54 (0.5,0.58) |
| North Africa And Middle East | 88830 (69529,110179) | 71.6 (56.5,88.83) | 265649 (218559,309833) | 76.1 (63.33,88.63) | 0.23(0.12,0.33) |
| Oceania | 2441 (1825,3093) | 110.29 (85.01,137.38) | 6606 (5044,8584) | 110.15 (86.19,141.7) | -0.02 (-0.05,0) |
| South Asia | 311117 (228511,416726) | 71.78 (52.92,96.69) | 1071260 (820127,1366680) | 88.3 (68.02,111.45) | 0.74(0.66,0.82) |
| Southeast Asia | 200296 (158700,252961) | 102.35 (82.98,128.37) | 612969 (501403,745497) | 115.4 (95.88,139.11) | 0.34 (0.27,0.41) |
| Southern Latin America | 34600 (28133,42981) | 82.4 (67.73,101.03) | 67844 (58167,78537) | 74.67 (63.84,86.8) | 0.14 (-0.08,0.36) |
| Southern Sub-Saharan Africa | 17046 (13347,21744) | 75.25 (59.56,95.82) | 41787 (34114,50581) | 90.4 (74.56,108.99) | 0.62 (0.44,0.8) |
| Tropical Latin America | 90777 (71331,113772) | 122.82 (99.71,151.03) | 304099 (245416,375972) | 122.75 (99.62,151.65) | -0.04 (-0.09,0) |
| Werstern Europe | 779577 (634739,949783) | 130.74 (106.98,159.03) | 1440284 (1208224,1709735) | 131.15 (108.91,156.67) | 0.08 (0.03,0.12) |

AF/AFL, Atrial fibrillation (AF)/atrial flutter (AFL); CI, confidence interval; EAPC, estimated annual percentage change; UI, uncertainty interval; SDI, socio-demographic index.

Supplementary Table 4 Incidence of AF/AFL Between 1990 and 2021 in 204 countries

| **Location** | **Incidence in 1990** | | **Incidence in 2021** | | **1990-2021 EAPC** | **1990-2021 change** |
| --- | --- | --- | --- | --- | --- | --- |
| United Arab Emirates | 155 (120,199) | 37.02 (27.3,49.36) | 1662 (1236,2173) | 39.65 (29.19,52.8) | 0.16 (-0.04,0.37) | 971.25 |
| Qatar | 36 (29,46) | 35.2 (25.6,46.99) | 366 (283,474) | 37.6 (27.56,50.65) | -0.19 (-0.22,-0.16) | 906.6 |
| Jordan | 386 (295,505) | 34.36 (25.04,46.19) | 2472 (1878,3282) | 37 (27.48,49.67) | -0.68 (-0.83,-0.53) | 540.8 |
| Bahrain | 53 (41,70) | 35.35 (26.17,47.72) | 297 (229,382) | 36.29 (26.61,48.17) | -0.13 (-0.14,-0.11) | 456.27 |
| Kuwait | 188 (147,241) | 35.46 (25.71,47.38) | 1013 (804,1301) | 37.87 (27.94,50.89) | 0.09 (0.08,0.1) | 438.71 |
| Djibouti | 43 (34,56) | 34.83 (26.74,46.37) | 224 (175,293) | 37.38 (28.63,49.61) | -0.18 (-0.19,-0.17) | 421.08 |
| Maldives | 42 (32,55) | 53.66 (40.67,71.29) | 181 (145,233) | 54.1 (41.34,71.72) | 0.23 (0.21,0.26) | 332.64 |
| Oman | 176 (134,227) | 28.99 (21.37,38.68) | 643 (504,829) | 34.43 (25.27,46.32) | -0.06 (-0.08,-0.05) | 265 |
| Saudi Arabia | 1686 (1284,2223) | 32.66 (24.17,44.12) | 6096 (4757,7921) | 36.92 (27.2,49.85) | 0.37 (0.36,0.38) | 261.66 |
| Islamic Republic of Iran | 8136 (6132,10787) | 37.34 (27.62,50.2) | 29418 (22665,38723) | 40.56 (30,54.38) | 0.12 (0.11,0.13) | 261.56 |
| Lebanon | 650 (494,855) | 33.33 (24.8,44.87) | 2244 (1665,3001) | 36.25 (26.97,48.31) | -0.05 (-0.07,-0.03) | 245.42 |
| Guam | 37 (28,47) | 53.53 (41.02,69.64) | 126 (97,164) | 59.14 (45.26,77.38) | -0.34 (-0.55,-0.13) | 245.3 |
| Guatemala | 1768 (1358,2343) | 55.48 (41.89,73.71) | 6076 (4623,8090) | 56.47 (42.71,75.76) | -0.29 (-0.37,-0.21) | 243.69 |
| Thailand | 17124 (13433,22527) | 52.67 (40.36,69.23) | 57918 (44292,77052) | 53.17 (40.48,70.77) | 0.03 (0.02,0.05) | 238.22 |
| Angola | 1311 (1002,1729) | 39.83 (30.05,53.22) | 4344 (3391,5660) | 41.84 (31.79,55.87) | 0.18 (0.17,0.2) | 231.38 |
| Timor-Leste | 134 (106,170) | 54.47 (41.71,72.09) | 439 (332,586) | 54.61 (41.79,72.26) | 0.25 (0.23,0.27) | 228.84 |
| Colombia | 9701 (7498,12735) | 57.96 (44.04,77.15) | 31451 (24378,41668) | 56.71 (43.35,75.87) | -0.48 (-0.53,-0.43) | 224.2 |
| Cameroon | 1413 (1076,1853) | 35.24 (26.59,47.05) | 4541 (3527,5945) | 39.23 (29.67,51.86) | 0.13 (0.12,0.14) | 221.35 |
| Nicaragua | 866 (669,1143) | 59.26 (44.92,78.79) | 2773 (2128,3642) | 58.13 (44.01,77.39) | 0 (-0.01,0.01) | 220.29 |
| Yemen | 1282 (964,1685) | 31.04 (22.91,41.47) | 4105 (3061,5427) | 32.9 (24.21,44.14) | 0.19 (0.18,0.2) | 220.14 |
| Singapore | 899 (704,1154) | 39.69 (30.61,52.17) | 2861 (2242,3726) | 33.07 (25.69,42.97) | -0.74 (-0.85,-0.64) | 218.41 |
| Belize | 55 (42,73) | 59.88 (45.68,79.39) | 175 (136,229) | 60.74 (45.89,80.02) | -0.75 (-0.88,-0.62) | 216.85 |
| Malaysia | 5041 (3955,6559) | 57.12 (43.61,75.3) | 15962 (12194,20998) | 58.73 (44.38,78.66) | 0.18 (0.16,0.19) | 216.64 |
| Togo | 413 (318,542) | 37.11 (28.07,49.68) | 1305 (1010,1717) | 37.53 (28.56,49.83) | 0.21 (0.18,0.23) | 215.56 |
| Algeria | 3727 (2696,5043) | 34.09 (24.78,46.4) | 11749 (8706,15697) | 35.37 (25.91,47.68) | 0.05 (0.02,0.07) | 215.2 |
| Ecuador | 2792 (2141,3638) | 54.84 (42,72.39) | 8791 (6715,11724) | 54.15 (41.24,71.88) | 0.06 (0.05,0.08) | 214.82 |
| Northern Mariana Islands | 9 (7,11) | 56.57 (43.15,75.34) | 28 (21,37) | 58.21 (44.24,76.14) | -0.75 (-0.89,-0.6) | 213.95 |
| Bangladesh | 19440 (14830,25449) | 45.54 (33.71,59.79) | 60982 (45614,80831) | 46.19 (34.26,61.31) | 0.04 (0.01,0.07) | 213.69 |
| Israel | 3551 (2614,4680) | 70.89 (53.65,92.18) | 11110 (8995,12779) | 91.98 (75.23,105.16) | 0.2 (0.15,0.24) | 212.88 |
| Mexico | 26349 (20418,35014) | 65.18 (50.08,86.54) | 82432 (63607,109361) | 66.4 (50.87,88.29) | 0.18 (0.17,0.2) | 212.84 |
| Equatorial Guinea | 66 (50,87) | 39.36 (29.69,53.03) | 205 (159,266) | 44.12 (33.56,58.64) | 0.42 (0.39,0.44) | 211.91 |
| Costa Rica | 1106 (845,1463) | 64.47 (48.81,86.04) | 3442 (2653,4536) | 62.53 (48,82.88) | 0.33 (0.3,0.36) | 211.37 |
| Peru | 6218 (4868,8165) | 54.04 (41.36,71.29) | 19333 (15032,25246) | 57.88 (44.6,76.58) | -0.37 (-0.4,-0.33) | 210.92 |
| Honduras | 1159 (896,1522) | 60.01 (46.1,79.46) | 3592 (2759,4716) | 58.92 (45.29,77.41) | 0.08 (0.07,0.09) | 209.93 |
| Niger | 846 (649,1123) | 34.61 (26.15,46.54) | 2620 (1999,3479) | 35.28 (26.73,47.18) | 0.09 (0.06,0.11) | 209.56 |
| Panama | 870 (667,1153) | 59.85 (45.7,79.52) | 2656 (2054,3532) | 59.92 (46.23,80.07) | 0.16 (0.13,0.19) | 205.21 |
| Republic of C么te d'Ivoire | 1300 (996,1708) | 37.12 (27.91,49.32) | 3940 (3081,5145) | 38.46 (29.28,51.82) | 0.01 (0,0.02) | 202.99 |
| Iraq | 2717 (2025,3570) | 36.58 (26.52,48.72) | 8151 (6210,10703) | 38.36 (28.13,51.24) | 0.16 (0.08,0.24) | 200 |
| China | 306585 (234243,404868) | 42.63 (32.4,56.46) | 916180 (707384,1201381) | 44.92 (34.96,59.42) | 0.12 (0.11,0.13) | 198.83 |
| Ghana | 2024 (1557,2654) | 36.14 (27.28,48.06) | 6048 (4667,7938) | 39.08 (29.56,51.95) | 0.06 (0.03,0.09) | 198.82 |
| Bolivarian Republic of Venezuela | 6130 (4764,7939) | 65.87 (50.36,86.05) | 18248 (13775,24049) | 61.32 (46.53,81.59) | 0.04 (0.02,0.07) | 197.69 |
| Tunisia | 1485 (1094,2018) | 32.93 (24.09,44.53) | 4414 (3251,5838) | 34.48 (25.02,45.65) | 0.17 (0.15,0.19) | 197.18 |
| Palestine | 252 (189,338) | 31.95 (23.55,42.92) | 749 (570,982) | 33.18 (24.4,44.66) | 0.52 (0.47,0.57) | 196.7 |
| Plurinational State of Bolivia | 1648 (1256,2170) | 55.11 (41.58,73.43) | 4880 (3734,6425) | 55.4 (42.03,74.02) | 0.07 (0.06,0.08) | 196.22 |
| Papua New Guinea | 751 (581,992) | 49.6 (37.99,65.71) | 2219 (1744,2890) | 49.67 (38.29,65.85) | -0.16 (-0.2,-0.13) | 195.41 |
| Republic of the Gambia | 116 (89,151) | 36.42 (27.6,48.08) | 342 (265,451) | 37.4 (28.57,50.28) | -1.21 (-1.47,-0.95) | 194.72 |
| Vanuatu | 32 (25,42) | 58.8 (44.26,77.86) | 93 (72,121) | 59.69 (45.68,78.54) | 0.29 (0.24,0.34) | 191.16 |
| Libya | 614 (465,806) | 35.68 (26.09,47.9) | 1777 (1361,2315) | 37.69 (27.8,50.68) | 0.13 (0.12,0.13) | 189.17 |
| Botswana | 201 (153,267) | 40.76 (30.74,54.2) | 581 (449,758) | 42.95 (32.63,57.14) | 0.03 (0,0.06) | 188.24 |
| India | 198419 (151420,263224) | 50.27 (37.44,66.79) | 570121 (430165,762853) | 51.45 (38.36,68.54) | -0.07 (-0.07,-0.06) | 187.33 |
| Bhutan | 94 (71,123) | 45.6 (33.8,60.36) | 269 (202,360) | 46.27 (34.64,61.83) | -0.12 (-0.14,-0.1) | 186.88 |
| Brazil | 58962 (45460,76802) | 68.76 (53.03,90.37) | 169133 (130789,219703) | 67.63 (52.17,88.49) | 0.16 (0.15,0.17) | 186.85 |
| Kenya | 3129 (2441,4088) | 41.04 (31.44,54.8) | 8973 (7020,11663) | 42.21 (32.33,56.29) | -0.9(-1.18,-0.62) | 186.8 |
| Philippines | 15964 (12390,20941) | 59.63 (45.31,79.4) | 45632 (35428,59598) | 60.3 (45.81,80.23) | 0.68 (0.61,0.75) | 185.84 |
| Republic of Korea | 14766 (11525,18924) | 49.39 (38.37,64.76) | 42194 (34817,52133) | 47.21 (39.34,57.49) | 0.21 (-0.05,0.46) | 185.75 |
| Cambodia | 2096 (1615,2727) | 53.26 (40.7,70.29) | 5973 (4647,7812) | 52.94 (40.15,69.72) | 0.05 (0.04,0.06) | 184.98 |
| Taiwan (Province of China) | 8121 (6340,10370) | 54.99 (43.02,71.18) | 22565 (17990,28132) | 53.21 (42.42,67.12) | 0.01 (-0.01,0.03) | 177.88 |
| Dominican Republic | 2106 (1609,2774) | 59.61 (45.77,78.47) | 5823 (4490,7736) | 58.97 (45.15,78.8) | 0.25 (0.23,0.26) | 176.54 |
| United Republic of Tanzania | 3448 (2650,4538) | 34.78 (26.8,46.2) | 9526 (7370,12404) | 39.1 (30.07,51.91) | 0.01 (0.01,0.02) | 176.31 |
| Ethiopia | 6365 (4844,8392) | 37.03 (28.2,49.29) | 17579 (13804,22707) | 42.11 (32.38,55.88) | 0.11 (0.11,0.12) | 176.18 |
| Sri Lanka | 5377 (4145,7100) | 54.08 (41.22,72.82) | 14672 (11154,19567) | 55.75 (42.39,73.31) | 0.4 (0.39,0.42) | 172.88 |
| Eritrea | 326 (253,428) | 33.94 (26.03,45.24) | 883 (684,1162) | 35.13 (26.99,47.06) | -0.04 (-0.05,-0.03) | 171.25 |
| Mauritius | 377 (291,492) | 56.64 (43.25,75.1) | 1022 (779,1353) | 56.83 (43.14,75.26) | 0.05 (0.03,0.07) | 171.04 |
| Congo | 379 (286,501) | 40.82 (30.79,54.27) | 1025 (797,1332) | 42.47 (32.26,56.38) | 0.09 (0.08,0.1) | 170.58 |
| Solomon Islands | 59 (45,78) | 51.19 (39.03,68.33) | 160 (124,212) | 51.92 (39.63,69.56) | 0.14 (0.13,0.16) | 170.53 |
| Egypt | 7316 (5543,9606) | 33.01 (24.14,44.24) | 19786 (14849,25927) | 36.92 (27.14,49.42) | -0.05 (-0.06,-0.04) | 170.44 |
| Syrian Arab Republic | 1567 (1191,2037) | 34.12 (25.03,45.49) | 4228 (3163,5674) | 35.28 (25.74,47.13) | -0.15 (-0.2,-0.09) | 169.76 |
| Brunei Darussalam | 48 (38,61) | 43.19 (33.56,56.47) | 130 (101,166) | 35.52 (27.3,45.61) | -0.24 (-0.29,-0.19) | 169 |
| Somalia | 726 (578,945) | 34.14 (26.01,45.43) | 1951 (1522,2560) | 35.55 (27.45,47.35) | -0.06 (-0.07,-0.05) | 168.84 |
| Comoros | 63 (49,82) | 35.58 (26.93,47.29) | 170 (132,226) | 36.7 (28.01,49.08) | -0.05 (-0.07,-0.03) | 168.44 |
| Austria | 7033 (6199,8047) | 57.77 (51.28,65.36) | 18808 (17782,19775) | 105.58 (100.49,110.74) | 2.21 (2.07,2.34) | 167.41 |
| Saint Lucia | 54 (40,72) | 62.01 (47.82,82.34) | 143 (109,187) | 59.78 (45.19,77.74) | 0.1 (0.09,0.11) | 167 |
| Socialist Republic of Viet Nam | 20315 (15605,26791) | 53.94 (41.15,71.13) | 53983 (41974,71154) | 57.26 (43.75,76.67) | 0.03 (0.02,0.04) | 165.73 |
| Benin | 657 (505,859) | 35.02 (26.78,45.86) | 1743 (1366,2273) | 36.28 (27.75,48.29) | 0.06 (0.04,0.08) | 165.23 |
| Nepal | 3700 (2805,4891) | 45.84 (34.18,61) | 9675 (7232,12862) | 44.58 (33.43,59.27) | 0 (-0.03,0.03) | 161.46 |
| Indonesia | 55881 (43453,72892) | 64.43 (49.08,85.9) | 145423 (112648,190123) | 66.36 (50.84,87.95) | -0.67 (-0.72,-0.61) | 160.23 |
| Zambia | 903 (702,1185) | 34.96 (26.67,46.91) | 2320 (1828,3002) | 35.81 (27.43,47.89) | 0.08 (0.07,0.09) | 156.98 |
| Commonwealth of the Bahamas | 92 (70,121) | 61.88 (46.8,81.61) | 234 (181,309) | 59.29 (45.09,78.28) | 0.16 (0.08,0.25) | 154.68 |
| Paraguay | 1475 (1135,1909) | 67.17 (52.06,88.01) | 3697 (2878,4841) | 63.72 (49.18,84.12) | 0.07 (0.05,0.1) | 150.74 |
| Morocco | 4690 (3492,6273) | 35.71 (26,48) | 11738 (8720,15856) | 36.59 (26.88,49.55) | -0.02 (-0.03,-0.01) | 150.25 |
| Republic of Palau | 5 (4,6) | 53.39 (40.57,71.18) | 12 (9,16) | 56.92 (43.46,75.08) | -0.15 (-0.32,0.02) | 149.99 |
| Senegal | 1107 (845,1455) | 37.2 (27.94,49.35) | 2744 (2111,3606) | 37.78 (28.58,50.72) | 0.06 (0.04,0.09) | 147.88 |
| Cook Islands | 6 (5,8) | 53.77 (40.76,71.21) | 15 (12,20) | 59.44 (45.14,78.03) | 0.14 (0.13,0.15) | 147.3 |
| Suriname | 146 (114,192) | 59.89 (45.74,79.67) | 361 (277,477) | 58.05 (44.73,77.23) | 0.83 (0.7,0.96) | 146.82 |
| Turkey | 10042 (7802,12979) | 32.02 (24.36,41.7) | 24774 (21786,28278) | 27.35 (24.06,31.31) | -0.78 (-0.89,-0.67) | 146.69 |
| Fiji | 176 (136,229) | 55.98 (42.17,74.75) | 428 (326,563) | 61.36 (46.49,80.16) | 0.12 (0.11,0.14) | 143.7 |
| Uzbekistan | 4414 (3384,5822) | 39.64 (29.94,52.67) | 10746 (8148,14124) | 41.51 (30.94,54.26) | -0.24 (-0.26,-0.23) | 143.43 |
| American Samoa | 11 (9,15) | 56.33 (42.71,73.79) | 27 (21,36) | 59.56 (45.61,79.26) | 0.15 (0.12,0.18) | 138.48 |
| Rwanda | 949 (737,1237) | 37.59 (28.8,49.6) | 2248 (1751,2936) | 37.96 (29.04,50) | -0.11 (-0.14,-0.08) | 137.03 |
| Trinidad and Tobago | 524 (395,698) | 63.52 (49.01,84.29) | 1240 (932,1641) | 63.93 (48.81,84.75) | 0.28 (0.27,0.29) | 136.79 |
| Uganda | 2290 (1765,2999) | 38.45 (29.55,51.14) | 5416 (4239,7160) | 38.93 (29.92,52.22) | 0.29 (0.22,0.36) | 136.46 |
| Chile | 4534 (3449,6005) | 46.21 (35.02,61.54) | 10713 (8108,14239) | 41.63 (31.85,55.05) | -0.02 (-0.02,-0.01) | 136.28 |
| Sudan | 2800 (2050,3759) | 33.7 (24.56,45.03) | 6614 (4970,8739) | 37.78 (27.49,50.99) | -0.12 (-0.14,-0.11) | 136.25 |
| Mali | 1193 (910,1586) | 33.84 (25.66,45.78) | 2815 (2156,3713) | 35.17 (26.68,47.05) | 0.2 (0.16,0.24) | 136.06 |
| Turkmenistan | 794 (597,1039) | 43.24 (32.26,56.89) | 1872 (1439,2433) | 47.04 (35.47,60.99) | -0.04 (-0.08,0) | 135.88 |
| Nigeria | 15157 (11648,20152) | 37.27 (28.27,50.01) | 35723 (28008,46500) | 42.52 (32.42,56.76) | -0.07 (-0.13,-0.01) | 135.69 |
| Czech Republic | 7616 (5591,10112) | 54.7 (40.9,71.71) | 17905 (14041,20271) | 81.97 (65.98,91.63) | -0.81 (-0.91,-0.72) | 135.12 |
| Madagascar | 1630 (1260,2145) | 35.21 (27.02,47.17) | 3804 (2959,5017) | 37.01 (28.34,48.94) | 0.35 (0.31,0.4) | 133.32 |
| Lao People's Democratic Republic | 1000 (757,1324) | 55.76 (42.56,74.12) | 2333 (1816,3027) | 54.97 (41.92,72.84) | 0.19 (0.13,0.24) | 133.28 |
| Mauritania | 330 (249,441) | 35.53 (27.05,47.76) | 767 (589,1011) | 37.53 (28.39,50.3) | -0.09 (-0.38,0.2) | 132.1 |
| Democratic Republic of the Congo | 5372 (4076,7087) | 40.23 (30.46,53.18) | 12383 (9617,16381) | 38.47 (28.94,51.58) | 0.11 (0.1,0.12) | 130.52 |
| United States Virgin Islands | 50 (38,66) | 61.81 (46.68,82.23) | 115 (83,157) | 62.03 (47.02,82.25) | 0.1 (0.08,0.12) | 130.05 |
| Mongolia | 450 (338,593) | 44.09 (33.03,57.99) | 1034 (782,1341) | 45.64 (34.27,60.09) | -0.1 (-0.15,-0.05) | 129.79 |
| Burkina Faso | 1373 (1046,1825) | 35.03 (26.64,46.61) | 3131 (2428,4105) | 36.46 (27.89,48.51) | -0.05 (-0.09,-0.01) | 128.04 |
| Haiti | 1745 (1328,2272) | 59.81 (45.73,79.63) | 3950 (3038,5216) | 59.42 (44.98,79.53) | 0.11 (0.1,0.12) | 126.32 |
| Australia | 14443 (13099,15906) | 73.34 (66.69,80.69) | 32531 (24219,42746) | 74.05 (56.85,96.39) | 0.09(0.04,0.15) | 125.23 |
| Andorra | 41 (30,54) | 69.12 (51.57,91.7) | 91 (68,119) | 60.36 (45.23,78.41) | -0.58 (-0.64,-0.52) | 123.68 |
| South Africa | 9549 (7351,12620) | 49.4 (37.52,66.24) | 21323 (16354,28253) | 48.94 (37.27,64.92) | 0.24 (0.22,0.26) | 123.31 |
| Azerbaijan | 2079 (1577,2727) | 43.33 (32.63,56.59) | 4617 (3523,6113) | 44.9 (33.78,58.97) | 0.14 (0.12,0.16) | 122.14 |
| Namibia | 233 (174,308) | 40.47 (30.73,53.77) | 515 (390,681) | 40.81 (31.04,55.47) | -0.17 (-0.2,-0.14) | 121.31 |
| Myanmar | 11700 (8930,15322) | 57.5 (43.4,76.58) | 25401 (19548,33339) | 55.75 (42.05,74.46) | 0.1 (0.09,0.12) | 117.1 |
| Marshall Islands | 7 (6,10) | 51.39 (39.1,67.49) | 16 (12,21) | 52.48 (40.09,69.43) | 0.13 (0.12,0.14) | 116.79 |
| United States of America | 245056 (180693,325021) | 75.22 (57.39,99.34) | 528208 (487191,574743) | 89.18 (82.53,96.66) | -0.44 (-0.49,-0.4) | 115.55 |
| Tajikistan | 1084 (832,1420) | 41.09 (31.09,53.75) | 2333 (1786,3072) | 40.85 (30.53,53.3) | 0.04 (0.03,0.06) | 115.17 |
| Bermuda | 38 (29,51) | 62.17 (47.44,82.32) | 82 (62,110) | 60.11 (45.87,79.58) | 0.12 (0.11,0.13) | 114.73 |
| Democratic People's Republic of Korea | 7060 (5522,9165) | 49.69 (38.11,65.61) | 15152 (11772,20144) | 48.09 (37.23,63.29) | 1.5 (1.07,1.93) | 114.62 |
| Malawi | 1267 (975,1639) | 36.49 (27.89,48.03) | 2719 (2109,3516) | 39.29 (30.1,52.01) | -0.08 (-0.11,-0.05) | 114.62 |
| Iceland | 175 (129,230) | 60.61 (45.01,78.8) | 375 (296,464) | 65.91 (52.74,81.71) | 0 (-0.02,0.02) | 114.46 |
| Burundi | 791 (607,1035) | 36.9 (28.28,48.88) | 1690 (1320,2214) | 37.32 (28.58,49.92) | 0.12 (0.1,0.13) | 113.79 |
| Poland | 23417 (17441,30849) | 53.07 (40,69.57) | 50025 (36265,66677) | 69.46 (51.87,90.62) | 0.19 (0.16,0.22) | 113.63 |
| El Salvador | 1707 (1311,2234) | 58.97 (44.52,78.27) | 3647 (2778,4768) | 58.15 (44.23,76.71) | 0 (-0.02,0.02) | 113.6 |
| Pakistan | 27497 (20887,36777) | 53.26 (39.62,71.06) | 57874 (44289,75861) | 53.85 (40.3,71.6) | 0.06 (0.05,0.08) | 110.47 |
| Albania | 995 (742,1295) | 47.94 (35.37,62.61) | 2070 (1507,2764) | 46.19 (34.59,60.4) | -0.1 (-0.13,-0.07) | 108.11 |
| Saint Vincent and the Grenadines | 40 (30,54) | 57.27 (43.51,76.79) | 84 (64,112) | 59.11 (44.99,78.29) | 0 (-0.02,0.02) | 107.49 |
| Chad | 905 (680,1215) | 34.2 (26.02,45.78) | 1862 (1437,2449) | 35.4 (26.67,47.34) | -0.67 (-0.78,-0.55) | 105.84 |
| New Zealand | 2848 (2164,3675) | 71.08 (54.87,91.02) | 5817 (4707,7311) | 69.78 (57.18,86.67) | -0.1 (-0.11,-0.1) | 104.26 |
| Malta | 251 (181,332) | 57.92 (42.47,76.67) | 512 (438,589) | 51.15 (44.57,58) | -0.01 (-0.03,0) | 104.24 |
| Kingdom of Eswatini | 107 (82,140) | 41.67 (31.37,55.9) | 217 (167,287) | 43.34 (32.97,57.62) | 0.22 (0.21,0.24) | 103.86 |
| Kiribati | 17 (13,23) | 55.34 (41.81,73.56) | 35 (27,46) | 55.96 (42.42,75.07) | 0.03 (0,0.06) | 102.14 |
| Mozambique | 1958 (1514,2574) | 35.96 (27.52,48.05) | 3914 (3015,5091) | 38.13 (28.99,50.32) | -0.53 (-0.59,-0.47) | 99.9 |
| Seychelles | 32 (24,43) | 57.65 (43.89,76.07) | 64 (50,83) | 57.24 (43.64,74.83) | -0.05 (-0.07,-0.04) | 98.45 |
| Puerto Rico | 2259 (1719,3011) | 62.11 (47.9,82.27) | 4460 (3243,5952) | 61.38 (45.95,80.92) | 0.01 (-0.01,0.02) | 97.46 |
| Greenland | 30 (23,39) | 96.28 (72.66,125.85) | 60 (44,79) | 86.59 (65.04,114.49) | -0.03 (-0.18,0.12) | 95.82 |
| Republic of Cabo Verde | 83 (62,114) | 36.05 (27.37,48.38) | 163 (127,214) | 37.51 (28.45,50.47) | 0.34 (0.28,0.39) | 95.34 |
| Sweden | 13182 (9876,17727) | 85.18 (65.88,110.36) | 25710 (18631,33708) | 123.84 (92.51,159.67) | 0.09 (-0.28,0.47) | 95.03 |
| Cyprus | 477 (347,625) | 55.82 (42,72.7) | 928 (720,1218) | 42.95 (34.02,55.26) | -0.1 (-0.12,-0.08) | 94.81 |
| Central African Republic | 380 (287,504) | 39.89 (30.12,53.39) | 737 (569,976) | 39.69 (30.18,52.71) | 0.33 (0.26,0.4) | 94.05 |
| Antigua and Barbuda | 31 (23,42) | 57.22 (43.72,75.88) | 60 (46,80) | 57.46 (43.94,75.87) | 0 (-0.01,0.01) | 92.83 |
| Gabon | 214 (162,288) | 40.53 (30.35,54.15) | 407 (313,532) | 42.53 (32.55,56.71) | -1.16 (-1.25,-1.06) | 90.22 |
| Guinea-Bissau | 124 (93,164) | 35.6 (26.79,46.54) | 233 (180,308) | 36.54 (27.84,49.11) | 0.32 (0.29,0.35) | 88.32 |
| Cuba | 6120 (4594,8183) | 59.93 (45.39,79.13) | 11429 (8751,15178) | 58.1 (44.65,76.07) | 0.86 (0.6,1.12) | 86.76 |
| Liberia | 392 (295,518) | 36.68 (27.93,49.2) | 726 (570,941) | 37.02 (27.98,49.14) | 0.3 (0.29,0.31) | 85.07 |
| Sao Tome and Principe | 22 (17,30) | 36.16 (27.55,48.63) | 41 (32,53) | 38.74 (29.34,52.48) | 0.21 (0.18,0.24) | 82.3 |
| Slovakia | 3990 (3313,4629) | 65.82 (55.05,75.92) | 7231 (5730,8496) | 73.15 (58.9,85.48) | 0.25 (0.17,0.33) | 81.25 |
| Luxembourg | 380 (310,475) | 67.74 (56.09,83.07) | 681 (580,770) | 65.48 (56.11,73.74) | 0.15 (0.13,0.17) | 79.22 |
| Tuvalu | 3 (2,4) | 52.18 (40.21,69.23) | 5 (4,7) | 55.34 (42.18,72.65) | 0.15 (0.12,0.18) | 78.96 |
| Sierra Leone | 785 (593,1049) | 40.49 (31.02,53.67) | 1403 (1091,1862) | 39.73 (30.33,53.31) | -0.11 (-0.13,-0.08) | 78.73 |
| Jamaica | 1041 (786,1388) | 57.72 (44.41,76.49) | 1851 (1423,2396) | 59.1 (44.9,77.87) | -1.06 (-1.21,-0.91) | 77.77 |
| Barbados | 178 (128,242) | 59.42 (45.08,79.16) | 316 (237,425) | 60.95 (46.79,81.94) | 0.06 (0.01,0.12) | 77.69 |
| Samoa | 43 (34,57) | 56.53 (43.2,75.2) | 77 (60,102) | 57.07 (43.2,75.97) | -0.56 (-0.6,-0.51) | 77.66 |
| Armenia | 1140 (872,1502) | 43.01 (32.14,56.2) | 2013 (1479,2662) | 45.65 (34.23,60.63) | 0.21 (0.19,0.23) | 76.57 |
| The former Yugoslav Republic of Macedonia | 914 (683,1207) | 48.59 (36.06,64.08) | 1603 (1158,2145) | 45.77 (33.88,60.43) | 0.04 (0.02,0.05) | 75.42 |
| Guinea | 1068 (809,1412) | 34.48 (26.09,45.94) | 1867 (1435,2453) | 35.62 (26.88,47.94) | 0 (-0.04,0.03) | 74.83 |
| Canada | 33635 (25923,42484) | 100.87 (78.41,126.21) | 58043 (42013,77003) | 80.6 (59.82,106.65) | -0.03 (-0.05,-0.02) | 72.56 |
| Zimbabwe | 1472 (1118,1937) | 40.11 (30.15,53.61) | 2534 (1926,3337) | 41.28 (31.18,55.1) | 0.09 (0.08,0.11) | 72.12 |
| Saint Kitts and Nevis | 23 (16,31) | 59.61 (45.54,78.91) | 39 (29,51) | 58.19 (44.27,77.42) | -0.07 (-0.09,-0.04) | 70.67 |
| Republic of San Marino | 24 (18,32) | 67.3 (50.42,87.53) | 41 (30,54) | 58.5 (43.3,76.78) | 0.36 (0.35,0.38) | 70.59 |
| Guyana | 219 (168,288) | 61.15 (46.34,81.74) | 364 (277,481) | 59.63 (45.02,79.68) | 0.13 (0.09,0.17) | 66.27 |
| Tonga | 28 (21,37) | 54.9 (41.6,73.39) | 46 (35,60) | 58.48 (44.53,76.74) | 0.13(0.12,0.14) | 65.31 |
| Slovenia | 1229 (920,1632) | 50.16 (37.53,66.24) | 2024 (1731,2316) | 48.42 (42.05,54.65) | -0.09 (-0.14,-0.04) | 64.79 |
| South Sudan | 828 (632,1090) | 35.02 (26.77,46.61) | 1351 (1062,1777) | 37.43 (28.5,49.65) | -0.01 (-0.14,0.12) | 63.08 |
| Kyrgyzstan | 1203 (909,1595) | 41.46 (31,54.33) | 1957 (1491,2572) | 41.13 (31.43,53.83) | 0.03 (0.01,0.05) | 62.73 |
| Croatia | 1919 (1584,2351) | 30.99 (25.33,38.17) | 3045 (2682,3466) | 34.78 (30.93,39.18) | -0.12 (-0.13,-0.11) | 58.7 |
| Ireland | 2713 (1936,3616) | 64.43 (47.57,84.46) | 4292 (3141,5685) | 54.42 (40.84,71.55) | 0.09 (0.08,0.1) | 58.2 |
| Bosnia and Herzegovina | 1861 (1414,2479) | 45.79 (34.25,60.36) | 2895 (2119,3855) | 46.03 (34.49,60.17) | 0.05 (0.03,0.08) | 55.59 |
| Portugal | 9401 (6872,12569) | 65.52 (49.07,86.59) | 14593 (11898,18076) | 59.99 (49.76,72.76) | 0.07 (0.06,0.09) | 55.22 |
| Spain | 41675 (31727,53592) | 74.66 (57.65,95) | 64456 (56846,73040) | 69.9 (62.22,78.58) | 0.1 (0.08,0.11) | 54.66 |
| Montenegro | 313 (235,408) | 50.48 (37.49,65.94) | 484 (353,637) | 47.55 (35.29,62.08) | 0.11 (0.09,0.12) | 54.63 |
| Netherlands | 14708 (13196,16558) | 72.21 (64.26,80.62) | 22455 (18709,27075) | 64.02 (54.48,75.58) | 0.17 (0.15,0.19) | 52.67 |
| Japan | 70805 (54525,93790) | 42.03 (32.59,55.25) | 107285 (77975,144807) | 31.86 (24.84,41.91) | 1.25 (0.97,1.53) | 51.52 |
| Grenada | 43 (32,58) | 58.26 (44.46,77.85) | 65 (49,86) | 58.42 (44.63,77.91) | 0.17 (0.1,0.24) | 49.64 |
| Kazakhstan | 5935 (4511,7796) | 48.19 (36.41,63.55) | 8829 (6626,11519) | 48.85 (37.09,63.65) | 0.1 (0.07,0.13) | 48.75 |
| Switzerland | 4309 (3281,5690) | 40.72 (31.61,52.93) | 6346 (5485,7579) | 36.45 (31.83,42.53) | 0.1 (0.09,0.11) | 47.27 |
| Federated States of Micronesia | 24 (18,31) | 54.03 (41.13,71.19) | 35 (27,46) | 53.81 (41.12,71.15) | 0.36 (0.32,0.4) | 47.09 |
| Republic of Moldova | 1925 (1459,2517) | 43.01 (32.7,55.93) | 2822 (2114,3649) | 47.44 (36.08,60.91) | -0.24 (-0.32,-0.16) | 46.58 |
| Russian Federation | 86356 (65246,113303) | 47.79 (36.77,62.21) | 125499 (94653,165367) | 52.32 (40.22,68.2) | 0 (-0.01,0.01) | 45.33 |
| Italy | 68691 (50618,91262) | 76.05 (57.71,99.91) | 99307 (72177,134792) | 70.54 (52.49,92.64) | 0.13 (0.12,0.15) | 44.57 |
| Afghanistan | 1906 (1415,2571) | 31.95 (23.7,42.95) | 2735 (2056,3602) | 33.33 (24.48,44.48) | 0.13 (0.11,0.15) | 43.53 |
| Dominica | 35 (26,47) | 58.83 (45.26,78.42) | 49 (37,65) | 59.66 (45.28,78.68) | -0.39 (-0.57,-0.21) | 41.21 |
| Denmark | 5649 (4210,7572) | 68.96 (52.48,89.77) | 7912 (5907,10323) | 70.03 (54.45,89.03) | -0.13 (-0.15,-0.12) | 40.06 |
| Greece | 8833 (7049,11378) | 57.29 (45.98,72.87) | 12367 (9012,16893) | 54.67 (41,71.66) | -0.06 (-0.08,-0.03) | 40.01 |
| Germany | 101466 (75243,135577) | 79.75 (60.18,104.73) | 140086 (122558,158105) | 81.28 (72.34,89.74) | 0.15 (0.15,0.16) | 38.06 |
| France | 57571 (42070,77071) | 68.85 (51.4,90.39) | 78372 (57303,105675) | 59.15 (44.34,78.68) | 0.32 (0.29,0.35) | 36.13 |
| Republic of Nauru | 2 (2,3) | 57.79 (43.81,77.26) | 3 (2,4) | 60.59 (45.99,80.04) | -0.04 (-0.06,-0.02) | 35.55 |
| United Kingdom of Great Britain and Northern Ireland | 48564 (36703,63566) | 53.06 (41.35,68.26) | 65549 (51519,83607) | 52.25 (41.8,65.6) | 0.54 (0.48,0.61) | 34.97 |
| Belarus | 5642 (4239,7454) | 43.56 (32.82,56.35) | 7544 (5606,9888) | 47.24 (35.87,61.53) | 0.1 (0.08,0.12) | 33.72 |
| Estonia | 871 (648,1141) | 42.66 (32.14,54.94) | 1149 (859,1498) | 46.91 (35.52,60.22) | 0.39 (0.38,0.41) | 31.89 |
| Lithuania | 1975 (1485,2581) | 43.97 (33.46,57.27) | 2548 (1918,3354) | 48.23 (36.45,62.39) | 0.04 (0.03,0.05) | 29.07 |
| Lesotho | 300 (227,402) | 38.22 (28.75,50.95) | 387 (293,514) | 39.92 (30.15,53.63) | 0.86 (0.67,1.05) | 28.9 |
| Norway | 4758 (3431,6405) | 69.29 (52.99,90.88) | 6082 (4530,8012) | 62.72 (47.43,82.17) | -0.03 (-0.11,0.06) | 27.81 |
| Uruguay | 1675 (1238,2253) | 42.52 (32,56.67) | 2129 (1563,2898) | 38.49 (28.98,51.8) | 0.01 (-0.02,0.03) | 27.1 |
| Tokelau | 1 (1,1) | 51.88 (39.68,69.51) | 1 (1,1) | 56.13 (42.64,73.71) | 0 (-0.02,0.02) | 26.05 |
| Finland | 6773 (4959,8593) | 94.77 (70.63,119.71) | 8476 (6391,11009) | 69.89 (55.04,86.68) | 0.51 (0.45,0.57) | 25.15 |
| Argentina | 13363 (10066,17929) | 41.84 (31.73,55.61) | 16008 (13581,19205) | 28.5 (24.36,34.01) | -1.31 (-1.62,-0.99) | 19.79 |
| Principality of Monaco | 47 (34,63) | 66.75 (49.53,87.83) | 55 (40,74) | 58.82 (44.19,76.65) | 0.03 (0.01,0.05) | 18.23 |
| Latvia | 1446 (1077,1905) | 40.6 (30.59,52.86) | 1701 (1429,1990) | 47.4 (40.34,54.56) | -0.06 (-0.07,-0.05) | 17.6 |
| Belgium | 9867 (7416,13339) | 62.56 (47.73,83.44) | 11602 (8964,15260) | 53.01 (41.85,67.78) | 0.25 (0.21,0.29) | 17.58 |
| Ukraine | 31985 (24201,42017) | 44.68 (34.28,57.82) | 35530 (26656,46895) | 46.19 (35.34,60.16) | -0.27 (-0.39,-0.14) | 11.08 |
| Serbia | 5307 (3932,7011) | 45.39 (33.82,59.77) | 5688 (4597,7029) | 34.05 (28.15,41.75) | -1 (-1.25,-0.74) | 7.19 |
| Hungary | 8383 (6153,11236) | 55.95 (41.54,73.85) | 8870 (6408,11720) | 46.18 (34.66,60.55) | -0.06 (-0.08,-0.04) | 5.82 |
| Bulgaria | 6135 (4417,8233) | 47.44 (34.84,62.38) | 6484 (4590,8604) | 45.66 (33.96,60.04) | -0.73 (-0.8,-0.67) | 5.7 |
| Republic of Niue | 1 (1,2) | 56.96 (43.31,75.64) | 1 (1,2) | 60.47 (46.2,79.8) | 0.15 (0.12,0.19) | -1.84 |
| Georgia | 2950 (2187,3910) | 47.31 (35.38,61.93) | 2830 (2114,3690) | 47.21 (35.92,61.51) | -0.64 (-0.71,-0.57) | -4.09 |
| Romania | 13355 (9778,17808) | 47.05 (34.77,61.61) | 12515 (10602,15109) | 33.74 (29.07,40.1) | 0.33 (0.25,0.4) | -6.29 |
|  | Number(95%UI) | Rate(95%UI) | Number(95%UI) | Rate(95%UI) | EAPC_95%CI |  |

AF/AFL, Atrial fibrillation (AF)/atrial flutter (AFL); CI, confidence interval; EAPC, estimated annual percentage change; UI, uncertainty interval.

Supplementary Table 5 Prevalence of AF/AFL Between 1990 and 2021 in 204 countries

| **Location** | **Incidence in 1990** | | **Incidence in 2021** | | **1990-2021 EAPC** | **1990-2021 change** |
| --- | --- | --- | --- | --- | --- | --- |
|  | **Number(95%UI)** | **Rate(95%UI)** | **Number(95%UI)** | **Rate(95%UI)** | **EAPC_95%CI** |  |
| Afghanistan | 17747 (13622,23160) | 312.82 (240.08,412.25) | 25639 (19701,33184) | 338.76 (257.55,442.8) | 0.26 (0.23,0.29) | 44.47 |
| Albania | 10877 (8359,13976) | 576.46 (437.91,747.53) | 26254 (20002,34169) | 582.74 (445.93,753.86) | 0.07 (0.04,0.1) | 141.36 |
| Algeria | 34903 (26341,46477) | 350.33 (265.16,464.73) | 116519 (89354,153163) | 376.73 (287.03,499.12) | 0.17 (0.14,0.19) | 233.84 |
| American Samoa | 113 (89,149) | 620 (485.43,805.63) | 289 (229,380) | 672.65 (528.47,875.08) | 0.21 (0.18,0.25) | 154.79 |
| Andorra | 462 (354,595) | 835.12 (645.86,1082.98) | 1172 (905,1510) | 743.99 (574.87,956.34) | -0.53 (-0.58,-0.47) | 153.5 |
| Angola | 12292 (9545,16176) | 419.99 (324.84,550.58) | 41399 (32326,54332) | 452.08 (354.11,588.63) | 0.27 (0.26,0.28) | 236.81 |
| Antigua and Barbuda | 361 (277,468) | 640.52 (500.21,831.03) | 664 (518,869) | 657.2 (512.46,858.3) | 0.07 (0.05,0.08) | 84.05 |
| Argentina | 144409 (112881,189972) | 462.57 (362.38,606.35) | 194835 (169094,228621) | 340.72 (296.16,399.78) | -1.03 (-1.32,-0.74) | 34.92 |
| Armenia | 12536 (9693,16435) | 507.82 (391.11,664.63) | 24452 (18788,32257) | 556.65 (424.75,729.78) | 0.33 (0.31,0.35) | 95.06 |
| Australia | 176034 (164773,187556) | 900.54 (846.03,956.65) | 435826 (338605,563002) | 928.31 (725.71,1201.33) | 0.19 (0.13,0.26) | 147.58 |
| Austria | 80258 (71857,89871) | 643.17 (578.86,714.48) | 241015 (230229,251784) | 1217.22 (1164.84,1272.17) | 2.33 (2.19,2.48) | 200.3 |
| Azerbaijan | 22683 (17563,29382) | 501.7 (385.38,655.28) | 48767 (38083,64570) | 540.88 (415.38,701.51) | 0.29 (0.27,0.31) | 114.99 |
| Bahrain | 462 (356,611) | 361.4 (274.6,473.49) | 2667 (2055,3468) | 386.71 (293.82,505.55) | -0.06 (-0.08,-0.05) | 476.93 |
| Bangladesh | 190657 (146465,248663) | 472.02 (362.32,621.61) | 623060 (480696,812961) | 494.7 (380.67,643.93) | 0.18 (0.16,0.21) | 226.8 |
| Barbados | 2085 (1592,2746) | 666.99 (518.95,866.63) | 3693 (2829,4833) | 701.8 (540.68,913.61) | 0.17 (0.11,0.23) | 77.11 |
| Belarus | 71527 (55047,92982) | 556.58 (429.87,719.16) | 101036 (78621,132534) | 620.57 (486.13,809.95) | 0.17 (0.15,0.19) | 41.26 |
| Belgium | 112962 (86002,147149) | 704.67 (544.2,918.24) | 163236 (131345,207711) | 675.51 (553.96,847.21) | 0.34 (0.31,0.38) | 44.5 |
| Belize | 607 (476,781) | 668.4 (523.01,861.13) | 1881 (1485,2467) | 693.37 (538.53,897.5) | -0.22 (-0.3,-0.15) | 209.8 |
| Benin | 6795 (5253,8934) | 377.41 (290.58,493.99) | 17562 (13735,23147) | 396.48 (308.75,520.98) | 0.14 (0.12,0.16) | 158.44 |
| Bermuda | 423 (328,552) | 705.31 (550.87,914.8) | 1007 (782,1312) | 700.19 (547.46,909.57) | 0.16 (0.15,0.17) | 138.14 |
| Bhutan | 864 (668,1134) | 467.22 (356.2,612.99) | 2770 (2170,3630) | 492.91 (384.1,647.49) | -0.04 (-0.06,-0.03) | 220.49 |
| Bolivarian Republic of Venezuela | 65247 (51221,84287) | 743.12 (578.4,960.3) | 202304 (158201,263377) | 700.83 (544.82,906.43) | 0.18 (0.15,0.2) | 210.06 |
| Bosnia and Herzegovina | 20194 (15626,26610) | 551.75 (424.34,722.2) | 36920 (28206,47883) | 574.34 (442.67,741.06) | 0.18 (0.16,0.19) | 82.83 |
| Botswana | 1971 (1525,2589) | 435.97 (337.45,570.77) | 5721 (4459,7586) | 467.19 (359.37,610.96) | 0.17 (0.14,0.2) | 190.2 |
| Brazil | 644967 (507649,839852) | 802.83 (631.35,1041.09) | 1961680 (1546310,2544332) | 795.32 (626.23,1025.71) | 0.21 (0.21,0.22) | 204.15 |
| Brunei Darussalam | 525 (412,677) | 537.28 (419.06,696.7) | 1479 (1155,1910) | 452.03 (354.84,582.96) | -0.07 (-0.09,-0.06) | 181.78 |
| Bulgaria | 70573 (53726,92308) | 580.26 (444.98,749.7) | 86405 (64937,113573) | 564.76 (430.67,735.55) | -0.66 (-0.71,-0.61) | 22.43 |
| Burkina Faso | 13882 (10757,18200) | 377.76 (293.77,494.41) | 31626 (24731,41910) | 397.21 (311.38,519) | -0.01 (-0.05,0.03) | 127.82 |
| Burundi | 8282 (6487,10976) | 407.99 (320.53,536.99) | 17035 (13278,22301) | 418.87 (326.88,552.33) | 0.14 (0.13,0.16) | 105.7 |
| Cambodia | 20822 (16350,27382) | 562.64 (441.69,725.52) | 61483 (48621,81182) | 578.56 (457.17,751.32) | 0.1 (0.09,0.11) | 195.28 |
| Cameroon | 14149 (10972,18719) | 381.73 (293.64,497.91) | 45126 (35031,58573) | 432.9 (335.41,563.68) | 0.19 (0.18,0.2) | 218.93 |
| Canada | 383916 (305778,474738) | 1163.51 (929.48,1432.68) | 731819 (554975,954038) | 958.83 (733.17,1244.27) | 0.08 (0.06,0.09) | 90.62 |
| Central African Republic | 3545 (2742,4677) | 416.27 (320.82,545.51) | 6761 (5202,8922) | 416.26 (325.14,546.14) | 0.38 (0.31,0.45) | 90.73 |
| Chad | 9335 (7171,12424) | 366 (283.48,480.61) | 18475 (14434,24478) | 383.73 (298.18,504.47) | -0.53 (-0.65,-0.42) | 97.92 |
| Chile | 48779 (37923,63534) | 517.99 (401.73,671.12) | 127594 (98729,165412) | 489.14 (379.61,632.16) | -0.01 (-0.01,0) | 161.58 |
| China | 3195309 (2518983,4168290) | 457.72 (358.93,594.96) | 10775721 (8531627,14014036) | 524 (418.15,681.23) | 0.16 (0.16,0.17) | 237.24 |
| Colombia | 101960 (80303,132427) | 648.41 (506.48,845.13) | 361800 (282565,475575) | 650.75 (507.97,853.91) | -0.32 (-0.37,-0.27) | 254.84 |
| Commonwealth of the Bahamas | 978 (767,1269) | 691.07 (536.42,899.86) | 2533 (1987,3335) | 674.57 (529.29,883.91) | 0.48 (0.38,0.58) | 158.99 |
| Comoros | 655 (509,868) | 395.77 (309.16,515.75) | 1811 (1415,2366) | 415.57 (326.17,548.36) | 0.04 (0.02,0.05) | 176.36 |
| Congo | 3685 (2852,4857) | 433.69 (337.43,567.64) | 9845 (7730,12833) | 462.37 (360.8,600.06) | 0.15 (0.14,0.16) | 167.17 |
| Cook Islands | 65 (51,87) | 595.69 (462.18,769.16) | 178 (138,232) | 683.51 (533.18,886.86) | 0.22 (0.2,0.23) | 171.78 |
| Costa Rica | 12283 (9603,15941) | 740.27 (578.22,963.56) | 39959 (31002,51910) | 727.25 (561.47,946.4) | 0.44 (0.4,0.48) | 225.32 |
| Croatia | 22653 (19259,27369) | 388.27 (328.33,464.05) | 39620 (35576,44450) | 420.02 (381.35,470.13) | -0.07 (-0.08,-0.07) | 74.9 |
| Cuba | 69471 (53452,90480) | 684.27 (530.29,890.3) | 136060 (106604,175458) | 671.42 (527.93,869.79) | 0.16 (0.15,0.17) | 95.85 |
| Cyprus | 5114 (3908,6514) | 652.09 (507.78,834.3) | 10427 (8434,13170) | 487.17 (395.53,615.62) | 0.69 (0.45,0.93) | 103.88 |
| Czech Republic | 93403 (71391,120323) | 665.74 (509.98,854.56) | 228258 (188006,254737) | 981.52 (815.26,1089.62) | -0.06 (-0.08,-0.04) | 144.38 |
| Democratic People's Republic of Korea | 73282 (57655,93865) | 534.03 (422.64,695.54) | 166218 (132256,214516) | 532.4 (422.66,687.42) | -0.83 (-0.98,-0.69) | 126.82 |
| Democratic Republic of the Congo | 51338 (39752,66862) | 429.1 (331.65,559.18) | 118526 (92188,158139) | 411.45 (320.33,546.46) | 1.44 (1.06,1.81) | 130.87 |
| Denmark | 69659 (53710,89658) | 817.12 (637.85,1043.61) | 111718 (88772,138463) | 908.22 (736.43,1119.51) | -0.03 (-0.05,-0.02) | 60.38 |
| Djibouti | 416 (324,545) | 388.29 (306.04,508.88) | 2213 (1710,2891) | 424.62 (335.81,555.29) | -0.18 (-0.2,-0.17) | 431.97 |
| Dominica | 389 (299,510) | 650.97 (507.12,849.2) | 544 (418,711) | 668.86 (514.54,861.99) | -0.06 (-0.24,0.13) | 39.73 |
| Dominican Republic | 22177 (17125,29173) | 662.46 (509.25,862.76) | 64889 (50825,84248) | 667.32 (520.85,869.63) | 0.31 (0.3,0.32) | 192.6 |
| Ecuador | 29616 (23224,38669) | 606.55 (471.16,785.92) | 98459 (77061,128281) | 615.7 (480.28,800.86) | 0.09 (0.08,0.11) | 232.46 |
| Egypt | 67055 (51335,87638) | 333.76 (255.54,434.8) | 184697 (143378,239672) | 393 (297.72,514.22) | 0 (-0.01,0.01) | 175.44 |
| El Salvador | 18470 (14488,24034) | 654.08 (510.09,850.26) | 42585 (33215,55380) | 665.9 (520.53,865.31) | 0.08 (0.07,0.1) | 130.57 |
| Equatorial Guinea | 634 (492,835) | 413.19 (321.21,541.73) | 1997 (1582,2592) | 487.69 (375.94,633.88) | 0.58 (0.55,0.6) | 215.03 |
| Eritrea | 3091 (2340,4067) | 371.42 (292.85,487.09) | 8704 (6755,11290) | 391.78 (304.41,516.89) | 0.07 (0.06,0.07) | 181.55 |
| Estonia | 11240 (8653,14638) | 544.37 (421.2,706.07) | 17269 (13159,22327) | 619.55 (480.95,800.04) | 0.58 (0.56,0.61) | 53.64 |
| Ethiopia | 63428 (49352,84190) | 406.05 (319.38,532.83) | 184880 (146237,239761) | 479.99 (377.48,630.3) | 0.17 (0.16,0.18) | 191.48 |
| Federated States of Micronesia | 242 (191,315) | 574.85 (450.09,745.62) | 353 (275,461) | 586.69 (458.47,759.65) | 0.48 (0.44,0.53) | 45.62 |
| Fiji | 1737 (1367,2298) | 607.48 (474.79,789.89) | 4434 (3483,5800) | 676.96 (527.93,884.27) | 0.16 (0.15,0.17) | 155.32 |
| Finland | 85278 (64676,106114) | 1168.45 (891.81,1441.77) | 117234 (94965,141038) | 862.54 (725.46,1013.76) | 0.68 (0.6,0.77) | 37.47 |
| France | 703229 (534466,921039) | 803.34 (620.29,1041.29) | 1081926 (820574,1416350) | 717.98 (554.42,925.96) | 0.37 (0.35,0.39) | 53.85 |
| Gabon | 2194 (1700,2905) | 432.99 (334.06,567.18) | 4065 (3180,5323) | 465.31 (360.35,610.14) | -1.12 (-1.23,-1.01) | 85.28 |
| Georgia | 33800 (25849,44479) | 558.89 (425.91,728.4) | 35412 (26991,46236) | 568.73 (436.23,738.62) | -0.5 (-0.57,-0.44) | 4.77 |
| Germany | 1225849 (937079,1593976) | 927.92 (719.81,1195.45) | 2155152 (1896612,2379617) | 1072.86 (943.99,1172.82) | 0.22 (0.22,0.23) | 75.81 |
| Ghana | 20084 (15703,26388) | 392.93 (305.98,515.13) | 60669 (47244,80034) | 432.42 (334.33,567.15) | 0.11 (0.08,0.14) | 202.07 |
| Greece | 105797 (87036,133969) | 686.94 (566.89,865.16) | 175318 (132665,230098) | 662.08 (510.11,855.56) | 0 (-0.02,0.03) | 65.71 |
| Greenland | 292 (228,377) | 1087.86 (839.27,1403.54) | 617 (478,795) | 1014.83 (775.76,1305.09) | 0.38 (0.16,0.6) | 111.34 |
| Grenada | 498 (382,652) | 642.5 (501.35,833.65) | 695 (538,916) | 653.72 (506.53,851.89) | 0.23 (0.17,0.3) | 39.58 |
| Guam | 369 (289,473) | 597.15 (469.33,773.68) | 1453 (1138,1871) | 672.25 (525.78,866.79) | -0.32 (-0.58,-0.06) | 294.12 |
| Guatemala | 17778 (13983,23674) | 604.89 (470,790.52) | 66296 (51813,86475) | 636.34 (499.2,825.44) | -0.15 (-0.23,-0.07) | 272.9 |
| Guinea | 11006 (8485,14476) | 370.46 (286.63,484.66) | 19024 (14744,25081) | 387.59 (300.45,506.19) | 0 (-0.04,0.05) | 72.85 |
| Guinea-Bissau | 1218 (950,1606) | 379.44 (293.67,497.66) | 2225 (1727,2948) | 394.7 (304.39,521.63) | 0.36 (0.32,0.41) | 82.76 |
| Guyana | 2254 (1769,2967) | 671.43 (519.76,877.4) | 3827 (2984,5099) | 667.24 (519.54,874.3) | 0.24 (0.21,0.28) | 69.78 |
| Haiti | 17161 (13462,22578) | 637.48 (497.01,823.22) | 39243 (30771,51649) | 654.08 (507,860.17) | 0.13 (0.13,0.14) | 128.67 |
| Honduras | 12036 (9402,15784) | 659.29 (513.99,858.55) | 37962 (29758,49433) | 664.18 (516.7,857.85) | 0.12 (0.11,0.12) | 215.41 |
| Hungary | 101902 (76999,132894) | 681.32 (519.83,880.64) | 118850 (90414,156204) | 574.65 (443.45,748.72) | -0.01 (-0.03,0.01) | 16.63 |
| Iceland | 2087 (1581,2704) | 704.04 (536.96,912.64) | 4788 (3892,5821) | 793.27 (651.04,960.17) | 0.11 (0.1,0.12) | 129.41 |
| India | 1860594 (1442729,2458704) | 521.35 (402.59,685.09) | 5612366 (4314558,7360330) | 533.92 (412.71,700) | 0.02 (0.01,0.03) | 201.64 |
| Indonesia | 560360 (442858,730937) | 700.63 (547.24,905.66) | 1474160 (1152713,1922564) | 728.2 (569.74,942.29) | -0.6 (-0.66,-0.55) | 163.07 |
| Iraq | 26906 (20773,34733) | 373.36 (285.05,485.8) | 78701 (61203,102060) | 406.12 (308.47,532.03) | 0.22 (0.14,0.3) | 192.5 |
| Ireland | 31562 (23747,40777) | 757.71 (578.57,979.99) | 54616 (42201,70424) | 666.48 (516.95,858.36) | 0.09 (0.08,0.11) | 73.04 |
| Islamic Republic of Iran | 75833 (58814,99095) | 385.97 (296.24,507.56) | 294249 (230024,382166) | 425.39 (327.17,559.23) | 0.15 (0.14,0.16) | 288.02 |
| Israel | 41616 (31818,54187) | 848.35 (656.52,1096.61) | 147352 (122034,167473) | 1155.51 (958.33,1312.39) | 0.23 (0.18,0.28) | 254.07 |
| Italy | 824078 (633970,1072624) | 897.86 (697.07,1163.46) | 1327156 (1001901,1743600) | 821.57 (632.6,1065.6) | 0.26 (0.24,0.28) | 61.05 |
| Jamaica | 11880 (9135,15489) | 647.52 (501.68,840.64) | 21347 (16750,27574) | 675.56 (527.03,877.87) | -0.86 (-0.98,-0.73) | 79.68 |
| Japan | 873570 (685978,1135914) | 519.91 (411.63,671.77) | 1498702 (1171946,1964169) | 391.87 (310.63,511.22) | 1.46 (1.15,1.77) | 71.56 |
| Jordan | 3649 (2816,4743) | 354.23 (268.4,466.12) | 23983 (18451,31112) | 396.61 (300.63,520.3) | -0.6 (-0.69,-0.51) | 557.28 |
| Kazakhstan | 65048 (50303,84806) | 562.81 (428.43,731.6) | 96397 (74221,126067) | 589.25 (453.42,768.59) | 0.15 (0.11,0.18) | 48.19 |
| Kenya | 32479 (25576,42395) | 455.99 (357.55,595.62) | 90501 (71144,118517) | 467.05 (366.94,610.02) | -0.89 (-1.16,-0.61) | 178.64 |
| Kingdom of Eswatini | 1042 (813,1372) | 445.49 (345.52,584.17) | 2108 (1632,2790) | 468.81 (364.74,615.22) | 0.35 (0.34,0.37) | 102.38 |
| Kiribati | 170 (133,221) | 577.26 (450.63,748.84) | 347 (272,457) | 598.52 (466.2,771.38) | 0.16 (0.13,0.18) | 103.65 |
| Kuwait | 1721 (1337,2199) | 369.85 (282.78,483.44) | 9836 (7697,12638) | 408.07 (310.58,530.22) | 0.07 (0.06,0.08) | 471.57 |
| Kyrgyzstan | 13329 (10299,17484) | 478.91 (366.47,626.57) | 20779 (16256,27052) | 491.16 (377.25,639.22) | 0.09 (0.07,0.11) | 55.89 |
| Lao People's Democratic Republic | 9988 (7851,13152) | 587.21 (457.7,766.12) | 23869 (18913,31406) | 605.11 (475.86,782.28) | 0.29 (0.24,0.35) | 138.98 |
| Latvia | 18682 (14270,24444) | 516.84 (396.14,673.25) | 25774 (22299,29649) | 629.57 (547.41,715.02) | 0.05 (0.04,0.06) | 37.96 |
| Lebanon | 6336 (4939,8255) | 342.25 (261.21,444.6) | 24591 (18725,32516) | 387.14 (298.49,511.08) | 0.1 (0.08,0.12) | 288.11 |
| Lesotho | 3040 (2351,3994) | 403.46 (311.48,530.49) | 3809 (2967,5053) | 423.73 (327.59,555.16) | 0.97 (0.8,1.14) | 25.29 |
| Liberia | 4038 (3146,5396) | 397.36 (308.62,524.77) | 7117 (5638,9316) | 408.89 (317.19,535.39) | 0.44 (0.42,0.45) | 76.26 |
| Libya | 6061 (4645,7875) | 367.31 (276.5,479.71) | 17554 (13696,22623) | 398.27 (304.76,518.53) | 0.14 (0.13,0.14) | 189.65 |
| Lithuania | 25740 (19772,33473) | 567.12 (437.53,735.84) | 37996 (28984,48993) | 634.2 (495.02,808.33) | 0.1 (0.09,0.11) | 47.62 |
| Luxembourg | 4288 (3610,5095) | 763.59 (647.3,906.09) | 8302 (7313,9305) | 751.62 (663.73,841.08) | 0.24 (0.21,0.26) | 93.62 |
| Madagascar | 16984 (13349,22344) | 391.91 (308.5,516.98) | 37729 (29479,49128) | 417.24 (327.3,548.36) | 0.42 (0.38,0.46) | 122.15 |
| Malawi | 13014 (10145,17325) | 406.12 (318.51,530.77) | 27955 (21964,36667) | 445.44 (350.05,586.14) | 0.01 (-0.04,0.05) | 114.81 |
| Malaysia | 52682 (42117,68276) | 631.34 (495.34,821.09) | 174176 (137798,228296) | 673.16 (526.92,873.05) | 0.22 (0.21,0.23) | 230.62 |
| Maldives | 424 (329,553) | 601.49 (471.53,786.4) | 1871 (1497,2409) | 619.06 (486.08,803.07) | 0.29 (0.27,0.32) | 340.77 |
| Mali | 11800 (9185,15919) | 363.12 (281.72,476.4) | 28145 (22007,37160) | 384.98 (298.11,507.1) | 0.34 (0.29,0.38) | 138.51 |
| Malta | 2836 (2179,3655) | 676.78 (521.13,871.7) | 6323 (5524,7076) | 598.49 (529.84,673.49) | 0.05 (0.04,0.07) | 122.92 |
| Marshall Islands | 73 (57,93) | 543.46 (423.67,699.52) | 157 (122,206) | 578.23 (451.61,751.27) | 0.18 (0.17,0.19) | 115.38 |
| Mauritania | 3395 (2607,4514) | 383.24 (295.57,505.2) | 8034 (6250,10647) | 419.11 (324.19,548.09) | -0.06 (-0.35,0.23) | 136.67 |
| Mauritius | 3919 (3071,5108) | 621.51 (488.13,813.67) | 11425 (8978,15014) | 642.42 (500.8,837.33) | 0.18 (0.17,0.19) | 191.53 |
| Mexico | 278511 (219192,361872) | 732.26 (571.33,951.32) | 899391 (705327,1167818) | 746.46 (582.69,967.96) | 0.28 (0.27,0.3) | 222.93 |
| Mongolia | 4854 (3718,6266) | 507.69 (387.87,661.82) | 10601 (8189,13897) | 544.58 (413.48,704.44) | -0.02 (-0.08,0.03) | 118.38 |
| Montenegro | 3723 (2851,4875) | 622.42 (474.22,809.12) | 5917 (4506,7731) | 597.77 (457.54,773.59) | 0.12 (0.1,0.14) | 58.91 |
| Morocco | 45335 (34423,58980) | 360.21 (273.2,470.75) | 117079 (89916,151800) | 383.34 (292.51,505.95) | 0.05 (0.04,0.06) | 158.25 |
| Mozambique | 20053 (15801,26456) | 397.84 (310.59,525.54) | 39677 (30931,51541) | 427.96 (333.79,561.38) | -0.41 (-0.46,-0.35) | 97.86 |
| Myanmar | 118341 (93009,156133) | 613.93 (479.1,797.7) | 267795 (211909,352206) | 614.44 (480.43,795.77) | 0.25 (0.23,0.27) | 126.29 |
| Namibia | 2284 (1755,2994) | 432.25 (335.34,566.54) | 5143 (3995,6875) | 443.93 (340.35,581.29) | -0.08 (-0.12,-0.05) | 125.21 |
| Nepal | 34654 (26729,45519) | 471.77 (362.87,619.63) | 96213 (73203,125250) | 471.74 (359.9,618.89) | 0.12 (0.09,0.15) | 177.64 |
| Netherlands | 172361 (156082,189738) | 834.04 (753,922.78) | 287028 (243038,332612) | 769.98 (654.22,894.08) | 0.2 (0.19,0.22) | 66.53 |
| New Zealand | 32004 (25316,40250) | 806.03 (643.22,1015.05) | 72708 (60818,88247) | 835.21 (704.87,1004.71) | 0.01 (0,0.02) | 127.18 |
| Nicaragua | 9056 (7066,11755) | 657.62 (505.51,861.44) | 30250 (23880,39026) | 661.52 (516.78,851.32) | 0.05 (0.04,0.06) | 234.05 |
| Niger | 8171 (6367,10826) | 368.55 (285.62,485.65) | 25954 (20174,34414) | 383.57 (295.92,504.59) | 0.06 (0.04,0.09) | 217.62 |
| Nigeria | 153286 (119379,203690) | 399.72 (310.99,523.95) | 353475 (278100,466330) | 461.68 (358.97,602.32) | 0.02 (-0.05,0.09) | 130.6 |
| Northern Mariana Islands | 82 (64,104) | 633.21 (496.4,821.86) | 294 (229,378) | 662.43 (515.37,853.03) | -0.59 (-0.72,-0.46) | 260.21 |
| Norway | 57867 (44003,75498) | 798.36 (620.77,1032.65) | 79412 (61345,102809) | 761.48 (589.35,985.49) | 0.2 (0.1,0.29) | 37.23 |
| Oman | 1615 (1248,2101) | 292.89 (225.07,380.76) | 5827 (4557,7637) | 365.26 (276.39,482.95) | 0.03 (0.02,0.05) | 260.77 |
| Pakistan | 270729 (206528,355785) | 554.73 (429.37,728.7) | 548174 (427141,726637) | 558.61 (429.5,735.69) | 0.13 (0.11,0.15) | 102.48 |
| Palestine | 2437 (1859,3179) | 323.2 (244.98,422.73) | 7197 (5525,9405) | 347.45 (265.23,453.98) | 0.57 (0.52,0.62) | 195.36 |
| Panama | 9494 (7472,12317) | 676.55 (528.06,881.82) | 30751 (24204,39735) | 691.05 (542.43,895.33) | 0.27 (0.23,0.31) | 223.92 |
| Papua New Guinea | 7335 (5716,9600) | 532.9 (418.45,697.52) | 21815 (17006,28137) | 543.71 (426.86,705.94) | -0.08 (-0.11,-0.05) | 197.41 |
| Paraguay | 16763 (13141,21472) | 797.07 (624.59,1027.49) | 43040 (33767,55877) | 769.35 (602.53,992.61) | 0.1 (0.06,0.13) | 156.75 |
| Peru | 66121 (52636,86420) | 597.59 (472.59,772.26) | 220883 (171496,283755) | 668.37 (516.03,860.23) | -0.16 (-0.21,-0.12) | 234.06 |
| Philippines | 160947 (126928,211314) | 648.69 (510.61,837.57) | 466916 (369507,610795) | 653.73 (513.13,843.12) | 0.84 (0.77,0.91) | 190.11 |
| Plurinational State of Bolivia | 16730 (13176,22025) | 595.3 (464.6,774.97) | 51917 (40535,67894) | 622.3 (488.06,809.65) | 0.06 (0.04,0.07) | 210.33 |
| Poland | 276686 (211770,358761) | 640.38 (488.94,828.18) | 648195 (491254,846914) | 866.31 (664.47,1117.94) | 0.29 (0.26,0.32) | 134.27 |
| Portugal | 105934 (80401,138675) | 758.21 (585.05,989.75) | 192005 (161459,229225) | 695.71 (591.04,820.14) | 0.18 (0.16,0.2) | 81.25 |
| Principality of Monaco | 602 (451,792) | 787.04 (609.64,1021.06) | 754 (573,971) | 718.17 (557.76,916.81) | 0.1 (0.08,0.12) | 25.26 |
| Puerto Rico | 25706 (19933,33640) | 709.64 (552.95,924.66) | 56494 (43078,73735) | 715.09 (552.46,927.13) | 0.05 (0.04,0.07) | 119.77 |
| Qatar | 298 (228,389) | 363.49 (278.22,475.53) | 3005 (2251,3953) | 402.59 (305.76,529.29) | -0.14 (-0.17,-0.11) | 908.18 |
| Republic of Cabo Verde | 943 (728,1248) | 397.4 (310.11,520.15) | 1754 (1380,2302) | 419.74 (323.1,551.73) | 0.49 (0.43,0.54) | 85.99 |
| Republic of C么te d'Ivoire | 12409 (9635,16308) | 402.26 (310.63,527.53) | 38549 (30111,50565) | 424.45 (328.78,553.75) | -0.01 (-0.02,0) | 210.66 |
| Republic of Korea | 167647 (132028,217480) | 610.53 (486.7,785.88) | 628474 (544533,739494) | 675.14 (584.7,790.59) | 0.25 (-0.03,0.53) | 274.88 |
| Republic of Moldova | 23013 (17873,30099) | 551.37 (427.29,709.11) | 37430 (28839,48105) | 622.47 (480.68,796.46) | -0.27 (-0.31,-0.23) | 62.65 |
| Republic of Nauru | 23 (18,30) | 642.55 (498.21,838.02) | 31 (25,40) | 671.41 (528.4,873.97) | 0.02 (0.01,0.04) | 37.68 |
| Republic of Niue | 14 (11,19) | 616.15 (483.25,798.37) | 14 (11,19) | 677.63 (531.4,878.21) | 0.26 (0.22,0.3) | -0.78 |
| Republic of Palau | 49 (39,65) | 585.01 (458.06,756.17) | 125 (98,163) | 646.55 (506.74,842.07) | 0.52 (0.36,0.68) | 153.23 |
| Republic of San Marino | 293 (225,382) | 800.69 (623.05,1036.98) | 583 (442,761) | 720.08 (555.9,936.44) | 0.45 (0.43,0.46) | 99.04 |
| Republic of the Gambia | 1153 (900,1502) | 394.31 (307.81,516.75) | 3491 (2752,4643) | 411.42 (317.56,536.48) | -1.17 (-1.44,-0.9) | 202.8 |
| Romania | 153776 (118560,200226) | 568.65 (435.33,734.23) | 162826 (141283,191969) | 412.59 (359.76,488) | 0.43 (0.33,0.52) | 5.89 |
| Russian Federation | 1053310 (820702,1363643) | 599.94 (467.24,774.28) | 1639057 (1272601,2138452) | 673.56 (526.09,872.26) | 0.11 (0.1,0.11) | 55.61 |
| Rwanda | 9685 (7590,12457) | 414.73 (326,538.65) | 23218 (18165,30751) | 431.09 (337.04,563.82) | -0.02 (-0.04,0.01) | 139.74 |
| Saint Kitts and Nevis | 253 (194,329) | 653.45 (516.25,841.33) | 406 (319,530) | 657.86 (511.85,859.19) | 0.01 (-0.01,0.03) | 60.28 |
| Saint Lucia | 583 (447,760) | 688.42 (532.55,895.22) | 1618 (1260,2099) | 680.63 (528.46,880.89) | 0.18 (0.17,0.19) | 177.35 |
| Saint Vincent and the Grenadines | 443 (341,581) | 631.81 (489.77,823.79) | 939 (732,1231) | 670.59 (523.25,876.28) | 0.07 (0.05,0.1) | 112.2 |
| Samoa | 451 (356,591) | 612.88 (479.08,796.86) | 825 (649,1086) | 637.38 (497.38,825.04) | -0.44 (-0.48,-0.4) | 82.81 |
| Sao Tome and Principe | 234 (182,309) | 391.01 (302,510.48) | 413 (324,548) | 431.62 (332.48,560.79) | 0.3 (0.26,0.33) | 76.11 |
| Saudi Arabia | 15737 (12130,20450) | 330.72 (250.33,435.24) | 54167 (42125,71479) | 391.86 (301.89,511.74) | 0.53 (0.51,0.54) | 244.19 |
| Senegal | 11310 (8799,15063) | 404.14 (314.98,526.95) | 28239 (22128,37630) | 416.56 (324.04,545.14) | 0.1 (0.07,0.13) | 149.69 |
| Serbia | 56918 (44033,74679) | 549.73 (422.16,715.09) | 70792 (59259,87626) | 409.9 (345.15,506.86) | -1.01 (-1.31,-0.72) | 24.38 |
| Seychelles | 354 (278,458) | 630.72 (495.41,812.41) | 696 (551,905) | 644.8 (506.5,834.04) | 0.04 (0.02,0.06) | 96.54 |
| Sierra Leone | 8193 (6376,10749) | 441.68 (342.89,578.08) | 14273 (11176,18823) | 438.39 (341.01,578.66) | -0.08 (-0.12,-0.05) | 74.21 |
| Singapore | 10361 (8190,13361) | 496.12 (394.24,643.05) | 36795 (28982,47620) | 430.26 (339.42,557.39) | -0.62 (-0.72,-0.51) | 255.14 |
| Slovakia | 50960 (42632,58858) | 848.64 (713.21,976.42) | 90634 (74283,106522) | 917.33 (758.01,1072.43) | 0.16 (0.08,0.24) | 77.85 |
| Slovenia | 15036 (11602,19562) | 610.78 (474.02,792.53) | 28595 (25053,32065) | 617.35 (543.74,691.9) | 0.05 (0.01,0.09) | 90.18 |
| Socialist Republic of Viet Nam | 214245 (169209,280476) | 581.97 (454.92,753.15) | 581496 (457784,771131) | 644.9 (502.66,844.07) | 0.06 (0.05,0.07) | 171.42 |
| Solomon Islands | 583 (457,752) | 551.7 (429.29,719.58) | 1594 (1258,2087) | 568.32 (443.6,747.82) | 0.15 (0.14,0.16) | 173.26 |
| Somalia | 6910 (5439,8954) | 374.38 (294.47,490.01) | 18687 (14347,24314) | 392.02 (308.37,516.94) | -0.08 (-0.09,-0.06) | 170.43 |
| South Africa | 98210 (77197,127867) | 532.23 (414.73,693.35) | 217357 (169171,286785) | 526.19 (408.32,689.91) | 0.3 (0.28,0.32) | 121.32 |
| South Sudan | 8739 (6834,11524) | 388.78 (304.99,513.39) | 13605 (10696,17831) | 423.26 (332.01,554.83) | 0.05 (-0.09,0.19) | 55.68 |
| Spain | 489404 (384802,625445) | 876.31 (690.91,1108.62) | 902888 (806291,1004786) | 863.92 (780.25,960.23) | 0.17 (0.15,0.19) | 84.49 |
| Sri Lanka | 55986 (44216,73510) | 600.31 (472.18,777.41) | 163229 (128662,213322) | 632.17 (494.65,817.05) | 0.55 (0.54,0.57) | 191.55 |
| Sudan | 26219 (19909,34245) | 336.41 (256.33,438.22) | 63916 (49242,83110) | 394.5 (298.41,515.66) | -0.08 (-0.09,-0.07) | 143.78 |
| Suriname | 1552 (1216,2030) | 662.16 (514.71,863.35) | 3954 (3098,5171) | 651.22 (508.16,840.34) | 1.05 (0.94,1.17) | 154.81 |
| Sweden | 163917 (128606,209330) | 1010.87 (810.08,1286.9) | 360875 (270489,465091) | 1529.82 (1166.57,1943.26) | 0.3 (-0.1,0.7) | 120.16 |
| Switzerland | 51170 (39839,65184) | 463.76 (360.67,586.53) | 84810 (74812,97781) | 439.01 (388.91,505.6) | 0.24 (0.23,0.26) | 65.74 |
| Syrian Arab Republic | 15095 (11605,19661) | 348.36 (264.02,453.87) | 41606 (32220,54570) | 377.81 (288.12,497.32) | -0.18 (-0.23,-0.13) | 175.63 |
| Taiwan (Province of China) | 90993 (73354,115877) | 645.3 (520.86,815.93) | 273226 (222314,341516) | 629.83 (513.33,784.23) | 0.13 (0.1,0.15) | 200.27 |
| Tajikistan | 11955 (9331,15431) | 476.78 (369.22,619.07) | 23850 (18509,31221) | 489.48 (373.73,641.48) | 0.16 (0.14,0.18) | 99.49 |
| Thailand | 176405 (139408,229043) | 577.24 (454.35,745.36) | 661374 (516881,864805) | 603.8 (470.51,784.54) | 0.14 (0.13,0.15) | 274.92 |
| The former Yugoslav Republic of Macedonia | 10256 (7924,13365) | 593.55 (455.11,776.64) | 18793 (14283,24572) | 572.89 (435.57,741.4) | 0.05 (0.04,0.07) | 83.23 |
| Timor-Leste | 1280 (1023,1638) | 585.93 (462.52,761.76) | 4680 (3685,6092) | 605.83 (476.38,787.01) | 0.36 (0.34,0.39) | 265.59 |
| Togo | 4094 (3207,5424) | 403.31 (311.69,529.91) | 12756 (9931,16740) | 411.66 (322.28,540.75) | 0.27 (0.24,0.29) | 211.59 |
| Tokelau | 7 (6,9) | 559.76 (436.38,722.71) | 9 (7,12) | 629.56 (494.07,812.57) | 0.08 (0.06,0.1) | 32.31 |
| Tonga | 292 (231,381) | 600.15 (470.99,773.65) | 507 (400,655) | 654.77 (516.14,842.08) | 0.24 (0.23,0.26) | 73.52 |
| Trinidad and Tobago | 5672 (4370,7442) | 710.06 (551.1,922.55) | 14037 (10919,18399) | 732.58 (567.98,954.23) | -0.84 (-1,-0.69) | 147.48 |
| Tunisia | 14175 (10853,18659) | 335.66 (257.82,444.15) | 45069 (34412,58372) | 365.01 (276.78,471.64) | 0.4 (0.39,0.41) | 217.96 |
| Turkey | 99437 (77991,127594) | 332.71 (262.14,425.52) | 247837 (221168,275262) | 282.89 (252.21,314.68) | 0.31 (0.28,0.33) | 149.24 |
| Turkmenistan | 8444 (6460,11054) | 501.72 (380.44,654.49) | 19896 (15619,25583) | 563.75 (432.79,729.21) | 0 (-0.04,0.04) | 135.64 |
| Tuvalu | 30 (24,40) | 548.68 (433.76,708.17) | 57 (45,75) | 609.07 (478.07,796.75) | 0.19 (0.16,0.23) | 88.73 |
| Uganda | 24063 (18872,31962) | 431.96 (338.73,567.67) | 55891 (43879,73062) | 442.48 (347.59,586.14) | 0.41 (0.33,0.49) | 132.27 |
| Ukraine | 397837 (308635,521139) | 559.63 (438.88,727.55) | 468750 (362555,614415) | 586.74 (457.73,760.23) | -0.07 (-0.2,0.05) | 17.82 |
| United Arab Emirates | 1294 (990,1674) | 381.72 (289.37,494.96) | 13475 (9821,18266) | 424.69 (326.48,554.82) | 0.26 (0.07,0.45) | 941.59 |
| United Kingdom of Great Britain and Northern Ireland | 589601 (466035,750521) | 620.1 (495.41,784.31) | 888645 (725229,1093517) | 648.32 (535.59,793.81) | 0.57 (0.5,0.64) | 50.72 |
| United Republic of Tanzania | 36073 (28046,47724) | 387.72 (303.94,510.65) | 99864 (78447,132449) | 445.22 (349.86,586.08) | 0.07 (0.06,0.07) | 176.84 |
| United States of America | 2909341 (2226181,3751437) | 875.02 (675.05,1125.91) | 6373868 (5938751,6852944) | 1040.36 (973.52,1116.25) | -0.32 (-0.36,-0.27) | 119.08 |
| United States Virgin Islands | 525 (409,695) | 695 (537.89,902.89) | 1367 (1038,1806) | 710.98 (550.75,926.06) | 0.21 (0.2,0.23) | 160.32 |
| Uruguay | 18817 (14625,24726) | 474.49 (372.7,619.64) | 26364 (20167,34763) | 445.88 (345.48,579.21) | 0.02 (-0.01,0.05) | 40.11 |
| Uzbekistan | 49809 (38683,65011) | 460.42 (354.54,596.53) | 112675 (87824,148398) | 496.56 (380.6,651.61) | -0.2 (-0.21,-0.19) | 126.21 |
| Vanuatu | 318 (247,418) | 643.66 (500.24,835.55) | 942 (739,1229) | 658.57 (516.53,852.91) | 0.43 (0.38,0.48) | 196.34 |
| Yemen | 11693 (8974,15102) | 307.38 (234.12,401.08) | 38432 (29592,49671) | 337.23 (255.75,443.55) | 0.32 (0.31,0.33) | 228.68 |
| Zambia | 9243 (7217,12053) | 387.55 (304.25,508.42) | 23356 (18374,30162) | 404.89 (320.47,528.63) | 0.14 (0.13,0.16) | 152.69 |
| Zimbabwe | 14595 (11343,19291) | 430.38 (334.6,559.29) | 24452 (18907,32514) | 444.98 (344.65,580.89) | 0.08 (0.07,0.1) | 67.54 |

AF/AFL, Atrial fibrillation (AF)/atrial flutter (AFL); CI, confidence interval; EAPC, estimated annual percentage change; UI, uncertainty interval.

Supplementary Table 6 Death of AF/AFL Between 1990 and 2021 in 204 countries

| Location | Death in 1990 | | Death in 2021 | | 1990-2021 EAPC | 1990-2021 change |
| --- | --- | --- | --- | --- | --- | --- |
|  | Number(95%UI) | Rate(95%UI) | Number(95%UI) | Rate(95%UI) | EAPC_95%CI |  |
| Global | 114540 (101326,127155) | 4.24 (3.69,4.71) | 338947 (288954,368613) | 4.36 (3.69,4.75) | 0.1(0.06,0.13) | 195.92 |
| Sex |  |  |  |  |  |  |
| Males | 42677 (37233,46516) | 4.2 (3.65,4.59) | 134700 (120296,145862) | 4.44 (3.94,4.81) | 0.21(0.18,0.24) |  |
| Females | 71862 (63274,81196) | 4.25 (3.66,4.79) | 204247 (167703,228405) | 4.29 (3.53,4.8) | 0.02(-0.02,0.06) |  |
| SDI region |  |  |  |  |  |  |
| High SDI | 50188 (44165,53072) | 4.74 (4.14,5.03) | 125622 (103230,137669) | 4.66 (3.88,5.08) | -0.01(-0.06,0.04) | 150.3 |
| High-middle SDI | 28089 (25067,30559) | 4.23 (3.69,4.63) | 79214 (67067,88012) | 4.29 (3.62,4.77) | 0.08(0.03,0.13) | 182.01 |
| Middle SDI | 22063 (19086,25973) | 4.18 (3.6,4.92) | 83853 (70992,94818) | 4.26 (3.57,4.83) | -0.06(-0.13,0.02) | 280.07 |
| Low-middle SDI | 10458 (7579,14095) | 3.07 (2.26,4.06) | 39263 (32069,46278) | 4.08 (3.34,4.81) | 1(0.91,1.08) | 275.45 |
| Low SDI | 3568 (2077,5030) | 3.05 (1.79,4.32) | 10623 (7284,13876) | 3.74 (2.57,4.9) | 0.87(0.63,1.12) | 197.77 |
| GBD region |  |  |  |  |  |  |
| Andean Latin America | 740 (634,855) | 4.61 (3.97,5.3) | 2079 (1699,2494) | 3.81 (3.11,4.57) | -0.78(-0.94,0.61) | 180.89 |
| Australasia | 1436 (1281,1525) | 6.99 (6.16,7.45) | 4337 (3529,4789) | 6.58 (5.39,7.25) | 0(-0.14,0.14) | 202.05 |
| Caribbean | 1098 (985,1194) | 5.59 (4.99,6.07) | 2731 (2349,3048) | 4.86 (4.2,5.42) | -0.42(-0.51,0.33) | 148.65 |
| Central Asia | 684 (596,799) | 1.87 (1.61,2.2) | 1386 (1241,1519) | 2.37 (2.09,2.6) | 0.69(0.43,0.95) | 102.62 |
| Central Europe | 5931 (5508,6253) | 5.1 (4.66,5.4) | 11104 (9859,11914) | 4.55 (4.03,4.88) | -0.21(-0.41,0.02) | 87.23 |
| Central Latin America | 2816 (2613,2927) | 4.98 (4.56,5.21) | 10354 (8980,11386) | 4.55 (3.95,5.01) | -0.32(-0.38,0.26) | 267.66 |
| Central Sub-Saharan Africa | 439 (278,633) | 4.11 (2.55,5.93) | 1311 (886,1982) | 4.67 (3.16,7.09) | 0.36(0.19,0.54) | 198.96 |
| East Asia | 17272 (14013,21292) | 4.94 (3.92,6.07) | 67666 (54232,80814) | 4.3 (3.42,5.18) | -0.63(-0.8,0.45) | 291.76 |
| Eastern Europe | 8133 (7308,8953) | 3.87 (3.44,4.3) | 15697 (14042,17056) | 4.33 (3.87,4.71) | 0.26(0.15,0.37) | 93 |
| Eastern Sub-Saharan Africa | 1263 (687,1765) | 3.17 (1.73,4.55) | 3166 (1927,4731) | 3.28 (1.97,4.89) | 0.06(-0.08,0.2) | 150.63 |
| High_income Asia Pacific | 5018 (4465,5373) | 3.06 (2.68,3.3) | 17112 (13415,19331) | 2.46 (2.01,2.74) | -1.27(-1.61,0.93) | 241 |
| High_income North America | 14544 (12556,15549) | 3.96 (3.41,4.24) | 39066 (32116,42759) | 5.15 (4.27,5.61) | 0.79(0.72,0.87) | 168.6 |
| North Africa And Middle East | 3374 (2613,4241) | 3.5 (2.68,4.4) | 11182 (9280,12594) | 3.92 (3.19,4.44) | 0.53(0.34,0.72) | 231.44 |
| Oceania | 65 (44,82) | 4.48 (3.27,5.61) | 185 (132,238) | 4.29 (3.16,5.46) | -0.19(-0.25,0.13) | 186.33 |
| South Asia | 7594 (4655,11542) | 2.38 (1.46,3.62) | 36165 (27041,46073) | 3.69 (2.75,4.71) | 1.58(1.38,1.78) | 376.22 |
| Southeast Asia | 6120 (5116,7566) | 4.24 (3.5,5.3) | 22401 (18974,25759) | 5.27 (4.39,6.12) | 0.61(0.49,0.74) | 266.01 |
| Southern Latin America | 1317 (1195,1407) | 3.61 (3.22,3.88) | 3299 (2853,3562) | 3.53 (3.06,3.81) | 0.74(0.38,1.11) | 150.5 |
| Southern Sub-Saharan Africa | 511 (417,626) | 2.82 (2.26,3.48) | 1413 (1228,1551) | 4 (3.39,4.44) | 1.14(0.82,1.46) | 176.56 |
| Tropical Latin America | 2727 (2419,2896) | 4.99 (4.29,5.36) | 11540 (9640,12679) | 4.86 (4.05,5.34) | -0.12(-0.21,0.03) | 323.14 |
| Werstern Europe | 31335 (27682,33261) | 5.43 (4.75,5.77) | 72184 (58846,79292) | 5.52 (4.56,6.04) | 0.31(0.24,0.38) | 130.36 |
| Werstern Sub-Saharan Africa | 2121 (1585,2724) | 4.51 (3.37,5.78) | 4568 (3662,5235) | 4.46 (3.65,5.13) | -0.23(-0.32,0.14) | 115.35 |

| Afghanistan | 113 (59,190) | 2.75 (1.46,4.55) | 179 (111,264) | 3.28 (2.04,4.86) | 0.66 (0.63,0.7) | 59.22 |
| --- | --- | --- | --- | --- | --- | --- |
| Albania | 59 (51,69) | 4.02 (3.41,4.65) | 181 (132,229) | 4.72 (3.41,6.04) | 0.92 (0.73,1.1) | 205.03 |
| Algeria | 244 (173,322) | 5.32 (3.83,6.82) | 1114 (824,1390) | 5.97 (4.5,7.24) | 1.15 (0.88,1.42) | 355.81 |
| American Samoa | 1 (1,1) | 6.07 (4.86,7.79) | 2 (1,3) | 6.46 (4.61,8.54) | 0.38 (0.33,0.44) | 196.02 |
| Andorra | 2 (1,3) | 4.8 (3.41,6.92) | 7 (5,9) | 3.66 (2.64,4.82) | -0.59 (-0.77,-0.4) | 262.21 |
| Angola | 74 (48,108) | 3.85 (2.52,5.65) | 279 (194,392) | 4.86 (3.35,6.83) | 0.64 (0.55,0.73) | 278.02 |
| Antigua and Barbuda | 4 (4,5) | 7 (6.2,7.77) | 5 (5,6) | 6.61 (5.97,7.14) | -0.06 (-0.28,0.17) | 24.4 |
| Argentina | 946 (851,1019) | 3.75 (3.32,4.06) | 2027 (1752,2197) | 3.42 (2.95,3.7) | 0.49 (0.15,0.83) | 114.21 |
| Armenia | 31 (27,35) | 1.52 (1.31,1.77) | 108 (93,122) | 2.53 (2.18,2.86) | 2.04 (1.67,2.4) | 249.82 |
| Australia | 1155 (1033,1227) | 6.79 (5.98,7.24) | 3608 (2927,3990) | 6.42 (5.24,7.08) | 0 (-0.18,0.17) | 212.33 |
| Austria | 659 (592,698) | 5.55 (4.94,5.9) | 1651 (1349,1818) | 6.75 (5.58,7.41) | 0.78 (0.47,1.09) | 150.51 |
| Azerbaijan | 59 (48,77) | 1.65 (1.34,2.14) | 124 (93,154) | 1.79 (1.39,2.21) | 0.76 (0.51,1) | 111.02 |
| Bahrain | 4 (2,5) | 6.97 (3.4,9.27) | 15 (8,20) | 5.66 (2.58,7.9) | 0.17 (-0.06,0.41) | 299.14 |
| Bangladesh | 1026 (567,1542) | 3.26 (1.75,4.9) | 4552 (3252,6400) | 5 (3.64,6.99) | -0.74 (-1.17,-0.31) | 343.51 |
| Barbados | 17 (15,18) | 5.84 (5.3,6.29) | 29 (24,34) | 5.74 (4.81,6.66) | 1.21 (0.83,1.59) | 73.68 |
| Belarus | 415 (354,477) | 3.64 (3.08,4.18) | 677 (566,781) | 4.06 (3.41,4.69) | 0.04 (-0.13,0.2) | 63.1 |
| Belgium | 694 (605,747) | 4.57 (3.93,4.93) | 1396 (1088,1557) | 4.21 (3.35,4.67) | 0.17 (0.09,0.25) | 101.19 |
| Belize | 4 (3,4) | 3.96 (3.23,4.8) | 11 (9,12) | 4.61 (3.97,5.16) | 0.16 (-0.09,0.4) | 205.06 |
| Benin | 42 (29,54) | 3.09 (2.09,3.93) | 108 (82,137) | 3.66 (2.75,4.62) | 0.3 (-0.15,0.76) | 154.56 |
| Bermuda | 3 (3,4) | 6.61 (5.63,7.4) | 7 (6,9) | 4.45 (3.69,5.36) | 0.6 (0.55,0.66) | 131.89 |
| Bhutan | 3 (2,5) | 2.64 (1.42,4.22) | 22 (15,30) | 4.47 (3.09,6.28) | -1.47 (-1.55,-1.39) | 571.05 |
| Bolivarian Republic of Venezuela | 327 (292,355) | 4.65 (4.08,5.09) | 1204 (948,1481) | 4.58 (3.63,5.6) | 1.89 (1.83,1.95) | 267.78 |
| Bosnia and Herzegovina | 91 (72,110) | 3.21 (2.53,3.9) | 258 (207,309) | 4.07 (3.26,4.87) | 0.22 (0.17,0.26) | 183.51 |
| Botswana | 8 (6,11) | 3.2 (2.25,4.46) | 26 (20,36) | 3.24 (2.42,4.69) | 0.91 (0.8,1.01) | 213.58 |
| Brazil | 2640 (2339,2805) | 5 (4.28,5.38) | 11250 (9412,12337) | 4.84 (4.04,5.31) | 0.4 (0.15,0.64) | 326.19 |
| Brunei Darussalam | 5 (4,6) | 7.15 (5.28,9.27) | 12 (10,13) | 6.22 (5.18,7.43) | -0.15 (-0.24,-0.06) | 131.43 |
| Bulgaria | 393 (346,449) | 5.51 (4.87,6.23) | 902 (786,1026) | 6.17 (5.4,6.98) | 0.22 (-0.04,0.48) | 129.67 |
| Burkina Faso | 82 (48,116) | 3.78 (2.22,5.35) | 270 (181,365) | 5.13 (3.49,6.87) | 0.5 (0.34,0.67) | 229.42 |
| Burundi | 40 (18,74) | 2.73 (1.19,5.17) | 70 (34,135) | 2.74 (1.27,5.53) | 1.35 (1.2,1.49) | 74.25 |
| Cambodia | 74 (55,109) | 3.19 (2.31,4.7) | 279 (217,359) | 4.3 (3.27,5.61) | -0.33 (-0.53,-0.12) | 274.65 |
| Cameroon | 115 (81,146) | 4.99 (3.47,6.41) | 324 (245,422) | 5.21 (4,6.66) | 1.18 (0.93,1.43) | 181.62 |
| Canada | 1498 (1339,1591) | 4.87 (4.33,5.18) | 3777 (3164,4143) | 4.29 (3.62,4.69) | 0.99 (0.86,1.13) | 152.09 |
| Central African Republic | 22 (13,35) | 4.12 (2.33,6.56) | 41 (24,63) | 4.24 (2.44,6.54) | 0.02 (-0.03,0.07) | 85.06 |
| Chad | 53 (31,78) | 2.95 (1.74,4.41) | 113 (77,151) | 3.82 (2.62,5.07) | -0.64 (-0.77,-0.51) | 112.5 |
| Chile | 254 (236,266) | 3.29 (3.01,3.47) | 995 (851,1075) | 3.73 (3.19,4.02) | 0.11 (0.07,0.14) | 291.69 |
| China | 16449 (13240,20521) | 4.93 (3.88,6.17) | 64728 (51765,77729) | 4.33 (3.43,5.23) | 0.84 (0.79,0.89) | 293.51 |
| Colombia | 558 (506,587) | 4.5 (4.03,4.76) | 2331 (1893,2705) | 4.03 (3.31,4.67) | 1.56 (1.04,2.09) | 318.05 |
| Commonwealth of the Bahamas | 7 (6,7) | 5.56 (4.92,6.09) | 19 (16,22) | 6.06 (5.1,7.02) | -0.6 (-0.78,-0.43) | 184.25 |
| Comoros | 4 (2,5) | 3.42 (1.84,5.13) | 10 (6,17) | 3.34 (1.79,5.53) | -0.64 (-0.75,-0.53) | 186.93 |
| Congo | 32 (24,47) | 5.93 (4.41,8.6) | 80 (56,101) | 5.97 (4.33,7.41) | -0.25 (-0.39,-0.11) | 149.44 |
| Cook Islands | 1 (0,1) | 6.82 (5.38,8.29) | 1 (1,2) | 5.99 (4.2,7.74) | -0.09 (-0.19,0.02) | 155.73 |
| Costa Rica | 67 (59,73) | 4.4 (3.89,4.83) | 250 (207,282) | 4.3 (3.59,4.82) | -0.39 (-0.45,-0.33) | 275.12 |
| Croatia | 142 (132,150) | 3.02 (2.79,3.19) | 363 (319,401) | 3.51 (3.09,3.87) | -0.25 (-0.44,-0.06) | 156.22 |
| Cuba | 442 (390,484) | 5.26 (4.58,5.78) | 1090 (943,1220) | 4.87 (4.22,5.46) | 0.25 (0.18,0.31) | 146.32 |
| Cyprus | 59 (40,78) | 14.11 (9.57,19.11) | 123 (102,143) | 8.36 (6.79,9.85) | 0.62 (0.2,1.04) | 109.81 |
| Czech Republic | 505 (473,532) | 4.08 (3.79,4.3) | 1123 (973,1237) | 4.68 (4.06,5.15) | -0.22 (-0.34,-0.09) | 122.1 |
| Democratic People's Republic of Korea | 421 (296,578) | 5.07 (3.43,7.31) | 1167 (886,1665) | 5.09 (3.77,7.73) | -1.76 (-2.04,-1.49) | 177.44 |
| Democratic Republic of the Congo | 281 (167,427) | 3.88 (2.25,5.95) | 854 (527,1390) | 4.45 (2.78,7.34) | 0.68 (0.57,0.79) | 204.19 |
| Denmark | 416 (373,453) | 4.73 (4.22,5.13) | 874 (741,955) | 6.06 (5.17,6.6) | 0.32 (0.16,0.48) | 109.93 |
| Djibouti | 2 (2,3) | 3.73 (2.64,5.16) | 10 (7,15) | 3.79 (2.49,5.46) | 0.45 (0.23,0.67) | 376.72 |
| Dominica | 4 (3,5) | 7.7 (6.4,9.28) | 5 (5,6) | 7.79 (6.56,9.08) | 0.89 (0.44,1.34) | 37.24 |
| Dominican Republic | 134 (110,156) | 6.01 (4.83,6.97) | 464 (361,587) | 4.96 (3.88,6.24) | -0.06 (-0.17,0.04) | 246.42 |
| Ecuador | 193 (171,209) | 5.06 (4.5,5.47) | 553 (464,656) | 4.01 (3.4,4.71) | -0.02 (-0.11,0.06) | 186.27 |
| Egypt | 459 (360,583) | 4.11 (3.16,5.32) | 971 (799,1145) | 3.61 (2.87,4.25) | -0.19 (-0.53,0.14) | 111.62 |
| El Salvador | 133 (110,157) | 4.8 (3.96,5.66) | 402 (307,494) | 5.36 (4.13,6.56) | -0.68 (-0.81,-0.56) | 202.41 |
| Equatorial Guinea | 5 (3,7) | 4.27 (2.66,6.69) | 15 (10,21) | 5.43 (3.59,7.37) | -0.18 (-0.33,-0.03) | 229.69 |
| Eritrea | 15 (9,22) | 3.14 (1.94,4.89) | 49 (26,86) | 3.94 (2.07,7.07) | 0.34 (0.24,0.45) | 224.36 |
| Estonia | 70 (64,77) | 3.95 (3.57,4.34) | 190 (161,212) | 5.33 (4.54,5.96) | 0.83 (0.75,0.91) | 170.38 |
| Ethiopia | 250 (118,380) | 2.58 (1.21,3.96) | 697 (360,1128) | 2.52 (1.29,4.11) | 0.63 (0.57,0.69) | 178.45 |
| Federated States of Micronesia | 2 (2,3) | 8.31 (5.76,11.84) | 3 (2,4) | 8.86 (6.48,11.44) | 0.82 (0.71,0.93) | 38.16 |
| Fiji | 10 (8,12) | 5.29 (4.48,6.39) | 26 (20,33) | 6.7 (5.37,8) | 0.67 (0.3,1.03) | 162.89 |
| Finland | 545 (466,595) | 7.77 (6.59,8.51) | 794 (645,877) | 4.67 (3.87,5.13) | -0.3 (-0.43,-0.17) | 45.66 |
| France | 4923 (4382,5224) | 5.51 (4.88,5.85) | 9758 (8012,10779) | 4.53 (3.78,4.99) | 0.52 (0.31,0.74) | 98.22 |
| Gabon | 25 (19,35) | 6.35 (4.65,8.87) | 41 (31,50) | 6.94 (5.29,8.37) | -1.85 (-2.14,-1.57) | 66 |
| Georgia | 168 (126,202) | 3.26 (2.37,3.99) | 341 (293,380) | 5.05 (4.37,5.62) | -0.55 (-0.61,-0.48) | 102.89 |
| Germany | 8302 (7144,9077) | 6.39 (5.46,6.99) | 20539 (16509,22874) | 7.81 (6.35,8.66) | 0.13 (0.02,0.24) | 147.4 |
| Ghana | 128 (101,155) | 4.33 (3.42,5.3) | 346 (267,424) | 4.26 (3.27,5.27) | 0.92 (0.88,0.95) | 170.96 |
| Greece | 590 (529,621) | 4.51 (4,4.77) | 1527 (1272,1673) | 4.25 (3.6,4.62) | 1.43 (0.52,2.34) | 158.95 |
| Greenland | 2 (1,2) | 9.44 (7.45,10.9) | 3 (2,4) | 7.37 (5.55,9.35) | 1.17 (0.96,1.37) | 83.71 |
| Grenada | 5 (4,5) | 5.35 (4.48,6.2) | 5 (5,6) | 7.08 (6.12,7.69) | -0.4 (-0.55,-0.25) | 16.13 |
| Guam | 2 (2,3) | 7.3 (6.23,8.47) | 5 (4,6) | 2.08 (1.64,2.41) | -0.3 (-0.36,-0.24) | 105.11 |
| Guatemala | 84 (78,92) | 5.44 (4.77,6.18) | 358 (309,404) | 4.32 (3.74,4.84) | -0.51 (-0.64,-0.38) | 325.3 |
| Guinea | 75 (45,109) | 3.53 (2.08,5.15) | 153 (110,198) | 4.33 (3.15,5.56) | 0.82 (0.47,1.17) | 104.62 |
| Guinea-Bissau | 8 (6,11) | 4.3 (3.11,6.14) | 14 (11,19) | 5.1 (3.79,6.68) | -3 (-3.56,-2.45) | 84.16 |
| Guyana | 12 (11,13) | 4.65 (4.16,5.09) | 24 (20,29) | 5.58 (4.58,6.68) | -0.71 (-0.88,-0.54) | 96.34 |
| Haiti | 97 (64,153) | 5.81 (3.86,9.01) | 219 (147,326) | 5.63 (3.8,8.29) | 0.71 (0.65,0.76) | 125.36 |
| Honduras | 56 (40,75) | 4.29 (3.05,6.07) | 302 (230,372) | 7.51 (5.71,9.15) | 0.61 (0.56,0.65) | 442.37 |
| Hungary | 528 (494,563) | 4.34 (4.02,4.63) | 796 (678,894) | 3.55 (3.03,3.99) | 0.61 (0.24,0.99) | 50.74 |
| Iceland | 18 (15,19) | 5.62 (4.88,6.04) | 51 (40,58) | 6.84 (5.49,7.68) | -0.04 (-0.09,0.02) | 187.05 |
| India | 5413 (3291,8480) | 2.15 (1.32,3.41) | 27888 (20426,34928) | 3.44 (2.5,4.32) | 1.8 (1.47,2.14) | 415.18 |
| Indonesia | 1899 (1376,2468) | 3.6 (2.54,4.93) | 6987 (5445,8421) | 6.36 (4.84,7.8) | -0.38 (-0.52,-0.25) | 267.9 |
| Iraq | 226 (161,304) | 3.25 (2.31,4.38) | 647 (481,826) | 4.85 (3.51,6.19) | 1.05 (0.86,1.23) | 186.15 |
| Ireland | 220 (200,231) | 6.07 (5.45,6.42) | 456 (371,510) | 5.2 (4.23,5.8) | 1.84 (1.53,2.16) | 107.92 |
| Islamic Republic of Iran | 411 (306,510) | 3.32 (2.42,4.15) | 1949 (1463,2233) | 3.31 (2.47,3.8) | 1.84 (1.72,1.96) | 374.6 |
| Israel | 256 (228,273) | 6.31 (5.5,6.76) | 714 (580,794) | 4.84 (3.98,5.35) | -0.07 (-0.15,0.02) | 178.81 |
| Italy | 3323 (2852,3560) | 4.09 (3.47,4.41) | 9825 (7691,11040) | 4.52 (3.6,5.05) | 0.79 (0.58,1.01) | 195.66 |
| Jamaica | 95 (85,105) | 5.17 (4.6,5.7) | 206 (164,251) | 5.46 (4.36,6.68) | -0.25 (-0.49,0) | 115.83 |
| Japan | 4429 (3883,4699) | 3.04 (2.63,3.25) | 13837 (10679,15687) | 2.33 (1.86,2.58) | -0.66 (-0.85,-0.48) | 212.44 |
| Jordan | 23 (19,28) | 3.14 (2.54,3.78) | 103 (80,125) | 2.72 (2.12,3.29) | 0.76 (0.54,0.97) | 339.28 |
| Kazakhstan | 213 (172,265) | 2.2 (1.75,2.79) | 350 (305,395) | 2.97 (2.57,3.34) | 0.19 (-0.01,0.4) | 64.47 |
| Kenya | 122 (74,167) | 2.43 (1.48,3.32) | 407 (264,581) | 3.39 (2.18,4.95) | -1.58 (-1.98,-1.17) | 234.18 |
| Kingdom of Eswatini | 6 (4,7) | 3.63 (2.61,4.89) | 11 (7,14) | 3.61 (2.59,4.61) | -0.56 (-0.79,-0.33) | 89.79 |
| Kiribati | 1 (1,1) | 3.19 (2.42,4.37) | 1 (1,2) | 3.86 (3.02,5.05) | 0.49 (0.24,0.75) | 112.81 |
| Kuwait | 9 (7,10) | 2.59 (2.08,2.95) | 60 (48,72) | 3 (2.4,3.57) | 1.22 (1.1,1.34) | 605.61 |
| Kyrgyzstan | 46 (38,56) | 2.01 (1.65,2.47) | 73 (61,84) | 2.17 (1.82,2.49) | 0.55 (0.45,0.65) | 58.08 |
| Lao People's Democratic Republic | 41 (27,60) | 4.19 (2.82,6.25) | 130 (102,169) | 5.14 (4.04,6.72) | 0.82 (0.26,1.39) | 217.5 |
| Latvia | 122 (112,129) | 3.74 (3.44,3.97) | 226 (194,250) | 4.49 (3.88,4.98) | 0.37 (0.12,0.63) | 85.42 |
| Lebanon | 73 (41,113) | 4.96 (2.67,7.76) | 239 (193,308) | 3.41 (2.75,4.35) | 0.6 (0.57,0.64) | 228.47 |
| Lesotho | 14 (10,20) | 2.42 (1.67,3.51) | 22 (16,29) | 3.53 (2.63,4.59) | 0.8 (0.59,1.02) | 59.23 |
| Liberia | 26 (17,33) | 4.12 (2.69,5.34) | 50 (36,67) | 4.52 (3.28,5.97) | -1.46 (-1.61,-1.32) | 93.84 |
| Libya | 38 (27,53) | 2.48 (1.79,3.43) | 111 (69,163) | 3.07 (1.9,4.49) | 2.28 (1.85,2.71) | 192.8 |
| Lithuania | 154 (138,170) | 3.67 (3.3,4.04) | 328 (288,363) | 4.52 (3.98,5.01) | 0.35 (0.29,0.4) | 113.18 |
| Luxembourg | 33 (31,35) | 6.64 (6.11,7.02) | 83 (70,92) | 6.32 (5.38,7.03) | 1.43 (1.15,1.71) | 151.14 |
| Madagascar | 153 (98,205) | 4.9 (3.08,6.61) | 271 (177,388) | 5.12 (3.35,7.35) | 0.73 (0.62,0.84) | 76.7 |
| Malawi | 48 (25,70) | 2.42 (1.22,3.61) | 122 (77,179) | 2.94 (1.86,4.29) | 0.24 (0.07,0.41) | 156.08 |
| Malaysia | 237 (193,289) | 3.22 (2.6,3.94) | 931 (786,1069) | 4.94 (4.12,5.74) | 0.14 (0.02,0.26) | 293.05 |
| Maldives | 1 (1,2) | 4.05 (2.57,5.55) | 10 (7,12) | 4.06 (2.99,5.05) | 0.58 (0.48,0.69) | 589.5 |
| Mali | 52 (34,75) | 2.84 (1.81,4.14) | 128 (90,170) | 2.95 (2.12,3.84) | 1.54 (1.14,1.94) | 147.62 |
| Malta | 19 (17,20) | 5.28 (4.67,5.65) | 58 (47,66) | 4.88 (3.97,5.49) | -0.22 (-0.37,-0.06) | 208.2 |
| Marshall Islands | 1 (0,1) | 6.9 (5.34,9.13) | 1 (1,2) | 7.35 (5.53,9.4) | 0.24 (0.11,0.37) | 94.15 |
| Mauritania | 28 (22,35) | 4.85 (3.79,5.96) | 73 (54,96) | 5.36 (4.02,7.01) | -0.02 (-0.18,0.14) | 158.45 |
| Mauritius | 23 (21,24) | 5.36 (4.79,5.74) | 76 (66,82) | 4.84 (4.24,5.26) | 0.1 (0.03,0.16) | 232.25 |
| Mexico | 1500 (1417,1547) | 5.69 (5.32,5.89) | 5147 (4611,5659) | 4.86 (4.33,5.33) | 0.12 (0,0.24) | 243.18 |
| Mongolia | 15 (12,19) | 2 (1.54,2.61) | 26 (20,33) | 1.91 (1.49,2.44) | -0.42 (-0.58,-0.27) | 73.19 |
| Montenegro | 61 (52,78) | 11.32 (9.48,14.4) | 133 (107,163) | 17.26 (13.44,21.44) | -0.44 (-0.53,-0.36) | 116.67 |
| Morocco | 304 (183,417) | 3.04 (1.79,4.21) | 991 (711,1280) | 4.28 (3.05,5.49) | 0.16 (0.14,0.18) | 226.18 |
| Mozambique | 104 (56,152) | 3.3 (1.83,4.93) | 252 (156,395) | 4.34 (2.74,6.93) | 0.11 (-0.1,0.31) | 143.59 |
| Myanmar | 504 (350,722) | 4.16 (2.93,5.94) | 1630 (1236,2084) | 5.15 (3.9,6.6) | -0.59 (-0.74,-0.43) | 223.52 |
| Namibia | 10 (7,13) | 3.08 (2.29,4.14) | 29 (21,40) | 3.77 (2.68,5.09) | 1.7 (1.23,2.18) | 197.66 |
| Nepal | 116 (63,184) | 2.31 (1.25,3.65) | 589 (401,829) | 4.05 (2.76,5.8) | 1.2 (1.05,1.35) | 406.9 |
| Netherlands | 1399 (1213,1507) | 6.99 (6.03,7.56) | 2501 (2084,2738) | 6.01 (5.03,6.57) | 1.25 (1.12,1.39) | 78.85 |
| New Zealand | 281 (246,300) | 8.01 (6.94,8.6) | 729 (593,805) | 7.52 (6.14,8.3) | 0.46 (0.36,0.57) | 159.74 |
| Nicaragua | 39 (32,46) | 3.45 (2.84,4.13) | 137 (111,165) | 3.7 (3,4.48) | 0.66 (0.46,0.85) | 253.36 |
| Niger | 32 (17,49) | 2.77 (1.43,4.25) | 114 (57,174) | 3.07 (1.61,4.71) | 0.82 (0.79,0.84) | 251.2 |
| Nigeria | 1258 (955,1655) | 5.1 (3.87,6.71) | 2185 (1644,2671) | 4.48 (3.44,5.46) | 2 (1.72,2.28) | 73.68 |
| Northern Mariana Islands | 0 (0,1) | 6.73 (5.51,8.36) | 2 (2,3) | 7.7 (6.44,8.85) | -0.72 (-0.86,-0.59) | 353.09 |
| Norway | 491 (431,522) | 6.27 (5.51,6.68) | 778 (629,857) | 5.94 (4.86,6.51) | 0.04 (-0.1,0.17) | 58.54 |
| Oman | 17 (12,23) | 4.03 (2.91,5.59) | 45 (36,55) | 4.73 (3.62,5.93) | 0.57 (0.31,0.82) | 173.11 |
| Pakistan | 1035 (660,1504) | 2.8 (1.81,4.06) | 3115 (2311,4332) | 4.58 (3.33,6.38) | 0.36 (0.3,0.42) | 200.89 |
| Palestine | 29 (21,37) | 5.11 (3.74,6.68) | 68 (55,82) | 5.37 (4.31,6.66) | -0.74 (-0.89,-0.59) | 139.1 |
| Panama | 53 (47,57) | 4.23 (3.73,4.57) | 221 (170,263) | 4.59 (3.55,5.46) | -0.07 (-0.11,-0.03) | 319.59 |
| Papua New Guinea | 32 (19,46) | 3.46 (2.1,4.84) | 108 (67,154) | 3.89 (2.43,5.52) | 1.47 (0.87,2.08) | 234.55 |
| Paraguay | 88 (71,102) | 4.78 (3.89,5.6) | 290 (218,360) | 5.73 (4.31,7.1) | 0.36 (0.06,0.65) | 231.31 |
| Peru | 435 (364,513) | 4.33 (3.61,5.09) | 1169 (896,1501) | 3.47 (2.66,4.45) | -0.25 (-0.51,0.01) | 168.55 |
| Philippines | 691 (595,803) | 5.26 (4.52,6.12) | 2445 (2054,2912) | 4.82 (4.02,5.81) | 1.06 (0.62,1.5) | 253.92 |
| Plurinational State of Bolivia | 112 (80,158) | 5.49 (4.04,7.63) | 358 (258,486) | 5.65 (4.13,7.54) | 1.41 (1.14,1.67) | 219.59 |
| Poland | 2503 (2303,2671) | 6.8 (6.18,7.24) | 4021 (3480,4421) | 4.96 (4.31,5.45) | 0.08 (-0.02,0.18) | 60.65 |
| Portugal | 507 (462,531) | 4.69 (4.21,4.93) | 1173 (969,1288) | 3.46 (2.9,3.78) | 0 (-0.17,0.18) | 131.31 |
| Principality of Monaco | 4 (3,5) | 4.34 (3.2,5.36) | 6 (4,7) | 4.35 (3.29,5.49) | 0.34 (0.23,0.45) | 57.67 |
| Puerto Rico | 170 (156,180) | 5.97 (5.48,6.31) | 390 (313,452) | 3.82 (3.11,4.43) | 0.38 (0.35,0.41) | 128.79 |
| Qatar | 2 (1,3) | 7.37 (3.17,10.02) | 10 (5,15) | 4.06 (1.45,6.04) | 0.9 (0.74,1.06) | 331.25 |
| Republic of Cabo Verde | 8 (6,11) | 3.62 (2.65,4.73) | 22 (16,28) | 5.43 (3.85,6.88) | -1.03 (-1.34,-0.73) | 165.62 |
| Republic of C么te d'Ivoire | 65 (49,83) | 4.26 (3.16,5.42) | 244 (188,307) | 4.71 (3.72,5.83) | 0.16 (0,0.32) | 273.43 |
| Republic of Korea | 553 (422,873) | 3.38 (2.55,5.22) | 3165 (2356,3785) | 3.5 (2.6,4.17) | -0.67 (-1.18,-0.16) | 472.23 |
| Republic of Moldova | 108 (99,116) | 3.96 (3.65,4.24) | 215 (189,238) | 3.5 (3.09,3.88) | -1.17 (-1.36,-0.99) | 99.56 |
| Republic of Nauru | 0 (0,0) | 8.16 (6.34,10.16) | 0 (0,0) | 10.35 (6.38,18.44) | -1.6 (-1.72,-1.47) | 61.92 |
| Republic of Niue | 0 (0,0) | 7.28 (5.81,8.89) | 0 (0,0) | 7.39 (5.57,8.79) | -2.71 (-3.39,-2.04) | -24.31 |
| Republic of Palau | 0 (0,0) | 4.84 (3.86,6.01) | 1 (0,1) | 4.69 (3.77,5.59) | 0.61 (0.41,0.81) | 90.92 |
| Republic of San Marino | 2 (2,3) | 5.93 (4.79,7) | 4 (2,5) | 3.03 (2.03,4.31) | -0.63 (-0.8,-0.46) | 65.3 |
| Republic of the Gambia | 6 (4,9) | 3.86 (2.69,5.24) | 29 (22,36) | 5.11 (3.91,6.43) | -0.56 (-0.74,-0.39) | 354.34 |
| Romania | 674 (625,726) | 3.58 (3.33,3.83) | 1429 (1263,1586) | 3.35 (2.97,3.72) | 0.38 (0.25,0.51) | 112 |
| Russian Federation | 5109 (4515,5816) | 3.92 (3.42,4.52) | 10825 (9597,11704) | 4.44 (3.92,4.8) | -1.31 (-1.62,-1) | 111.89 |
| Rwanda | 56 (27,84) | 3.74 (1.71,5.7) | 102 (42,179) | 3.03 (1.24,5.39) | -0.05 (-0.26,0.17) | 80.81 |
| Saint Kitts and Nevis | 2 (2,3) | 8.38 (7.51,9.3) | 3 (3,3) | 7.65 (6.77,8.39) | -2 (-2.33,-1.68) | 28.6 |
| Saint Lucia | 6 (5,6) | 10.41 (9.62,11.35) | 16 (13,19) | 7.2 (5.98,8.44) | -0.18 (-0.39,0.03) | 168.16 |
| Saint Vincent and the Grenadines | 4 (4,5) | 7.93 (7.37,8.39) | 8 (7,9) | 7.43 (6.63,8.15) | -0.05 (-0.14,0.03) | 86.03 |
| Samoa | 4 (3,5) | 6.77 (4.91,8.9) | 7 (5,9) | 6.72 (5.11,8.46) | -1.3 (-1.69,-0.91) | 85.5 |
| Sao Tome and Principe | 2 (1,2) | 3.49 (2.42,5.7) | 3 (2,4) | 4.94 (3.79,5.87) | 1.42 (1.28,1.55) | 96.4 |
| Saudi Arabia | 102 (78,142) | 2.97 (2.27,4.12) | 249 (197,305) | 3.35 (2.67,4.11) | 0.3 (0.2,0.4) | 143.13 |
| Senegal | 72 (49,97) | 3.89 (2.64,5.32) | 232 (173,291) | 5.03 (3.76,6.29) | 0.67 (0.6,0.75) | 222.86 |
| Serbia | 427 (363,489) | 6.22 (5.28,7.24) | 810 (665,962) | 4.63 (3.83,5.5) | -1.49 (-1.84,-1.13) | 89.72 |
| Seychelles | 3 (2,3) | 5.03 (4.27,6.17) | 5 (4,6) | 5.46 (4.33,6.73) | 0.47 (0.29,0.66) | 71.5 |
| Sierra Leone | 45 (30,62) | 3.46 (2.27,4.78) | 82 (61,106) | 3.86 (2.96,4.89) | 0.38 (0.33,0.43) | 83.28 |
| Singapore | 32 (29,33) | 1.94 (1.78,2.04) | 99 (84,108) | 1.22 (1.03,1.33) | -1.82 (-2.05,-1.59) | 213.91 |
| Slovakia | 305 (263,366) | 5.75 (4.94,6.89) | 562 (463,658) | 5.99 (4.9,7.02) | 0.39 (0.28,0.5) | 84.61 |
| Slovenia | 83 (76,89) | 3.54 (3.22,3.83) | 214 (175,242) | 3.67 (3.01,4.15) | 0.46 (0.03,0.88) | 157.91 |
| Socialist Republic of Viet Nam | 1219 (961,1630) | 4.14 (3.22,5.56) | 4015 (2990,5030) | 5.83 (4.35,7.36) | 0.58 (0.56,0.6) | 229.32 |
| Solomon Islands | 3 (1,4) | 4.09 (2.11,6.13) | 9 (5,13) | 4.92 (2.65,8.18) | -0.56 (-0.64,-0.48) | 249.14 |
| Somalia | 32 (15,51) | 2.79 (1.29,4.5) | 61 (26,111) | 2.23 (1.02,3.98) | 1.16 (0.82,1.51) | 91.81 |
| South Africa | 411 (328,515) | 2.77 (2.2,3.48) | 1208 (1035,1329) | 4.04 (3.43,4.51) | -0.4 (-0.52,-0.28) | 194.13 |
| South Sudan | 54 (25,82) | 3.32 (1.57,4.96) | 63 (31,99) | 2.97 (1.47,4.74) | -0.35 (-0.44,-0.26) | 15.63 |
| Spain | 2611 (2310,2779) | 5.21 (4.56,5.56) | 6767 (5396,7550) | 4.54 (3.69,5.04) | 1.03 (0.74,1.31) | 159.17 |
| Sri Lanka | 209 (180,246) | 3.64 (3.07,4.33) | 777 (556,1029) | 4.05 (2.89,5.35) | 0.32 (0.21,0.42) | 272.55 |
| Sudan | 155 (98,232) | 2.87 (1.76,4.2) | 394 (287,519) | 3.24 (2.32,4.25) | 0.11 (-0.04,0.26) | 154.29 |
| Suriname | 12 (10,14) | 5.52 (4.6,6.35) | 29 (21,37) | 5.28 (3.89,6.83) | 2.57 (2.28,2.87) | 141.74 |
| Sweden | 839 (737,898) | 4.9 (4.29,5.25) | 2851 (2296,3193) | 9.47 (7.71,10.59) | 0.62 (0.5,0.75) | 239.71 |
| Switzerland | 317 (271,346) | 2.74 (2.35,3.01) | 773 (609,867) | 3 (2.39,3.34) | -0.11 (-0.31,0.09) | 143.96 |
| Syrian Arab Republic | 153 (109,201) | 4.26 (3.02,5.62) | 318 (210,419) | 4.57 (2.89,5.92) | -1.36 (-1.67,-1.04) | 108.68 |
| Taiwan (Province of China) | 403 (369,425) | 5.23 (4.64,5.59) | 1771 (1474,1977) | 3.71 (3.12,4.12) | -0.61 (-0.96,-0.26) | 339.89 |
| Tajikistan | 28 (20,41) | 1.26 (0.9,1.9) | 39 (32,48) | 1.15 (0.92,1.44) | -1.35 (-1.56,-1.15) | 42.23 |
| Thailand | 1206 (930,1572) | 5.81 (4.39,7.57) | 5064 (3744,6440) | 4.56 (3.39,5.83) | 0.89 (0.77,1.01) | 319.94 |
| The former Yugoslav Republic of Macedonia | 64 (54,77) | 4.41 (3.68,5.34) | 149 (110,214) | 7.45 (5.8,9.5) | 0.6 (0.57,0.63) | 132.68 |
| Timor-Leste | 5 (3,7) | 3.56 (2.28,5.09) | 22 (17,28) | 4.54 (3.47,5.8) | -0.06 (-0.08,-0.03) | 352.72 |
| Togo | 24 (17,31) | 3.83 (2.84,5.08) | 77 (57,98) | 4.7 (3.6,5.93) | 0.48 (0.36,0.6) | 226.76 |
| Tokelau | 0 (0,0) | 7.36 (5.74,9.22) | 0 (0,0) | 7.16 (4.95,10.39) | -0.79 (-0.92,-0.66) | 34.53 |
| Tonga | 2 (1,2) | 4.41 (3,6.32) | 4 (3,5) | 5.01 (3.51,6.87) | 0.6 (0.52,0.68) | 120.55 |
| Trinidad and Tobago | 38 (35,40) | 7.53 (6.81,8.15) | 99 (77,119) | 5.64 (4.41,6.79) | 0.75 (0.17,1.33) | 160.91 |
| Tunisia | 94 (69,117) | 3.62 (2.6,4.59) | 463 (305,649) | 4.61 (2.98,6.69) | 0.5 (0.29,0.72) | 394.42 |
| Turkey | 836 (659,1100) | 3.59 (2.79,4.75) | 2923 (2332,3533) | 3.88 (3.05,4.69) | 0.02 (-0.03,0.06) | 249.83 |
| Turkmenistan | 29 (24,34) | 2.19 (1.83,2.65) | 86 (68,112) | 2.89 (2.27,3.76) | 0.15 (0.02,0.28) | 200.69 |
| Tuvalu | 0 (0,0) | 6.98 (5.26,9.23) | 0 (0,1) | 7.14 (5.55,8.84) | -0.21 (-0.44,0.03) | 95.92 |
| Uganda | 103 (44,165) | 2.69 (1.13,4.35) | 251 (126,429) | 2.91 (1.43,4.96) | 2.97 (1.9,4.04) | 144.2 |
| Ukraine | 2156 (1971,2362) | 3.85 (3.47,4.24) | 3236 (2489,4051) | 4.1 (3.16,5.13) | 0.3 (0.17,0.43) | 50.11 |
| United Arab Emirates | 7 (5,10) | 3.62 (2.64,5.17) | 30 (24,37) | 3.82 (2.51,4.93) | -0.16 (-0.36,0.04) | 345 |
| United Kingdom of Great Britain and Northern Ireland | 5083 (4544,5338) | 5.47 (4.84,5.77) | 9412 (7868,10176) | 5.71 (4.8,6.15) | 0.95 (0.88,1.02) | 85.17 |
| United Republic of Tanzania | 216 (115,327) | 3.61 (1.91,5.49) | 580 (329,891) | 3.6 (2.05,5.39) | -1.04 (-1.26,-0.82) | 168.37 |
| United States of America | 13044 (11221,13964) | 3.88 (3.33,4.16) | 35285 (28993,38677) | 5.26 (4.36,5.74) | 0.71 (0.58,0.84) | 170.51 |
| United States Virgin Islands | 4 (3,4) | 7.66 (6.41,8.8) | 8 (6,10) | 5.15 (3.97,6.7) | 1.26 (1,1.52) | 118.98 |
| Uruguay | 117 (105,126) | 3.23 (2.87,3.49) | 277 (235,301) | 3.78 (3.24,4.1) | 0.35 (0.29,0.41) | 137.32 |
| Uzbekistan | 97 (57,158) | 1.03 (0.6,1.69) | 240 (204,278) | 1.39 (1.18,1.6) | -0.14 (-0.29,0.01) | 147.69 |
| Vanuatu | 1 (1,2) | 4.62 (3.07,6.47) | 5 (3,6) | 5.14 (3.8,6.54) | 1.06 (0.97,1.15) | 226.34 |
| Yemen | 76 (44,121) | 3.08 (1.79,4.86) | 291 (198,404) | 3.74 (2.57,5.12) | 0.6 (0.55,0.65) | 285.09 |
| Zambia | 64 (44,81) | 4.11 (2.84,5.3) | 218 (129,366) | 5.71 (3.59,9.09) | 1.25 (0.95,1.55) | 242.82 |
| Zimbabwe | 63 (50,76) | 3.08 (2.34,3.75) | 118 (80,149) | 3.39 (2.23,4.25) | 0.81 (0.5,1.13) | 86.79 |

AF/AFL, Atrial fibrillation (AF)/atrial flutter (AFL); CI, confidence interval; EAPC, estimated annual percentage change; UI, uncertainty interval.

Supplementary Table 7 DALYs of AF/AFL Between 1990 and 2021 in 204 countries

| **Location** | **Incidence in 1990** | | **Incidence in 2021** | | **1990-2021 EAPC** | **1990-2021 change** |
| --- | --- | --- | --- | --- | --- | --- |
|  | **Number(95%UI)** | **Rate(95%UI)** | **Number(95%UI)** | **Rate(95%UI)** | **EAPC_95%CI** |  |
| Afghanistan | 3394 (2188,4977) | 63.05 (40.48,92.63) | 5074 (3663,6761) | 72.08 (51.55,95.15) | 0.46 (0.43,0.48) | 49.5 |
| Albania | 1700 (1354,2122) | 97.6 (78.23,120.32) | 4399 (3431,5468) | 103.15 (81.18,126.41) | 0.39 (0.29,0.48) | 158.81 |
| Algeria | 6619 (5035,8537) | 90.02 (68.59,112.91) | 23845 (18698,29189) | 95.93 (76.67,116.45) | 0.63 (0.48,0.77) | 260.24 |
| American Samoa | 23 (18,27) | 133.26 (107.73,163.29) | 58 (45,71) | 143.68 (111.47,176.76) | 0.34 (0.31,0.37) | 156.45 |
| Andorra | 64 (49,85) | 125.54 (95.34,164.86) | 170 (130,217) | 102.38 (77.85,131.72) | -0.6 (-0.69,-0.52) | 164.81 |
| Angola | 2447 (1758,3251) | 89.65 (65.44,120.75) | 8368 (6207,11093) | 102.72 (76.04,135.77) | 0.37 (0.32,0.42) | 242 |
| Antigua and Barbuda | 80 (70,95) | 139.73 (120.93,164.23) | 121 (101,147) | 132.09 (111.79,158.06) | -0.17 (-0.31,-0.04) | 51.01 |
| Argentina | 24020 (19576,29910) | 82.39 (67.9,101.12) | 40198 (34929,46587) | 69.17 (59.96,80.23) | -0.1 (-0.31,0.12) | 67.35 |
| Armenia | 1451 (1074,1905) | 60.37 (45.72,79.07) | 3355 (2607,4231) | 76.87 (59.76,96.05) | 0.98 (0.81,1.15) | 131.24 |
| Australia | 28469 (24181,33071) | 152.15 (129.99,175.93) | 73571 (60143,89381) | 146.76 (119.81,179.53) | 0 (-0.09,0.1) | 158.42 |
| Austria | 14654 (12661,16940) | 119.39 (103.2,137.72) | 37096 (30927,43902) | 175.92 (146.38,208.58) | 1.46 (1.25,1.68) | 153.14 |
| Azerbaijan | 2729 (1994,3648) | 62.51 (46.28,83.71) | 5902 (4361,7951) | 67.92 (51.38,90.14) | 0.43 (0.35,0.51) | 116.23 |
| Bahrain | 101 (65,128) | 113.06 (66.97,143.09) | 442 (316,569) | 94.91 (57.64,122.79) | 0.02 (-0.1,0.13) | 336.23 |
| Bangladesh | 29954 (20406,40049) | 78.66 (52.88,105.79) | 109146 (82747,147679) | 97.3 (74.5,129.97) | -0.72 (-1,-0.45) | 264.38 |
| Barbados | 376 (312,456) | 123.26 (102.83,148.15) | 641 (513,774) | 123.06 (98.96,148.54) | 0.64 (0.46,0.82) | 70.53 |
| Belarus | 11328 (9217,14087) | 91.19 (74.49,113.12) | 16740 (13493,20892) | 101.79 (82.13,126.55) | 0.03 (-0.06,0.12) | 47.77 |
| Belgium | 17560 (14240,21830) | 111.27 (90.86,138.05) | 27975 (22943,33735) | 103.49 (84.22,126.61) | 0.24 (0.17,0.31) | 59.32 |
| Belize | 96 (76,122) | 105.76 (83.07,134.19) | 297 (239,365) | 113.27 (91.6,138.44) | -0.11 (-0.24,0.03) | 209.24 |
| Benin | 1095 (824,1396) | 66.58 (50.37,84.27) | 2817 (2218,3600) | 73.63 (57.93,92.8) | 0.11 (-0.13,0.35) | 157.22 |
| Bermuda | 76 (60,92) | 136.3 (109.71,164.1) | 160 (130,197) | 105.58 (84.94,130.79) | 0.36 (0.32,0.39) | 110.73 |
| Bhutan | 123 (86,167) | 71.66 (49.36,97.91) | 503 (379,649) | 93.85 (70.8,121.02) | -0.93 (-0.99,-0.87) | 309.97 |
| Bolivarian Republic of Venezuela | 9691 (7702,11944) | 116.92 (94.53,143.09) | 31981 (25013,39311) | 114.29 (89.6,140.04) | 0.95 (0.92,0.98) | 230 |
| Bosnia and Herzegovina | 3085 (2409,3907) | 89.03 (70.69,112.39) | 6410 (5131,7828) | 99.83 (80.18,121.35) | 0.1 (0.07,0.12) | 107.76 |
| Botswana | 310 (233,407) | 76.84 (58.18,100.28) | 862 (665,1099) | 77.73 (60.71,99.9) | 0.46 (0.4,0.52) | 177.89 |
| Brazil | 88346 (69433,110571) | 122.91 (99.74,151.06) | 297032 (239336,367908) | 122.58 (99.23,151.64) | 0.18 (0.05,0.32) | 236.21 |
| Brunei Darussalam | 124 (102,149) | 144.66 (117.27,173.57) | 328 (271,388) | 122.03 (102.46,143.98) | -0.05 (-0.1,-0.01) | 163.88 |
| Bulgaria | 12305 (10093,15114) | 118.85 (99.82,142.79) | 19898 (16801,23531) | 132.45 (112.78,156.09) | -0.2 (-0.36,-0.05) | 61.7 |
| Burkina Faso | 2340 (1612,3065) | 75.92 (51.64,100.43) | 6312 (4577,8276) | 93.72 (69,121.53) | 0.39 (0.29,0.48) | 169.72 |
| Burundi | 1371 (900,2021) | 71.61 (46.75,109.02) | 2555 (1694,3792) | 69.88 (45.58,107.87) | 0.91 (0.82,1) | 86.43 |
| Cambodia | 2927 (2223,3785) | 86.33 (66.07,113.06) | 9138 (7031,11349) | 98.48 (76.53,122.26) | -0.26 (-0.38,-0.15) | 212.18 |
| Cameroon | 2795 (2110,3447) | 90.76 (68.25,111.88) | 8308 (6392,10485) | 96.96 (75.89,120.43) | 0.69 (0.54,0.85) | 197.27 |
| Canada | 49511 (38763,61653) | 152.55 (120.12,189.47) | 99213 (77674,125422) | 125.65 (97.53,160.69) | 0.42 (0.35,0.49) | 100.39 |
| Central African Republic | 741 (498,1034) | 93.67 (63.8,132.54) | 1396 (964,1962) | 94.52 (64.64,133.89) | 0.13 (0.08,0.17) | 88.35 |
| Chad | 1481 (1037,2001) | 64.7 (45.18,87.18) | 3120 (2274,4079) | 76.68 (56.2,97.97) | -0.66 (-0.75,-0.56) | 110.64 |
| Chile | 7594 (6160,9340) | 84.57 (69.33,103.12) | 22386 (18415,26886) | 85.2 (70.05,102.61) | 0.02 (0,0.04) | 194.78 |
| China | 508610 (395853,638618) | 93.28 (75.14,115.5) | 1653117 (1303681,2056459) | 89.76 (72.13,109.67) | 0.55 (0.51,0.58) | 225.03 |
| Colombia | 15779 (12835,19518) | 107.08 (88.03,131.81) | 57513 (46273,72041) | 102.82 (82.58,129.17) | 0.6 (0.29,0.91) | 264.49 |
| Commonwealth of the Bahamas | 177 (148,215) | 130.83 (110.46,157.54) | 469 (380,561) | 132.64 (108.37,157.69) | -0.21 (-0.31,-0.11) | 164.54 |
| Comoros | 118 (79,159) | 79.03 (53.09,107.56) | 303 (209,425) | 76.29 (51.65,110.47) | -0.26 (-0.32,-0.21) | 156.84 |
| Congo | 920 (695,1253) | 120.05 (93.69,164.53) | 2231 (1708,2802) | 118.63 (92.06,145.96) | -0.23 (-0.32,-0.14) | 142.4 |
| Cook Islands | 15 (12,18) | 143.94 (115.36,176.95) | 33 (25,43) | 133.97 (101.01,170.52) | -0.14 (-0.22,-0.06) | 126.21 |
| Costa Rica | 1827 (1467,2302) | 112.76 (90.97,140.74) | 6111 (4936,7505) | 109.7 (88.42,135.24) | -0.19 (-0.24,-0.15) | 234.52 |
| Croatia | 3763 (3147,4496) | 69.49 (58.62,82.02) | 7519 (6521,8661) | 76.37 (65.94,88.41) | -0.19 (-0.27,-0.11) | 99.83 |
| Cuba | 11364 (9070,13947) | 118.49 (95.6,143.76) | 23660 (19350,29217) | 113.08 (92.16,140.13) | 0.2 (0.14,0.25) | 108.21 |
| Cyprus | 1241 (932,1550) | 217.88 (160.27,274.78) | 2333 (1967,2795) | 129.79 (108.76,154.1) | 0.63 (0.34,0.91) | 87.89 |
| Czech Republic | 14372 (11698,17635) | 105.91 (86.52,128.9) | 31633 (25775,37952) | 135.11 (109.98,161.89) | -0.13 (-0.19,-0.08) | 120.1 |
| Democratic People's Republic of Korea | 12101 (9159,15414) | 103.92 (79.23,132.66) | 29340 (22444,38029) | 103.98 (80.31,138.02) | -1.71 (-1.91,-1.5) | 142.46 |
| Democratic Republic of the Congo | 9495 (6434,13068) | 87.89 (60.01,121.48) | 24604 (16933,35653) | 94.61 (66.05,140.02) | 0.99 (0.76,1.22) | 159.12 |
| Denmark | 10814 (8775,13295) | 125.94 (101.96,153.71) | 18911 (15746,22897) | 145.11 (120.42,176.86) | 0.17 (0.08,0.27) | 74.87 |
| Djibouti | 76 (57,100) | 81.99 (62.73,107.18) | 368 (267,489) | 83.68 (61.71,110.9) | 0.22 (0.09,0.36) | 387.02 |
| Dominica | 84 (68,102) | 147.85 (120.39,180) | 115 (95,138) | 150.75 (124.68,179.75) | 0.31 (0,0.61) | 38.02 |
| Dominican Republic | 3597 (2866,4515) | 122.8 (100.09,151.2) | 11054 (8859,13676) | 114.8 (92.06,141.9) | 0 (-0.06,0.05) | 207.31 |
| Ecuador | 4975 (4021,5988) | 109.78 (90.42,130.68) | 14843 (11786,18496) | 96.93 (77.68,119.73) | 0.03 (-0.02,0.08) | 198.37 |
| Egypt | 12709 (10081,15899) | 76.43 (61.03,96.35) | 30353 (24425,37293) | 75.86 (62.38,91.21) | -0.01 (-0.16,0.14) | 138.84 |
| El Salvador | 3201 (2604,3900) | 113.81 (92.47,138.42) | 8071 (6523,9719) | 119.8 (96.6,144.65) | -0.38 (-0.44,-0.31) | 152.15 |
| Equatorial Guinea | 137 (97,196) | 95.27 (68.76,137.32) | 407 (299,528) | 111.1 (83.53,142.52) | 0.09 (0.02,0.16) | 196.32 |
| Eritrea | 599 (414,827) | 77.05 (53.62,107.15) | 1612 (1063,2379) | 84.53 (54.92,128.65) | 0.14 (0.08,0.21) | 169.1 |
| Estonia | 1863 (1545,2308) | 94.23 (78.75,115.45) | 3559 (3013,4202) | 114.15 (94.72,136.36) | 0.52 (0.45,0.58) | 91.02 |
| Ethiopia | 10134 (6772,13928) | 69.44 (45.56,97.24) | 25179 (17422,35300) | 70.26 (48.29,98.85) | 0.23 (0.19,0.26) | 148.45 |
| Federated States of Micronesia | 65 (50,82) | 166.28 (124.82,215.25) | 95 (74,118) | 173.09 (134.52,216.49) | 0.55 (0.49,0.62) | 46 |
| Fiji | 344 (282,419) | 124.74 (103.61,151.09) | 843 (664,1017) | 144.64 (115.55,171.88) | 0.47 (0.23,0.71) | 145.27 |
| Finland | 13810 (11123,16890) | 190.5 (153.58,231.88) | 18394 (14929,21967) | 127.35 (103.37,152.38) | -0.03 (-0.11,0.05) | 33.19 |
| France | 113184 (92060,139793) | 128.27 (104.29,157.92) | 182910 (150493,221951) | 107.36 (86.51,132.29) | 0.4 (0.31,0.5) | 61.6 |
| Gabon | 575 (454,736) | 122.35 (97.53,157.84) | 993 (774,1212) | 129.32 (101.95,154.99) | -1.47 (-1.6,-1.33) | 72.54 |
| Georgia | 5209 (3807,6550) | 89.4 (64.79,113.19) | 7760 (6545,9213) | 122.39 (102.9,145.33) | -0.6 (-0.65,-0.55) | 48.98 |
| Germany | 201327 (163031,246392) | 152.56 (123.99,185.96) | 397403 (335970,463533) | 176.87 (148.23,208.48) | 0.07 (-0.02,0.15) | 97.39 |
| Ghana | 3553 (2795,4415) | 84.22 (68.07,101.15) | 9818 (7715,12324) | 84.84 (67.39,103.92) | 0.56 (0.52,0.6) | 176.33 |
| Greece | 16059 (13186,19814) | 109.08 (90.27,133.03) | 30788 (25091,37704) | 105.1 (84.64,130.31) | 1 (0.55,1.45) | 91.71 |
| Greenland | 56 (46,68) | 224.14 (184.04,269.42) | 104 (81,130) | 181.89 (144.48,226.12) | 0.72 (0.56,0.89) | 84.28 |
| Grenada | 98 (82,119) | 121.66 (100.98,147.69) | 135 (113,159) | 141.52 (120.78,164.76) | -0.22 (-0.32,-0.12) | 37 |
| Guam | 74 (61,89) | 141.86 (120.45,168.14) | 195 (152,244) | 90.63 (70.16,113.59) | -0.32 (-0.46,-0.19) | 163.47 |
| Guatemala | 2706 (2179,3315) | 111.72 (93.41,136.28) | 9969 (8055,12254) | 102.34 (83.66,124.24) | -0.47 (-0.56,-0.37) | 268.37 |
| Guinea | 1908 (1367,2508) | 72.06 (51.92,95.94) | 3582 (2749,4497) | 82.81 (63.8,103.74) | 0.4 (0.2,0.61) | 87.71 |
| Guinea-Bissau | 229 (171,306) | 85.55 (65.06,112.72) | 417 (314,533) | 94.52 (72.48,119.86) | -0.95 (-1.22,-0.68) | 81.88 |
| Guyana | 371 (302,451) | 116.75 (96.07,140.9) | 681 (543,834) | 127.56 (103.37,152.5) | -0.28 (-0.37,-0.2) | 83.46 |
| Haiti | 3107 (2285,4348) | 130.3 (96.61,182.94) | 6840 (4956,9180) | 127.46 (93.23,171.69) | 0.46 (0.42,0.5) | 120.18 |
| Honduras | 1806 (1390,2322) | 107.39 (82.98,138.54) | 7556 (5799,9174) | 148.88 (115.58,180.09) | 0.33 (0.31,0.36) | 318.4 |
| Hungary | 15494 (12523,19139) | 109.79 (90.18,134.09) | 19640 (15966,23931) | 92.84 (75.06,114.33) | 0.32 (0.17,0.48) | 26.75 |
| Iceland | 388 (324,467) | 128.16 (106.36,154.7) | 945 (793,1109) | 144.46 (120.93,170.09) | -0.02 (-0.05,0.01) | 143.56 |
| India | 239570 (175322,323221) | 69.36 (51.38,93.59) | 855145 (645560,1094554) | 86.05 (65.71,108.52) | 1.12 (0.96,1.28) | 256.95 |
| Indonesia | 75340 (58204,96155) | 101.59 (77.89,129.42) | 223354 (178284,274232) | 133.13 (106.77,160.61) | -0.44 (-0.5,-0.37) | 196.46 |
| Iraq | 5572 (4348,6978) | 76.45 (59.52,96.57) | 16445 (13020,19938) | 93.63 (74.24,114) | 0.53 (0.43,0.63) | 195.13 |
| Ireland | 5569 (4632,6722) | 138.95 (116.12,166.07) | 9551 (7848,11621) | 113.62 (93.02,138.37) | 0.82 (0.7,0.94) | 71.5 |
| Islamic Republic of Iran | 12116 (9430,15214) | 70.54 (55.4,88.33) | 47525 (37528,58271) | 72.39 (57.36,88.32) | 0.88 (0.82,0.94) | 292.26 |
| Israel | 6749 (5501,8272) | 145.13 (119.45,175.94) | 19691 (15654,24214) | 148.81 (117.35,184.36) | 0.02 (-0.03,0.07) | 191.77 |
| Italy | 106933 (81969,136680) | 120.11 (93.1,152.35) | 206967 (163080,257741) | 116.35 (91.14,146.4) | 0.36 (0.24,0.47) | 93.55 |
| Jamaica | 2149 (1777,2606) | 116.99 (96.82,142.47) | 4111 (3349,4961) | 123.13 (99.37,149.66) | -0.75 (-0.89,-0.6) | 91.3 |
| Japan | 132020 (106096,163379) | 81.38 (66.07,100.34) | 270641 (220302,330702) | 61.78 (50.37,76.42) | 0.36 (0.17,0.55) | 105 |
| Jordan | 658 (526,813) | 68.66 (55.41,84.59) | 3377 (2602,4223) | 63.06 (49.36,78.25) | -0.08 (-0.19,0.03) | 413.02 |
| Kazakhstan | 8359 (6442,11000) | 74.49 (57.9,97.3) | 12990 (10082,16723) | 85.35 (68.14,108.69) | 0.18 (0.07,0.29) | 55.4 |
| Kenya | 4433 (3231,5797) | 67.09 (48.23,86.25) | 13715 (9944,18038) | 80.37 (57.75,106.07) | -1.25 (-1.53,-0.97) | 209.38 |
| Kingdom of Eswatini | 180 (139,236) | 84.13 (64.91,109.9) | 372 (283,474) | 88.58 (67.65,110.66) | -0.38 (-0.54,-0.23) | 106 |
| Kiribati | 27 (22,34) | 93.66 (74.45,116.98) | 56 (44,70) | 102.38 (80.22,126) | 0.22 (0.11,0.33) | 106.79 |
| Kuwait | 272 (217,339) | 62.26 (49.55,76.91) | 1525 (1212,1889) | 66.57 (52.84,82.17) | 0.67 (0.61,0.73) | 460.44 |
| Kyrgyzstan | 1740 (1344,2258) | 64.88 (50.53,83.45) | 2816 (2187,3622) | 69.06 (54.42,88.3) | 0.24 (0.19,0.29) | 61.9 |
| Lao People's Democratic Republic | 1536 (1129,2018) | 102.38 (75.79,136.34) | 3842 (2968,4839) | 111.23 (87.57,138.44) | 0.4 (0.08,0.72) | 150.12 |
| Latvia | 3115 (2573,3792) | 88.84 (73.85,107.54) | 4759 (4088,5510) | 107.11 (90.35,125.03) | 0.22 (0.13,0.31) | 52.76 |
| Lebanon | 1614 (1080,2271) | 92.94 (60.68,132.36) | 4546 (3720,5509) | 68.9 (56.31,84.18) | 0.23 (0.21,0.25) | 181.63 |
| Lesotho | 451 (340,597) | 63.7 (47.34,85.86) | 699 (534,890) | 83.87 (64.5,105.66) | 0.84 (0.72,0.96) | 54.8 |
| Liberia | 690 (498,882) | 80.39 (58.15,101.4) | 1248 (940,1587) | 85.7 (65.43,107.19) | -1.13 (-1.25,-1.02) | 80.89 |
| Libya | 978 (741,1255) | 60.2 (45.09,77.15) | 2950 (2127,3911) | 70.05 (50.35,92.29) | 1.43 (1.19,1.67) | 201.68 |
| Lithuania | 4073 (3334,5057) | 91.65 (75.55,113.69) | 6940 (5789,8373) | 107.61 (88.1,131.18) | 0.22 (0.19,0.26) | 70.36 |
| Luxembourg | 775 (667,905) | 143.61 (124.34,166.52) | 1569 (1347,1837) | 133.18 (114.42,156.51) | 0.8 (0.67,0.92) | 102.46 |
| Madagascar | 4041 (2847,5160) | 101.25 (70.8,129.17) | 8193 (5940,10725) | 104.41 (74.57,139) | 0.55 (0.48,0.62) | 102.74 |
| Malawi | 1878 (1295,2561) | 64.69 (43.61,87.19) | 4339 (3177,5754) | 75.66 (55.07,100.82) | -0.02 (-0.1,0.07) | 131 |
| Malaysia | 7513 (5778,9559) | 92.23 (71.37,117.01) | 26502 (20978,32395) | 112.56 (91.2,135.64) | 0.1 (0.01,0.18) | 252.75 |
| Maldives | 58 (42,77) | 97.45 (71.84,126.11) | 261 (204,337) | 93.33 (73.33,118.42) | 0.45 (0.41,0.5) | 351.77 |
| Mali | 1741 (1280,2332) | 63.27 (46.3,84.06) | 4155 (3145,5466) | 66.21 (50.52,84.94) | 0.75 (0.58,0.92) | 138.68 |
| Malta | 487 (399,589) | 121.54 (100.54,145.77) | 1180 (1005,1388) | 107.08 (90.69,126.34) | -0.31 (-0.39,-0.24) | 142.14 |
| Marshall Islands | 19 (15,23) | 145.78 (115.76,183.43) | 41 (30,53) | 156.76 (122.5,196.88) | 0.22 (0.14,0.29) | 120.35 |
| Mauritania | 682 (546,834) | 89.92 (72.58,109.98) | 1595 (1232,1996) | 95.91 (73.63,118.22) | -0.16 (-0.3,-0.03) | 133.88 |
| Mauritius | 654 (537,792) | 116.21 (97.68,138.52) | 1849 (1526,2231) | 109.11 (90.73,131.02) | 0.18 (0.11,0.24) | 182.87 |
| Mexico | 42540 (34462,52381) | 124.51 (103.13,149.87) | 139470 (110869,170421) | 119.77 (96.17,145.26) | 0.07 (-0.01,0.14) | 227.85 |
| Mongolia | 639 (483,839) | 69.37 (53.04,90.7) | 1288 (954,1703) | 69.41 (52.42,89.83) | -0.37 (-0.49,-0.24) | 101.68 |
| Montenegro | 1184 (1023,1423) | 204.4 (176.69,246.84) | 2331 (1944,2768) | 266.09 (221.18,320.05) | -0.08 (-0.11,-0.04) | 96.95 |
| Morocco | 7964 (5788,10135) | 67.34 (48.44,86.56) | 23355 (18287,29438) | 83.58 (65.59,104.3) | 0.11 (0.09,0.13) | 193.27 |
| Mozambique | 3346 (2241,4552) | 75 (50.57,101.97) | 7582 (5257,10573) | 92.79 (65.44,130.27) | -0.24 (-0.33,-0.15) | 126.59 |
| Myanmar | 17968 (13420,23816) | 103.21 (77.79,135.89) | 43946 (34393,56178) | 111.54 (88.01,140.63) | -0.18 (-0.24,-0.11) | 144.58 |
| Namibia | 362 (278,466) | 75.68 (58.72,97.54) | 897 (677,1147) | 84.98 (63.14,107.72) | 1.04 (0.81,1.28) | 147.8 |
| Nepal | 4603 (3202,6368) | 66.88 (45.87,93.49) | 16079 (11993,21120) | 87.28 (65.75,116.28) | 0.74 (0.67,0.82) | 249.33 |
| Netherlands | 30426 (26007,35157) | 148.71 (126.96,172.08) | 50673 (42989,59870) | 130.44 (110.01,154.87) | 0.92 (0.84,1) | 66.55 |
| New Zealand | 6138 (5194,7226) | 161.54 (137.35,188.98) | 14013 (11814,16401) | 154.18 (130.39,181.27) | 0.12 (0.06,0.17) | 128.28 |
| Nicaragua | 1252 (974,1576) | 95.1 (74.75,119.04) | 4233 (3296,5278) | 97.61 (76.83,120.84) | 0.33 (0.21,0.44) | 238.17 |
| Niger | 1154 (795,1596) | 62.61 (41.76,88.15) | 3721 (2518,5115) | 66.3 (43.73,90.92) | 0.42 (0.37,0.47) | 222.48 |
| Nigeria | 29033 (22706,36923) | 90.67 (71.86,115.03) | 56183 (42281,70658) | 87.08 (65.99,108.4) | 0.94 (0.82,1.07) | 93.51 |
| Northern Mariana Islands | 18 (14,22) | 144.3 (118.96,173.08) | 64 (53,78) | 158.63 (132.79,185.55) | -0.69 (-0.81,-0.58) | 254.17 |
| Norway | 10706 (8932,12818) | 143.49 (119.37,171.91) | 14671 (12145,17639) | 128.56 (105.7,156.78) | 0 (-0.12,0.12) | 37.03 |
| Oman | 413 (310,526) | 80 (60.86,102.78) | 1237 (991,1518) | 90.96 (74.65,109.22) | 0.22 (0.11,0.34) | 199.54 |
| Pakistan | 36868 (26780,49762) | 79.54 (57.9,106.28) | 90387 (69208,116432) | 101.51 (79.65,131.68) | 0.19 (0.16,0.23) | 145.16 |
| Palestine | 583 (451,731) | 87.07 (67.42,108.73) | 1522 (1249,1838) | 89.76 (74.27,107.6) | -0.3 (-0.38,-0.22) | 161.17 |
| Panama | 1430 (1151,1761) | 105.41 (85.8,129.16) | 5027 (3957,6123) | 110.42 (86.72,134.83) | -0.01 (-0.04,0.03) | 251.61 |
| Papua New Guinea | 1362 (950,1811) | 97.03 (69.37,127.75) | 4124 (2976,5545) | 102.24 (75.12,137.62) | 0.72 (0.41,1.03) | 202.73 |
| Paraguay | 2431 (1947,3092) | 120.41 (97.06,152.77) | 7067 (5557,8778) | 130.91 (103.06,161.94) | 0.29 (0.16,0.41) | 190.71 |
| Peru | 11138 (8738,13510) | 103.1 (81.39,125.11) | 32300 (24716,40452) | 97.33 (74.34,122.44) | -0.44 (-0.59,-0.3) | 190 |
| Philippines | 23120 (18497,29118) | 111.79 (90.94,136.5) | 72144 (56995,88259) | 110.96 (89.17,135.18) | 0.73 (0.51,0.96) | 212.04 |
| Plurinational State of Bolivia | 3127 (2335,4175) | 121.39 (91.49,162.71) | 9440 (7098,11874) | 123.22 (93.91,154.08) | 0.7 (0.56,0.84) | 201.89 |
| Poland | 57914 (49713,67500) | 141.14 (122.41,163.83) | 99702 (80721,122377) | 130.17 (104.84,160.59) | 0.04 (0,0.08) | 72.16 |
| Portugal | 15111 (11965,19103) | 116.12 (93.14,144.2) | 28100 (23004,34125) | 96.53 (78.19,118.23) | -0.01 (-0.14,0.11) | 85.96 |
| Principality of Monaco | 93 (73,117) | 117.26 (90.98,147.88) | 125 (97,156) | 109.17 (84.56,138.67) | 0.18 (0.14,0.22) | 34.17 |
| Puerto Rico | 4226 (3445,5170) | 126.54 (104.91,151.88) | 8769 (7003,10860) | 104.23 (82.57,131.44) | 0.16 (0.13,0.18) | 107.49 |
| Qatar | 62 (41,78) | 116.22 (65.7,150.08) | 408 (298,555) | 78.49 (45.53,103.69) | 0.4 (0.32,0.48) | 556.7 |
| Republic of Cabo Verde | 175 (134,220) | 73.39 (56.36,91.97) | 387 (300,476) | 93.89 (72.94,115.1) | -0.29 (-0.44,-0.13) | 121.56 |
| Republic of C么te d'Ivoire | 1990 (1499,2529) | 82.32 (63.19,102.24) | 6579 (5111,8188) | 89.33 (71.39,107.83) | 0.12 (0.06,0.18) | 230.57 |
| Republic of Korea | 22988 (17736,30412) | 93.85 (73.56,124.39) | 89265 (71029,110072) | 96.31 (76.81,117.99) | -0.34 (-0.7,0.02) | 288.31 |
| Republic of Moldova | 3481 (2822,4378) | 94.5 (78.13,116.9) | 6007 (4883,7306) | 99.18 (80.48,120.79) | -0.67 (-0.75,-0.59) | 72.56 |
| Republic of Nauru | 6 (5,8) | 179.67 (145.62,221.17) | 9 (7,11) | 204.45 (146.73,299.14) | -0.73 (-0.79,-0.66) | 40.48 |
| Republic of Niue | 4 (3,4) | 151.06 (125.32,182.25) | 3 (3,4) | 155.44 (124.6,184.55) | -1.66 (-2.1,-1.22) | -12.37 |
| Republic of Palau | 9 (7,11) | 114.57 (91.97,138.85) | 19 (15,25) | 113.24 (92.02,140.01) | 0.39 (0.24,0.53) | 114.42 |
| Republic of San Marino | 50 (40,62) | 133.98 (106.29,165.98) | 84 (64,111) | 92.22 (69.55,122.85) | 0.08 (-0.01,0.16) | 68.04 |
| Republic of the Gambia | 189 (142,244) | 77.86 (58.66,99.21) | 680 (527,843) | 93.4 (73.11,114.03) | -0.79 (-0.95,-0.62) | 259.07 |
| Romania | 22242 (17716,28195) | 90.76 (73.88,112.51) | 31380 (26640,36872) | 77.45 (65.23,91.39) | 0.43 (0.33,0.54) | 41.08 |
| Russian Federation | 156916 (126821,199504) | 97.18 (79.68,121.88) | 271806 (222263,328016) | 111.42 (91.12,134.81) | -0.98 (-1.19,-0.77) | 73.22 |
| Rwanda | 1848 (1199,2515) | 86.99 (54.74,118.73) | 3524 (2244,5177) | 73.71 (45.42,110.15) | -0.12 (-0.24,0) | 90.65 |
| Saint Kitts and Nevis | 55 (46,65) | 158.08 (133.38,183.44) | 77 (63,92) | 145.54 (123.59,168.74) | -1.24 (-1.43,-1.04) | 39.13 |
| Saint Lucia | 125 (106,146) | 175.93 (153.53,200.83) | 316 (255,375) | 137.1 (111.28,162.68) | -0.17 (-0.31,-0.03) | 152.22 |
| Saint Vincent and the Grenadines | 97 (84,115) | 150.93 (131.3,175.74) | 182 (152,215) | 141.74 (120.9,165.99) | 0.03 (-0.02,0.08) | 86.67 |
| Samoa | 99 (76,125) | 143.82 (110.5,180.37) | 180 (143,224) | 146.13 (115.9,180.16) | -0.86 (-1.03,-0.69) | 81.72 |
| Sao Tome and Principe | 38 (30,52) | 71.04 (54.61,98.35) | 74 (59,90) | 91.18 (73.63,109.63) | 0.94 (0.9,0.99) | 91.86 |
| Saudi Arabia | 2831 (2245,3620) | 64.02 (50.22,81.43) | 9049 (7157,11409) | 72.24 (58.16,88.58) | 0.35 (0.29,0.42) | 219.61 |
| Senegal | 1915 (1430,2460) | 78.78 (58.9,101.14) | 5350 (4182,6681) | 92.09 (72.76,112.97) | 0.41 (0.37,0.45) | 179.41 |
| Serbia | 10432 (8568,12759) | 120.07 (99.86,144.1) | 16205 (13504,19228) | 92.99 (77.52,110.36) | -1.12 (-1.35,-0.89) | 55.34 |
| Seychelles | 62 (51,76) | 112.57 (91.59,137.49) | 113 (90,136) | 114.22 (91.68,137.86) | 0.16 (0.05,0.26) | 81.45 |
| Sierra Leone | 1267 (923,1667) | 76.13 (54.99,99.73) | 2275 (1713,2918) | 80.38 (61.47,101.1) | 0.18 (0.16,0.2) | 79.58 |
| Singapore | 1383 (1082,1754) | 69.05 (54.34,86.24) | 4278 (3235,5584) | 50.52 (38.39,65.59) | -1.26 (-1.4,-1.12) | 209.43 |
| Slovakia | 8402 (6989,10006) | 144.92 (121.43,171.5) | 14612 (11797,17705) | 150.56 (121.36,182.31) | 0.18 (0.08,0.28) | 73.91 |
| Slovenia | 2338 (1905,2882) | 95.87 (78.17,117.88) | 4654 (3899,5468) | 92.57 (76.97,109.58) | 0.02 (-0.2,0.25) | 99.08 |
| Socialist Republic of Viet Nam | 33051 (25239,41932) | 95.92 (73.96,120.29) | 97149 (74777,122074) | 118.87 (91.48,147.64) | 0.34 (0.31,0.36) | 193.94 |
| Solomon Islands | 102 (69,139) | 103.32 (67.79,140.33) | 306 (217,408) | 115.15 (78.89,160.17) | -0.27 (-0.32,-0.22) | 198.96 |
| Somalia | 1182 (742,1682) | 70.06 (43.56,99.59) | 2767 (1735,4169) | 63.59 (39.51,97.45) | 0.58 (0.39,0.77) | 134.12 |
| South Africa | 13583 (10577,17397) | 76.09 (59.69,97.65) | 34786 (28411,42506) | 91.41 (75.11,110.29) | -0.21 (-0.31,-0.12) | 156.1 |
| South Sudan | 1617 (1007,2226) | 77.27 (48.54,105.52) | 2148 (1433,2959) | 73.44 (48.25,101.88) | -0.26 (-0.35,-0.18) | 32.82 |
| Spain | 71587 (57640,88764) | 132 (107.21,161.79) | 140604 (117419,165622) | 119.53 (98.17,143.01) | 0.48 (0.35,0.61) | 96.41 |
| Sri Lanka | 7446 (5696,9311) | 90.36 (70.8,111.36) | 22556 (16762,28901) | 95.58 (71.97,120.57) | 0.34 (0.28,0.4) | 202.94 |
| Sudan | 4646 (3340,6250) | 64.76 (46.08,88.58) | 10967 (8324,13689) | 72.54 (56.08,90.42) | -0.06 (-0.15,0.03) | 136.07 |
| Suriname | 291 (241,350) | 127.17 (105.45,152.1) | 706 (544,876) | 119.94 (93.43,148.75) | 1.61 (1.46,1.77) | 142.43 |
| Sweden | 22758 (17956,28457) | 137.78 (108.56,173.28) | 57180 (45730,70747) | 222 (175.69,277.17) | 0.37 (0.17,0.57) | 151.26 |
| Switzerland | 7852 (6377,9611) | 70.15 (56.77,85.88) | 14918 (12437,17545) | 69.34 (57.59,82.94) | -0.02 (-0.14,0.09) | 89.99 |
| Syrian Arab Republic | 3247 (2558,4087) | 79.86 (62.62,101.42) | 8108 (6087,10069) | 85.65 (63.76,105.23) | -0.83 (-1.02,-0.63) | 149.75 |
| Taiwan (Province of China) | 13126 (10402,16164) | 112.17 (92.95,134.45) | 41012 (33444,50586) | 92.14 (74.48,113.99) | -0.11 (-0.22,0) | 212.45 |
| Tajikistan | 1358 (1009,1881) | 55.03 (41.21,75.82) | 2547 (1826,3444) | 54.26 (39.71,72.32) | -0.68 (-0.8,-0.56) | 87.59 |
| Thailand | 30152 (23731,38013) | 112.77 (89.31,141.37) | 110567 (87502,136071) | 101.05 (80.12,124.39) | 0.46 (0.39,0.53) | 266.7 |
| The former Yugoslav Republic of Macedonia | 1732 (1418,2143) | 106.33 (88.49,129.52) | 3664 (2801,4933) | 136.26 (107.56,174.16) | 0.38 (0.35,0.4) | 111.54 |
| Timor-Leste | 179 (131,233) | 90.69 (66.63,119.68) | 692 (531,906) | 102.36 (80.67,131.25) | -0.06 (-0.09,-0.03) | 287.4 |
| Togo | 674 (522,859) | 78.55 (61.25,99.57) | 2198 (1650,2761) | 89.23 (69.02,110.26) | 0.3 (0.23,0.37) | 226.04 |
| Tokelau | 2 (1,2) | 152.92 (124.85,190.22) | 2 (2,3) | 149.97 (112.24,200.51) | -0.43 (-0.49,-0.37) | 19.73 |
| Tonga | 51 (39,67) | 109.91 (83.21,144.28) | 91 (71,117) | 119.97 (93.23,153.34) | 0.36 (0.32,0.41) | 78.83 |
| Trinidad and Tobago | 1001 (827,1210) | 147.3 (125.02,173.65) | 2386 (1898,2915) | 128.6 (102.59,156.53) | -0.11 (-0.46,0.25) | 138.28 |
| Tunisia | 2561 (1967,3177) | 70.85 (55.59,87.76) | 9283 (6895,12247) | 81.82 (60.46,107.55) | 0.53 (0.44,0.61) | 262.49 |
| Turkey | 19964 (15940,24779) | 72.06 (57.9,89.75) | 55942 (46418,65880) | 67.92 (56.42,80.43) | 0.01 (-0.02,0.03) | 180.22 |
| Turkmenistan | 1138 (887,1460) | 70.46 (55.65,90.88) | 2975 (2299,3738) | 86.5 (67.67,106.69) | -0.01 (-0.07,0.04) | 161.51 |
| Tuvalu | 8 (6,10) | 149.09 (117.45,187.98) | 13 (10,16) | 150.94 (121.58,183.48) | 0.04 (-0.07,0.14) | 71.35 |
| Uganda | 3639 (2371,5067) | 70.74 (45.45,99.84) | 8476 (5794,12027) | 73.52 (49.36,105.55) | 1.96 (1.34,2.58) | 132.91 |
| Ukraine | 61515 (49362,75818) | 92.65 (75.62,113.39) | 78770 (61400,97874) | 98.56 (76.69,121.96) | 0.08 (-0.03,0.19) | 28.05 |
| United Arab Emirates | 232 (171,301) | 79.54 (60.3,104.87) | 1763 (1273,2342) | 85.82 (66.1,105.16) | -0.06 (-0.23,0.12) | 660.31 |
| United Kingdom of Great Britain and Northern Ireland | 110739 (93505,131324) | 117 (99.11,138.75) | 176779 (150586,207501) | 119.73 (101.41,141.22) | 0.68 (0.64,0.72) | 59.64 |
| United Republic of Tanzania | 6566 (4530,8842) | 79.46 (53.67,107.3) | 16779 (11752,23254) | 82.33 (57.78,113.53) | -0.79 (-0.92,-0.67) | 155.54 |
| United States of America | 393871 (306983,495874) | 118.44 (92.41,149.14) | 916645 (768611,1080294) | 146.72 (122.77,172.78) | 0.24 (0.18,0.3) | 132.73 |
| United States Virgin Islands | 96 (77,118) | 149.87 (122.66,179.17) | 201 (151,252) | 113.8 (87.47,142) | 0.59 (0.52,0.65) | 109.06 |
| Uruguay | 2984 (2412,3683) | 77.36 (62.87,95.1) | 5256 (4397,6336) | 82.04 (68.63,99.71) | 0.14 (0.11,0.18) | 76.16 |
| Uzbekistan | 5343 (3749,7549) | 50.19 (35.2,71.03) | 12966 (9657,17427) | 58.96 (44.73,77.9) | -0.16 (-0.23,-0.08) | 142.7 |
| Vanuatu | 58 (41,76) | 120.37 (87.79,158.55) | 175 (135,222) | 127.94 (98.97,160.53) | 0.74 (0.69,0.78) | 204.34 |
| Yemen | 2246 (1544,3172) | 65.51 (44.69,94.69) | 7685 (5625,9928) | 75.17 (55.62,97.35) | 0.41 (0.38,0.44) | 242.14 |
| Zambia | 1828 (1391,2272) | 86.65 (65.76,107.38) | 5743 (3778,9002) | 112.52 (76.94,168.22) | 0.93 (0.72,1.14) | 214.11 |
| Zimbabwe | 2159 (1653,2691) | 72.21 (55.8,88.6) | 4171 (3086,5215) | 82.48 (61.95,102) | 0.67 (0.46,0.87) | 93.21 |

AF/AFL, Atrial fibrillation (AF)/atrial flutter (AFL); CI, confidence interval; EAPC, estimated annual percentage change; UI, uncertainty interval; DALYs, disability-adjusted life years.

Supplementary Table 8 The APC and AAPC in case number of AF/AFL incidence, prevalence ,death and DALYs for both sexes from 1991 to 2021.

| **Number** | | | | | | | | | | | |
| --- | --- | --- | --- | --- | --- | --- | --- | --- | --- | --- | --- |
| **incidence** | | | **prevalence** | | | **death** | | | **DALYs** | | |
| **year** | **APC** | **P** | **year** | **APC** | **P** | **year** | **APC** | **P** | **year** | **APC** | **P** |
| 1990-1996 | 2.049(2.013,2.086) | < 0.001 | 1990-1996 | 2.253(2.222,2.283) | < 0.001 | 1990-1995 | 3.941(3.746,4.136) | < 0.001 | 1990-1994 | 2.866(2.738,2.9994) | < 0.001 |
| 1996-2021 | 2.305(2.24,2.371) | < 0.001 | 1996-2021 | 2.649(2.596,2.703) | < 0.001 | 1995-2007 | 3.279(3.227,3.332) | < 0.001 | 1994-2000 | 2.689(2.602,2.775) | < 0.001 |
| 2001-2004 | 2.958(2.766,3.15) | < 0.001 | 2001-2004 | 3.319(3.164,3.474) | < 0.001 | 2007-2018 | 4.070(4.006,4.133) | < 0.001 | 2000-2004 | 3.161(2.973,3.349) | < 0.001 |
| 2004-2010 | 2.452(2.412,2.493) | < 0.001 | 2004-2010 | 2.667(2.634,2.7) | < 0.001 | 2018-2021 | 2.384(1.925,2.846) | < 0.001 | 2004-2007 | 2.774(2.409,3.141) | < 0.001 |
| 2010-2015 | 2.802(2.744,2.86) | < 0.001 | 2010-2015 | 2.892(2.845,2.94) | < 0.001 |  |  |  | 2007-2013 | 3.131(3.05,3.213) | < 0.001 |
| 2015-2021 | 3.324(3.292,3.356) | < 0.001 | 2015-2021 | 3.363(3.337,3.89) | < 0.001 |  |  |  | 2013-2019 | 3.381(3.3,3.462) | < 0.001 |
|  |  |  |  |  |  |  |  |  | 2019-2021 | 2.485(2.121,2.85) | < 0.001 |
| 1990-2021 | 2.624(2.599,2.648) | < 0.001 | 1990-2021 | 2.817(2.798,2.837) | < 0.001 | 1990-2021 | 3.578(3.519,3.638) | < 0.001 | 1990-2021 | 2.987(2.934,3.04) | < 0.001 |
| 2010-2021 | 3.086(3.057,3.116) | < 0.001 | 2010-2021 | 3.149(3.125,3.173) | < 0.001 | 2010-2021 | 3.607(3.48,3.735) | < 0.001 | 2010-2021 | 3.150(3.075,3.224) | < 0.001 |
| 1990-1999 | 2.135(2.104,2.165) | < 0.001 | 1990-1999 | 2.385(2.36,2.409) | < 0.001 | 1990-1999 | 3.646(3.542,3.75) | < 0.001 | 1990-1999 | 2.767(2.7,2.834) | < 0.001 |
| 2000-2009 | 2.604(2.542,2.667) | < 0.001 | 2000-2009 | 2.882(2.831,2.932) | < 0.001 | 2000-2009 | 3.454(3.414,3.495) | < 0.001 | 2000-2009 | 3.025(2.891,3.16) | < 0.001 |

APC, annual percentage change; AAPC, average annual percentage change; DALYs, disability-adjusted life years.

Supplementary Table 9 The APC and AAPC in ASR of AF/AFL incidence, prevalence, death and DALYs for both sexes from 1991 to 2021

| **ASR** | | | | | | | | | | | |
| --- | --- | --- | --- | --- | --- | --- | --- | --- | --- | --- | --- |
| **incidence** | | | **prevalence** | | | **death** | | | **DALYs** | | |
| **year** | **APC** | **P** | **year** | **APC** | **P** | **year** | **APC** | **P** | **year** | **APC** | **P** |
| 1990-1995 | -0.331(-0.344,-0.318) | < 0.001 | 1990-1995 | -0.229(-0.256,-0.201) | < 0.001 | 1990-1995 | 0.665(0.517,0.813) | < 0.001 | 1990-1995 | 0.219(0.13,0.307) | < 0.001 |
| 1995-2001 | -0.192(-0.205,-0.18) | < 0.001 | 1995-2001 | -0.005(-0.03,0.021) | 0.699 | 1995-2003 | 0.162(0.083,0.242) | 0.001 | 1995-2001 | 0.02(-0.065,0.104) | 0.62 |
| 2001-2004 | 0.348(0.297,0.4) | < 0.001 | 2001-2004 | 0.564(0.46,0.668) | < 0.001 | 2003-2006 | -0.404(-0.983,0.177) | 0.159 | 2001-2004 | 0.292(-0.069,0.655) | 0.103 |
| 2004-2010 | -0.307(-0.318,-0.296) | < 0.001 | 2004-2011 | -0.285(-0.301,-0.268) | < 0.001 | 2006-2012 | 0.059(-0.075,0.192) | 0.362 | 2004-2007 | -0.333(-0.694,0.029) | 0.068 |
| 2010-2015 | -0.065(-0.081,0.05) | < 0.001 | 2011-2015 | -0.113(-0.163,-0.064) | < 0.001 | 2012-2018 | 0.365(0.224,0.506) | < 0.001 | 2007-2013 | -0.135(-0.216,-0.054) | 0.003 |
| 2015-2019 | 0.384(0.359-0.409) | < 0.001 | 2015-2019 | 0.291(0.241,0.342) | < 0.001 | 2018-2018 | -0.878(-1.218,-0.536) | < 0.001 | 2013-2018 | 0.189(0.074,0.304) | 0.004 |
| 2019-2021 | 0.787(0.735,0.838) | < 0.001 | 2019-2021 | 0.682(0.578,0.787) | < 0.001 |  |  |  | 2018-2021 | -0.179(-0.362,0.005) | 0.056 |
| 1990-2021 | -0.027(-0.034,-0.02) | < 0.001 | 1990-2021 | 0.019(0.004,0.034) | 0.014 | 1990-2021 | 0.106(0.031,0.181) | 0.006 | 1990-2021 | 0.022(-0.034,0.078) | 0.438 |
| 2010-2021 | 0.253(0.239,0.266) | < 0.001 | 2010-2021 | 0.162(0.134,0.191) | < 0.001 | 2010-2021 | -0.031(-0.145,0.082) | 0.591 | 2010-2021 | 0(-0.068,0.068) | 0.995 |
| 1990-1999 | -0.269(-0.278,-0.261) | < 0.001 | 1990-1999 | -0.129(-0.146,-0.112) | < 0.001 | 1990-1999 | 0.441(0.359,0.523) | < 0.001 | 1990-1999 | 0.130(0.075,0.186) | < 0.001 |
| 2000-2009 | -0.076(-0.093,-0.06) | < 0.001 | 2000-2009 | 0.029(-0.004,0.061) | 0.081 | 2000-2009 | -0.061(-0.246,0.123) | 0.515 | 2000-2009 | -0.042(-0.196,0.113) | 0.596 |

APC, annual percentage change; AAPC, average annual percentage change; ASR, age-standardized rate; DALYs, disability-adjusted life years.

Supplementary Table 10 The effects of age, period, and birth cohort on the relative risk of AF/AFL incidence, prevalence, and death

| **Factor** | **Incidence** | | **Prevalence** | | **Death** | |
| --- | --- | --- | --- | --- | --- | --- |
|  | **RR(95%CI)** | **P** | **RR(95%CI)** | **P** | **RR(95%CI)** | **P** |
| Age(years) |  |  |  |  |  |  |
| 30-34 | 0.066 (0.065,0.066) | <0.001 | 0.015 (0.015,0.016) | <0.001 | 0.022 (0.02,0.024) | <0.001 |
| 35-39 | 0.181 (0.18,0.182) | <0.001 | 0.093 (0.093,0.094) | <0.001 | 0.029 (0.027,0.031) | <0.001 |
| 40-44 | 0.311 (0.311,0.312) | <0.001 | 0.23 (0.23,0.23) | <0.001 | 0.066 (0.063,0.069) | <0.001 |
| 45-49 | 0.446 (0.445,0.447) | <0.001 | 0.422 (0.422,0.422) | <0.001 | 0.142 (0.138,0.147) | <0.001 |
| 50-54 | 0.671 (0.669,0.672) | <0.001 | 0.668 (0.667,0.668) | <0.001 | 0.271 (0.264,0.278) | <0.001 |
| 55-59 | 0.959 (0.957,0.961) | <0.001 | 1.01 (1.009,1.011) | <0.001 | 0.468 (0.458,0.478) | <0.001 |
| 60-64 | 1.444 (1.442,1.447) | <0.001 | 1.457 (1.456,1.458) | <0.001 | 0.735 (0.723,0.748) | <0.001 |
| 65-69 | 2.061 (2.057,2.064) | <0.001 | 2.102 (2.1,2.103) | <0.001 | 1.118 (1.102,1.134) | <0.001 |
| 70-74 | 2.561 (2.557,2.566) | <0.001 | 2.868 (2.867,2.87) | <0.001 | 2.207 (2.183,2.232) | <0.001 |
| 75-79 | 2.921 (2.915,2.926) | <0.001 | 3.651 (3.649,3.652) | <0.001 | 4.065 (4.029,4.102) | <0.001 |
| 80-84 | 2.97 (2.964,2.977) | <0.001 | 4.327 (4.325,4.33) | <0.001 | 9.265 (9.19,9.341) | <0.001 |
| 85-89 | 2.691 (2.683,2.698) | <0.001 | 4.652 (4.649,4.655) | <0.001 | 16.826 (16.674,16.98) | <0.001 |
| 90-94 | 2.41 (2.401,2.418) | <0.001 | 4.465 (4.461,4.468) | <0.001 | 31.653 (31.299,32.01) | <0.001 |
| 95+ | 2.204 (2.191,2.218) | <0.001 | 3.677 (3.672,3.682) | <0.001 | 36.381 (35.856,36.914) | <0.001 |
| Period |  |  |  |  |  |  |
| 1992 | 0.772 (0.77,0.773) | <0.001 | 0.681 (0.681,0.682) | <0.001 | 0.576 (0.57,0.582) | <0.001 |
| 1997 | 0.854 (0.853,0.855) | <0.001 | 0.796 (0.796,0.797) | <0.001 | 0.721 (0.716,0.726) | <0.001 |
| 2002 | 0.957 (0.955,0.958) | <0.001 | 0.941 (0.94,0.941) | <0.001 | 0.895 (0.891,0.899) | <0.001 |
| 2007 | 1.052 (1.05,1.053) | <0.001 | 1.083 (1.082,1.083) | <0.001 | 1.108 (1.103,1.113) | <0.001 |
| 2012 | 1.158 (1.157,1.16) | <0.001 | 1.244 (1.243,1.244) | <0.001 | 1.39 (1.381,1.399) | <0.001 |
| 2017 | 1.302 (1.3,1.304) | <0.001 | 1.455 (1.454,1.456) | <0.001 | 1.745 (1.73,1.761) | <0.001 |
| Birth cohort |  |  |  |  |  |  |
| 1897-1901 | 2.741 (2.685,2.798) | <0.001 | 4.242 (4.223,4.261) | <0.001 | 7.366 (7.162,7.576) | <0.001 |
| 1902-1906 | 2.439 (2.416,2.463) | <0.001 | 3.483 (3.476,3.49) | <0.001 | 5.823 (5.712,5.937) | <0.001 |
| 1907-1911 | 2.155 (2.142,2.168) | <0.001 | 2.921 (2.917,2.925) | <0.001 | 4.702 (4.63,4.776) | <0.001 |
| 1912-1916 | 1.907 (1.899,1.916) | <0.001 | 2.472 (2.469,2.474) | <0.001 | 3.856 (3.806,3.907) | <0.001 |
| 1917-1921 | 1.705 (1.698,1.711) | <0.001 | 2.079 (2.077,2.081) | <0.001 | 3.094 (3.058,3.13) | <0.001 |
| 1922-1926 | 1.506 (1.501,1.512) | <0.001 | 1.785 (1.784,1.787) | <0.001 | 2.529 (2.501,2.557) | <0.001 |
| 1927-1931 | 1.335 (1.331,1.339) | <0.001 | 1.52 (1.519,1.522) | <0.001 | 2.033 (2.009,2.057) | <0.001 |
| 1932-1936 | 1.189 (1.186,1.192) | <0.001 | 1.296 (1.294,1.297) | <0.001 | 1.617 (1.595,1.639) | <0.001 |
| 1937-1941 | 1.069 (1.066,1.071) | <0.001 | 1.123 (1.122,1.124) | <0.001 | 1.278 (1.257,1.298) | <0.001 |
| 1942-1946 | 0.972 (0.97,0.975) | <0.001 | 0.975 (0.975,0.976) | <0.001 | 1.017 (0.998,1.037) | <0.001 |
| 1947-1951 | 0.881 (0.879,0.883) | <0.001 | 0.848 (0.847,0.849) | <0.001 | 0.799 (0.782,0.817) | <0.001 |
| 1952-1956 | 0.796 (0.794,0.798) | <0.001 | 0.737 (0.736,0.737) | <0.001 | 0.637 (0.62,0.653) | <0.001 |
| 1957-1961 | 0.731 (0.73,0.733) | <0.001 | 0.644 (0.643,0.645) | <0.001 | 0.508 (0.493,0.523) | <0.001 |
| 1962-1966 | 0.664 (0.662,0.666) | <0.001 | 0.558 (0.558,0.559) | <0.001 | 0.403 (0.389,0.416) | <0.001 |
| 1967-1971 | 0.599 (0.597,0.601) | <0.001 | 0.481 (0.48,0.481) | <0.001 | 0.312 (0.3,0.325) | <0.001 |
| 1972-1976 | 0.538 (0.536,0.54) | <0.001 | 0.409 (0.409,0.41) | <0.001 | 0.251 (0.239,0.264) | <0.001 |
| 1977-1981 | 0.478 (0.476,0.48) | <0.001 | 0.347 (0.346,0.348) | <0.001 | 0.211 (0.196,0.227) | <0.001 |
| 1982-1986 | 0.427 (0.424,0.429) | <0.001 | 0.297 (0.296,0.298) | <0.001 | 0.169 (0.152,0.189) | <0.001 |
| 1987-1991 | 0.384 (0.379,0.389) | <0.001 | 0.257 (0.254,0.259) | <0.001 | 0.132 (0.109,0.16) | <0.001 |

AF/AFL, Atrial fibrillation (AF)/atrial flutter (AFL); RR, Relative Risk; CI, confidence interval.

Supplementary Table 11 The effects of age, period, and birth cohort on the relative risk of AF/AFL incidence ,prevalence and death by sexs

| **Factor** | **Incidence** | | | | **Prevalence** | | | | **Death** | | | |
| --- | --- | --- | --- | --- | --- | --- | --- | --- | --- | --- | --- | --- |
|  | **Male** | | **Female** | | **Male** | | **Female** | | **Male** | | **Female** | |
|  | **RR(95%CI)** | **P** | **RR(95%CI)** | **P** | **RR(95%CI)** | **P** | **RR(95%CI)** | **P** | **RR(95%CI)** | **P** | **RR(95%CI)** | **P** |
| Age(years) |  |  |  |  |  |  |  |  |  |  |  |  |
| 30-34 | 0.07 (0.07,0.071) | <0.001 | 0.059 (0.058,0.06) | <0.001 | 0.016 (0.016,0.016) | <0.001 | 0.014 (0.014,0.014) | <0.001 | 0.023 (0.021,0.026) | <0.001 | 0.021 (0.018,0.024) | <0.001 |
| 35-39 | 0.196 (0.195,0.197) | <0.001 | 0.161 (0.16,0.162) | <0.001 | 0.097 (0.097,0.098) | <0.001 | 0.086 (0.086,0.086) | <0.001 | 0.03 (0.028,0.033) | <0.001 | 0.027 (0.025,0.03) | <0.001 |
| 40-44 | 0.334 (0.333,0.336) | <0.001 | 0.281 (0.28,0.283) | <0.001 | 0.239 (0.239,0.239) | <0.001 | 0.211 (0.211,0.212) | <0.001 | 0.07 (0.066,0.074) | <0.001 | 0.062 (0.058,0.066) | <0.001 |
| 45-49 | 0.476 (0.475,0.478) | <0.001 | 0.407 (0.405,0.408) | <0.001 | 0.436 (0.435,0.436) | <0.001 | 0.392 (0.391,0.393) | <0.001 | 0.151 (0.145,0.157) | <0.001 | 0.131 (0.125,0.138) | <0.001 |
| 50-54 | 0.71 (0.708,0.712) | <0.001 | 0.626 (0.624,0.628) | <0.001 | 0.686 (0.685,0.687) | <0.001 | 0.627 (0.626,0.628) | <0.001 | 0.291 (0.282,0.301) | <0.001 | 0.247 (0.237,0.257) | <0.001 |
| 55-59 | 1.01 (1.008,1.013) | <0.001 | 0.909 (0.906,0.912) | <0.001 | 1.029 (1.028,1.03) | <0.001 | 0.966 (0.965,0.967) | <0.001 | 0.488 (0.475,0.502) | <0.001 | 0.446 (0.432,0.461) | <0.001 |
| 60-64 | 1.476 (1.472,1.48) | <0.001 | 1.438 (1.434,1.441) | <0.001 | 1.466 (1.465,1.468) | <0.001 | 1.428 (1.426,1.429) | <0.001 | 0.754 (0.737,0.771) | <0.001 | 0.721 (0.702,0.74) | <0.001 |
| 65-69 | 2.052 (2.047,2.057) | <0.001 | 2.125 (2.12,2.13) | <0.001 | 2.07 (2.069,2.072) | <0.001 | 2.135 (2.133,2.137) | <0.001 | 1.118 (1.097,1.14) | <0.001 | 1.13 (1.106,1.155) | <0.001 |
| 70-74 | 2.5 (2.493,2.506) | <0.001 | 2.708 (2.701,2.714) | <0.001 | 2.775 (2.773,2.777) | <0.001 | 3 (2.998,3.003) | <0.001 | 2.133 (2.102,2.166) | <0.001 | 2.317 (2.279,2.356) | <0.001 |
| 75-79 | 2.804 (2.796,2.813) | <0.001 | 3.139 (3.132,3.147) | <0.001 | 3.487 (3.484,3.489) | <0.001 | 3.902 (3.899,3.905) | <0.001 | 3.835 (3.788,3.883) | <0.001 | 4.357 (4.3,4.416) | <0.001 |
| 80-84 | 2.777 (2.767,2.788) | <0.001 | 3.256 (3.247,3.265) | <0.001 | 4.115 (4.112,4.119) | <0.001 | 4.687 (4.683,4.691) | <0.001 | 8.68 (8.582,8.779) | <0.001 | 9.971 (9.851,10.093) | <0.001 |
| 85-89 | 2.425 (2.414,2.437) | <0.001 | 2.999 (2.99,3.009) | <0.001 | 4.445 (4.441,4.45) | <0.001 | 5.071 (5.067,5.076) | <0.001 | 15.68 (15.483,15.88) | <0.001 | 18.129 (17.881,18.38) | <0.001 |
| 90-94 | 2.147 (2.133,2.162) | <0.001 | 2.677 (2.665,2.688) | <0.001 | 4.336 (4.33,4.342) | <0.001 | 4.864 (4.859,4.869) | <0.001 | 29.872 (29.412,30.339) | <0.001 | 33.819 (33.245,34.404) | <0.001 |
| 95+ | 2.069 (2.045,2.093) | <0.001 | 2.383 (2.365,2.4) | <0.001 | 3.729 (3.721,3.738) | <0.001 | 3.939 (3.932,3.946) | <0.001 | 34.555 (33.849,35.277) | <0.001 | 38.735 (37.895,39.594) | <0.001 |
| Period |  |  |  |  |  |  |  |  |  |  |  |  |
| 1992 | 0.777 (0.775,0.778) | <0.001 | 0.763 (0.762,0.765) | <0.001 | 0.681 (0.68,0.681) | <0.001 | 0.674 (0.674,0.675) | <0.001 | 0.574 (0.566,0.582) | <0.001 | 0.577 (0.568,0.586) | <0.001 |
| 1997 | 0.86 (0.858,0.862) | <0.001 | 0.845 (0.844,0.847) | <0.001 | 0.795 (0.795,0.796) | <0.001 | 0.792 (0.791,0.792) | <0.001 | 0.72 (0.713,0.727) | <0.001 | 0.722 (0.715,0.729) | <0.001 |
| 2002 | 0.963 (0.962,0.965) | <0.001 | 0.948 (0.946,0.949) | <0.001 | 0.943 (0.942,0.943) | <0.001 | 0.936 (0.936,0.937) | <0.001 | 0.891 (0.885,0.898) | <0.001 | 0.897 (0.892,0.902) | <0.001 |
| 2007 | 1.058 (1.056,1.059) | <0.001 | 1.046 (1.044,1.047) | <0.001 | 1.089 (1.088,1.089) | <0.001 | 1.078 (1.078,1.079) | <0.001 | 1.112 (1.104,1.119) | <0.001 | 1.105 (1.099,1.111) | <0.001 |
| 2012 | 1.153 (1.151,1.155) | <0.001 | 1.168 (1.166,1.17) | <0.001 | 1.245 (1.244,1.246) | <0.001 | 1.251 (1.251,1.252) | <0.001 | 1.4 (1.388,1.412) | <0.001 | 1.384 (1.371,1.397) | <0.001 |
| 2017 | 1.275 (1.272,1.278) | <0.001 | 1.339 (1.336,1.341) | <0.001 | 1.446 (1.445,1.446) | <0.001 | 1.483 (1.482,1.484) | <0.001 | 1.745 (1.724,1.766) | <0.001 | 1.75 (1.726,1.775) | <0.001 |
| Birth cohort |  |  |  |  |  |  |  |  |  |  |  |  |
| 1897-1901 | 2.618 (2.515,2.726) | <0.001 | 2.929 (2.859,3) | <0.001 | 3.927 (3.895,3.96) | <0.001 | 4.638 (4.614,4.663) | <0.001 | 7.291 (6.955,7.643) | <0.001 | 7.576 (7.29,7.872) | <0.001 |
| 1902-1906 | 2.351 (2.309,2.393) | <0.001 | 2.585 (2.556,2.614) | <0.001 | 3.317 (3.305,3.33) | <0.001 | 3.738 (3.728,3.748) | <0.001 | 5.593 (5.433,5.758) | <0.001 | 6.055 (5.886,6.228) | <0.001 |
| 1907-1911 | 2.054 (2.032,2.077) | <0.001 | 2.283 (2.267,2.3) | <0.001 | 2.841 (2.835,2.848) | <0.001 | 3.092 (3.087,3.098) | <0.001 | 4.506 (4.406,4.608) | <0.001 | 4.894 (4.782,5.008) | <0.001 |
| 1912-1916 | 1.813 (1.798,1.829) | <0.001 | 2.019 (2.008,2.03) | <0.001 | 2.445 (2.44,2.449) | <0.001 | 2.582 (2.578,2.586) | <0.001 | 3.706 (3.637,3.775) | <0.001 | 4.01 (3.933,4.089) | <0.001 |
| 1917-1921 | 1.632 (1.621,1.644) | <0.001 | 1.794 (1.786,1.803) | <0.001 | 2.074 (2.07,2.077) | <0.001 | 2.146 (2.143,2.149) | <0.001 | 2.996 (2.947,3.046) | <0.001 | 3.208 (3.153,3.264) | <0.001 |
| 1922-1926 | 1.461 (1.452,1.47) | <0.001 | 1.568 (1.562,1.575) | <0.001 | 1.798 (1.795,1.8) | <0.001 | 1.819 (1.816,1.821) | <0.001 | 2.456 (2.418,2.494) | <0.001 | 2.618 (2.575,2.662) | <0.001 |
| 1927-1931 | 1.303 (1.296,1.311) | <0.001 | 1.376 (1.371,1.381) | <0.001 | 1.533 (1.531,1.535) | <0.001 | 1.53 (1.528,1.532) | <0.001 | 1.982 (1.95,2.014) | <0.001 | 2.098 (2.06,2.136) | <0.001 |
| 1932-1936 | 1.167 (1.161,1.173) | <0.001 | 1.215 (1.211,1.219) | <0.001 | 1.308 (1.307,1.31) | <0.001 | 1.289 (1.287,1.29) | <0.001 | 1.594 (1.565,1.623) | <0.001 | 1.655 (1.62,1.69) | <0.001 |
| 1937-1941 | 1.059 (1.055,1.064) | <0.001 | 1.078 (1.074,1.082) | <0.001 | 1.141 (1.139,1.142) | <0.001 | 1.103 (1.102,1.104) | <0.001 | 1.269 (1.242,1.296) | <0.001 | 1.297 (1.265,1.329) | <0.001 |
| 1942-1946 | 0.974 (0.971,0.978) | <0.001 | 0.966 (0.963,0.969) | <0.001 | 0.995 (0.994,0.996) | <0.001 | 0.946 (0.944,0.947) | <0.001 | 1.017 (0.992,1.043) | 0.189 | 1.023 (0.993,1.053) | 0.139 |
| 1947-1951 | 0.894 (0.891,0.897) | <0.001 | 0.861 (0.858,0.864) | <0.001 | 0.869 (0.868,0.87) | <0.001 | 0.813 (0.811,0.814) | <0.001 | 0.809 (0.786,0.833) | <0.001 | 0.791 (0.764,0.819) | <0.001 |
| 1952-1956 | 0.815 (0.812,0.818) | <0.001 | 0.769 (0.767,0.772) | <0.001 | 0.755 (0.754,0.756) | <0.001 | 0.705 (0.704,0.706) | <0.001 | 0.647 (0.625,0.669) | <0.001 | 0.627 (0.602,0.653) | <0.001 |
| 1957-1961 | 0.755 (0.753,0.758) | <0.001 | 0.698 (0.696,0.701) | <0.001 | 0.66 (0.659,0.661) | <0.001 | 0.615 (0.614,0.616) | <0.001 | 0.519 (0.499,0.539) | <0.001 | 0.496 (0.474,0.52) | <0.001 |
| 1962-1966 | 0.691 (0.689,0.693) | <0.001 | 0.629 (0.627,0.632) | <0.001 | 0.57 (0.569,0.571) | <0.001 | 0.536 (0.535,0.537) | <0.001 | 0.41 (0.392,0.428) | <0.001 | 0.396 (0.375,0.417) | <0.001 |
| 1967-1971 | 0.621 (0.619,0.624) | <0.001 | 0.572 (0.569,0.574) | <0.001 | 0.485 (0.484,0.486) | <0.001 | 0.47 (0.469,0.471) | <0.001 | 0.318 (0.302,0.335) | <0.001 | 0.306 (0.287,0.325) | <0.001 |
| 1972-1976 | 0.556 (0.554,0.559) | <0.001 | 0.517 (0.514,0.52) | <0.001 | 0.41 (0.409,0.411) | <0.001 | 0.403 (0.402,0.405) | <0.001 | 0.255 (0.239,0.273) | <0.001 | 0.247 (0.228,0.268) | <0.001 |
| 1977-1981 | 0.496 (0.493,0.499) | <0.001 | 0.458 (0.455,0.461) | <0.001 | 0.348 (0.347,0.349) | <0.001 | 0.341 (0.34,0.342) | <0.001 | 0.219 (0.199,0.24) | <0.001 | 0.203 (0.182,0.227) | <0.001 |
| 1982-1986 | 0.446 (0.442,0.449) | <0.001 | 0.406 (0.402,0.41) | <0.001 | 0.298 (0.297,0.299) | <0.001 | 0.291 (0.289,0.293) | <0.001 | 0.177 (0.153,0.203) | <0.001 | 0.159 (0.134,0.188) | <0.001 |
| 1987-1991 | 0.401 (0.394,0.408) | <0.001 | 0.366 (0.358,0.374) | <0.001 | 0.256 (0.252,0.259) | <0.001 | 0.253 (0.249,0.257) | <0.001 | 0.14 (0.11,0.18) | <0.001 | 0.121 (0.089,0.164) | <0.001 |

AF/AFL, Atrial fibrillation (AF)/atrial flutter (AFL); RR, Relative Risk; CI, confidence interval.

Supplementary Table 12 Changes in DALYs of AF/AFL according aging, population growth and epidemiological change from 1990 to 2021 by sexs

| **location** | **Both** | | | **Female** | | | **Male** | | |
| --- | --- | --- | --- | --- | --- | --- | --- | --- | --- |
|  | **Aging** | **population** | **Epidemidogical** | **Aging** | **population** | **Epidemidogical** | **Aging** | **population** | **Epidemidogical** |
| Global | 56.31 | 43.17 | 0.52 | 56.53 | 44.94 | -1.47 | 56.63 | 41.39 | 1.98 |
| High SDI | 64.16 | 38.76 | -2.92 | 63.3 | 43.24 | -6.53 | 69.84 | 36.93 | -6.78 |
| High-middle SDI | 70.27 | 31.55 | -1.82 | 69.63 | 33.73 | -3.37 | 70.53 | 29.3 | 0.17 |
| Middle SDI | 64.97 | 31.69 | 3.34 | 66.26 | 32.71 | 1.03 | 63.1 | 30.7 | 6.2 |
| Low-middle SDI | 39.32 | 45.68 | 15 | 42.54 | 45.28 | 12.18 | 35.38 | 45.95 | 18.67 |
| Low SDI | 6.24 | 83.62 | 10.13 | 9.54 | 81.27 | 9.19 | 2.6 | 85.88 | 11.52 |

AF/AFL, Atrial fibrillation (AF)/atrial flutter (AFL); DALYs, disability-adjusted life years.

Supplementary Table 13 Changes in DALYs of AF/AFL according aging, population growth and epidemiological change from 1990 to 2021 in 204 countries and territories

| **location_name** | **Aging** | **population** | **Epidemidogical** |
| --- | --- | --- | --- |
| Afghanistan | -3.08 | 111.21 | -8.13 |
| Albania | 121.76 | -25.41 | 3.65 |
| Algeria | 56.01 | 43.87 | 0.12 |
| American Samoa | 88.13 | 2.95 | 8.91 |
| Andorra | 74.78 | 47.94 | -22.72 |
| Angola | -0.99 | 91.88 | 9.12 |
| Antigua and Barbuda | 22.8 | 87.39 | -10.18 |
| Argentina | 71.5 | 68.32 | -39.82 |
| Armenia | 84.74 | -16.8 | 32.06 |
| Australia | 45.71 | 58.77 | -4.48 |
| Austria | 42.41 | 15.93 | 41.66 |
| Azerbaijan | 42.14 | 46.6 | 11.26 |
| Bahamas | 55.84 | 42.42 | 1.74 |
| Bahrain | 40.18 | 75.16 | -15.34 |
| Bangladesh | 55.23 | 32.28 | 12.48 |
| Barbados | 68.24 | 31.23 | 0.53 |
| Belarus | 101.73 | -31.69 | 29.96 |
| Belgium | 89.56 | 30.25 | -19.81 |
| Belize | 22.39 | 69.87 | 7.74 |
| Benin | -18.71 | 109.42 | 9.29 |
| Bermuda | 68.75 | -11.55 | 42.8 |
| Bhutan | 69.03 | 13.76 | 17.22 |
| Bolivia (Plurinational State of) | 43.35 | 55.25 | 1.4 |
| Bosnia and Herzegovina | 130.37 | -46.34 | 15.97 |
| Botswana | 43.04 | 57.95 | -0.99 |
| Brazil | 66.17 | 33.51 | 0.32 |
| Brunei Darussalam | 62.32 | 57.21 | -19.53 |
| Bulgaria | 140.1 | -62.56 | 22.46 |
| Burkina Faso | -6.58 | 87.19 | 19.38 |
| Burundi | -31.8 | 142.23 | -10.44 |
| Cabo Verde | 8.81 | 55.67 | 35.52 |
| Cambodia | 48.93 | 44.43 | 6.64 |
| Cameroon | -8.62 | 101.91 | 6.71 |
| Canada | 80.06 | 47.47 | -27.53 |
| Central African Republic | -6.78 | 107.34 | -0.56 |
| Chad | -75.19 | 152.75 | 22.44 |
| Chile | 64.45 | 32.96 | 2.58 |
| China | 84.42 | 17.26 | -1.68 |
| Colombia | 70.5 | 35.53 | -6.03 |
| Comoros | 54.33 | 50.42 | -4.75 |
| Congo | 14.83 | 88.86 | -3.69 |
| Cook Islands | 73.49 | 11.51 | 15 |
| Costa Rica | 63.61 | 39.34 | -2.95 |
| Côte d'Ivoire | 25.89 | 67.88 | 6.22 |
| Croatia | 107.47 | -21.93 | 14.45 |
| Cuba | 103.67 | 6.26 | -9.93 |
| Cyprus | 120.97 | 96.2 | -117.17 |
| Czechia | 63.49 | 4.19 | 32.32 |
| Democratic People's Republic of Korea | 71.56 | 28.64 | -0.2 |
| Democratic Republic of the Congo | 3.94 | 89.56 | 6.5 |
| Denmark | 51.43 | 23.14 | 25.43 |
| Djibouti | 31.51 | 68.25 | 0.23 |
| Dominica | 116.93 | -24.14 | 7.21 |
| Dominican Republic | 65.53 | 39.53 | -5.06 |
| Ecuador | 57.1 | 56.44 | -13.54 |
| Egypt | 23.16 | 75.75 | 1.1 |
| El Salvador | 71.73 | 21.62 | 6.65 |
| Equatorial Guinea | -29 | 120.16 | 8.84 |
| Eritrea | 33.74 | 64.09 | 2.17 |
| Estonia | 91.84 | -29.35 | 37.51 |
| Eswatini | 37.71 | 49.48 | 12.8 |
| Ethiopia | 18.79 | 83.47 | -2.26 |
| Fiji | 65.87 | 22.48 | 11.65 |
| Finland | 213.49 | 36.3 | -149.79 |
| France | 109.8 | 29.56 | -39.36 |
| Gabon | -14.33 | 105.56 | 8.78 |
| Gambia | 17.87 | 67.72 | 14.41 |
| Georgia | 23.47 | 33.22 | 43.31 |
| Georgia | 23.47 | 33.22 | 43.31 |
| Georgia | 23.47 | 33.22 | 43.31 |
| Georgia | 23.47 | 33.22 | 43.31 |
| Germany | 70.03 | 9.82 | 20.15 |
| Ghana | 19.54 | 80.17 | 0.29 |
| Greece | 110.69 | -3.35 | -7.34 |

AF/AFL, Atrial fibrillation (AF)/atrial flutter (AFL); DALYs, disability-adjusted life years.

Supplementary Table 14 The predicted case number and ASR of incidence, death, and DALYs of AF/AFL to 2036 globally

| **Year** | **Incidence** | | | | | | **Death** | | | | | | **DALYs** | | | | | |
| --- | --- | --- | --- | --- | --- | --- | --- | --- | --- | --- | --- | --- | --- | --- | --- | --- | --- | --- |
|  | **Both** |  | **Famale** |  | **Male** |  | **Both** |  | **Famale** |  | **Male** |  | **Both** |  | **Famale** |  | **Male** |  |
|  | **number** | **ASR** | **number** | **ASR** | **number** | **ASR** | **number** | **ASR** | **number** | **ASR** | **number** | **ASR** | **number** | **ASR** | **number** | **ASR** | **number** | **ASR** |
| 2022 | 4648497.7 | 52.36 | 2275482 | 47.68 | 2373016 | 57.23 | 355524 | 4.45 | 211785 | 4.39 | 143739 | 4.52 | 8665608 | 102 | 4466412 | 92.97 | 4199196 | 112.42 |
| 2023 | 4798677.7 | 52.59 | 2356984 | 48.03 | 2441694 | 57.34 | 364201 | 4.42 | 216401 | 4.35 | 147800 | 4.49 | 8898298 | 101.65 | 4585331 | 92.69 | 4312967 | 112 |
| 2024 | 4956171.9 | 52.83 | 2443203 | 48.39 | 2512969 | 57.44 | 374976 | 4.38 | 222161 | 4.32 | 152815 | 4.46 | 9158813 | 101.32 | 4719560 | 92.43 | 4439253 | 111.59 |
| 2025 | 5118916.6 | 53.06 | 2533236 | 48.75 | 2585681 | 57.53 | 386343 | 4.35 | 228261 | 4.28 | 158082 | 4.43 | 9432003 | 101 | 4861638 | 92.19 | 4570365 | 111.17 |
| 2026 | 5285700.9 | 53.31 | 2626240 | 49.13 | 2659461 | 57.63 | 397402 | 4.31 | 234224 | 4.24 | 163177 | 4.39 | 9707917 | 100.69 | 5006156 | 91.97 | 4701761 | 110.76 |
| 2027 | 5454402.1 | 53.56 | 2721146 | 49.53 | 2733256 | 57.74 | 407663 | 4.28 | 239775 | 4.21 | 167888 | 4.36 | 9979115 | 100.4 | 5149134 | 91.77 | 4829981 | 110.38 |
| 2028 | 5627543 | 53.82 | 2819358 | 49.93 | 2808185 | 57.84 | 418755 | 4.25 | 245770 | 4.17 | 172985 | 4.33 | 10263733 | 100.13 | 5299963 | 91.58 | 4963770 | 110.03 |
| 2029 | 5807266.4 | 54.08 | 2922217 | 50.34 | 2885049 | 57.93 | 431739 | 4.21 | 252806 | 4.14 | 178933 | 4.3 | 10574185 | 99.88 | 5465617 | 91.41 | 5108568 | 109.67 |
| 2030 | 5991409.4 | 54.34 | 3028737 | 50.76 | 2962672 | 58.01 | 445681 | 4.18 | 260419 | 4.1 | 185263 | 4.28 | 10899124 | 99.63 | 5640772 | 91.27 | 5258352 | 109.31 |
| 2031 | 6178957.4 | 54.61 | 3138119 | 51.2 | 3040838 | 58.1 | 459888 | 4.15 | 268259 | 4.07 | 191629 | 4.25 | 11230299 | 99.41 | 5820853 | 91.15 | 5409446 | 108.97 |
| 2032 | 6367670.7 | 54.9 | 3249143 | 51.65 | 3118528 | 58.19 | 473809 | 4.12 | 276026 | 4.04 | 197784 | 4.22 | 11559503 | 99.21 | 6001427 | 91.06 | 5558076 | 108.65 |
| 2033 | 6559129.5 | 55.19 | 3362630 | 52.11 | 3196499 | 58.29 | 488625 | 4.09 | 284327 | 4.01 | 204298 | 4.19 | 11900367 | 99.03 | 6189475 | 90.98 | 5710892 | 108.35 |
| 2034 | 6755503.4 | 55.48 | 3479890 | 52.59 | 3275613 | 58.37 | 505254 | 4.06 | 293670 | 3.98 | 211585 | 4.17 | 12264110 | 98.85 | 6391230 | 90.91 | 5872880 | 108.04 |
| 2035 | 6955252.1 | 55.77 | 3600229 | 53.07 | 3355023 | 58.44 | 522984 | 4.04 | 303708 | 3.95 | 219277 | 4.14 | 12641249 | 98.69 | 6602155 | 90.88 | 6039094 | 107.73 |
| 2036 | 7157841.9 | 56.07 | 3723153 | 53.57 | 3434689 | 58.53 | 541137 | 4.01 | 314089 | 3.93 | 227048 | 4.12 | 13024321 | 98.56 | 6817798 | 90.87 | 6206523 | 107.45 |

ASR, age-standardized rate; AF/AFL, Atrial fibrillation (AF)/atrial flutter (AFL); DALYs, disability-adjusted life years.


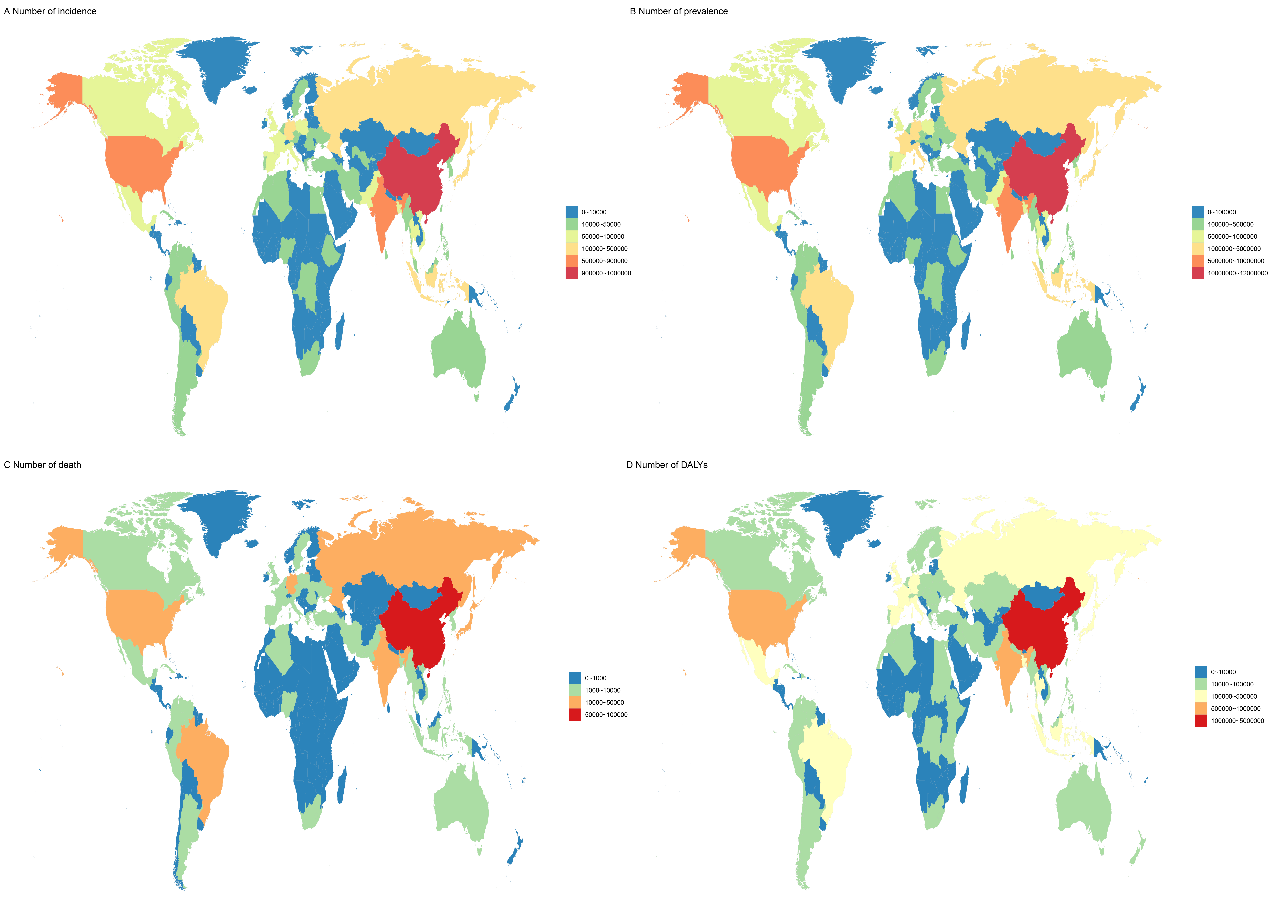


**Supplementary Fig. 1** Geographic heat map. (A) Number of AF/AFL patient incidence from 1990 to 2021. (B) Number of AF/AFL patient prevalence from 1990 to 2021. (C) Number of AF/AFL patient death from 1990 to 2021. (D) Number of AF/AFL patient DALYs from 1990 to 2021. AF/AFL, Atrial fibrillation (AF)/atrial flutter (AFL); DALYs, disability-adjusted life years.


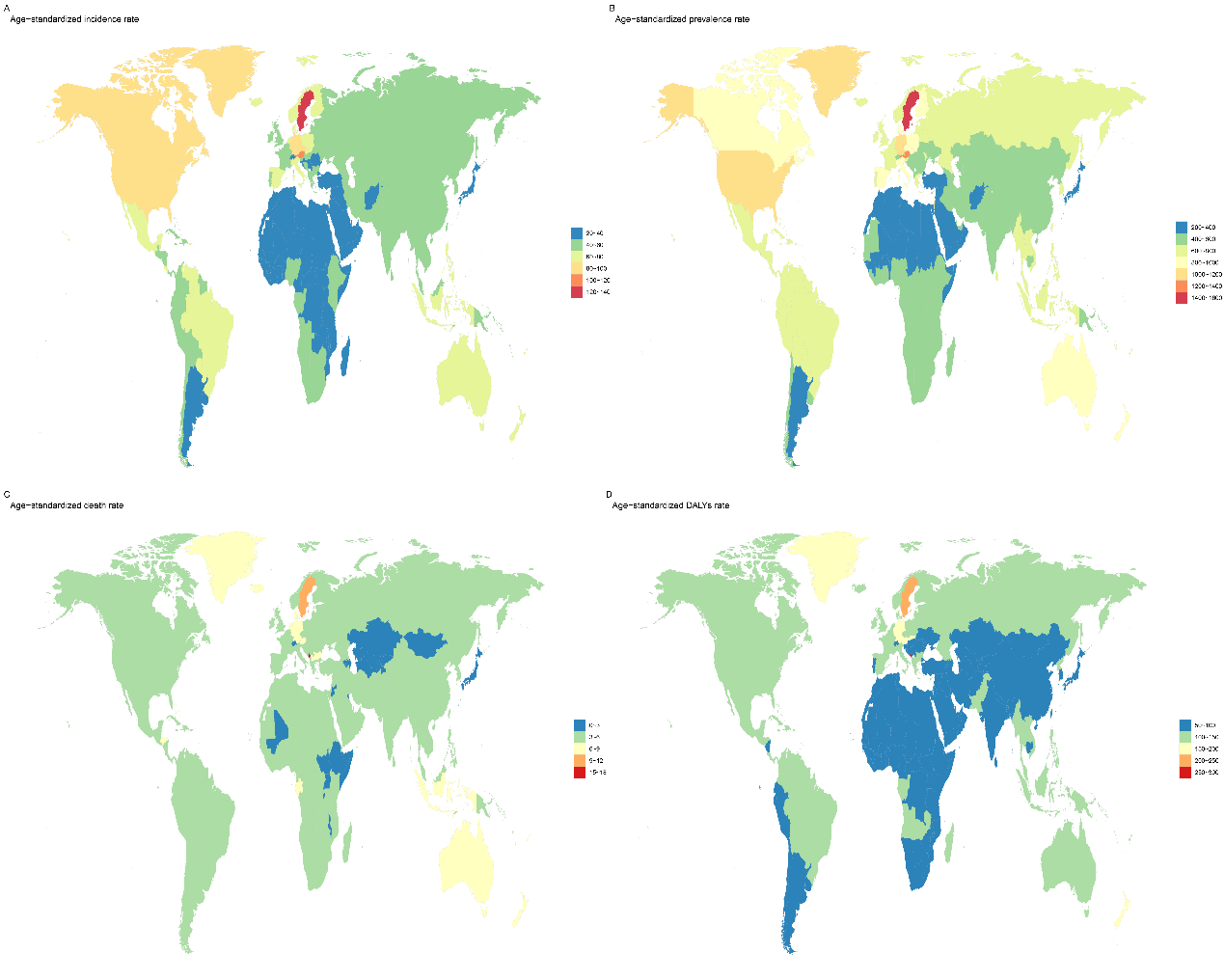


**Supplementary Fig. 2** Geographic heat map. (A) ASR of AF/AFL patient incidence from 1990 to 2021. (B) ASR of AF/AFL patient prevalence from 1990 to 2021. (C) ASR of AF/AFL patient death from 1990 to 2021. (D) ASR of AF/AFL patient DALYs from 1990 to 2021. ASR, age-standardized rate; AF/AFL, Atrial fibrillation (AF)/atrial flutter (AFL); DALYs, disability-adjusted life years.


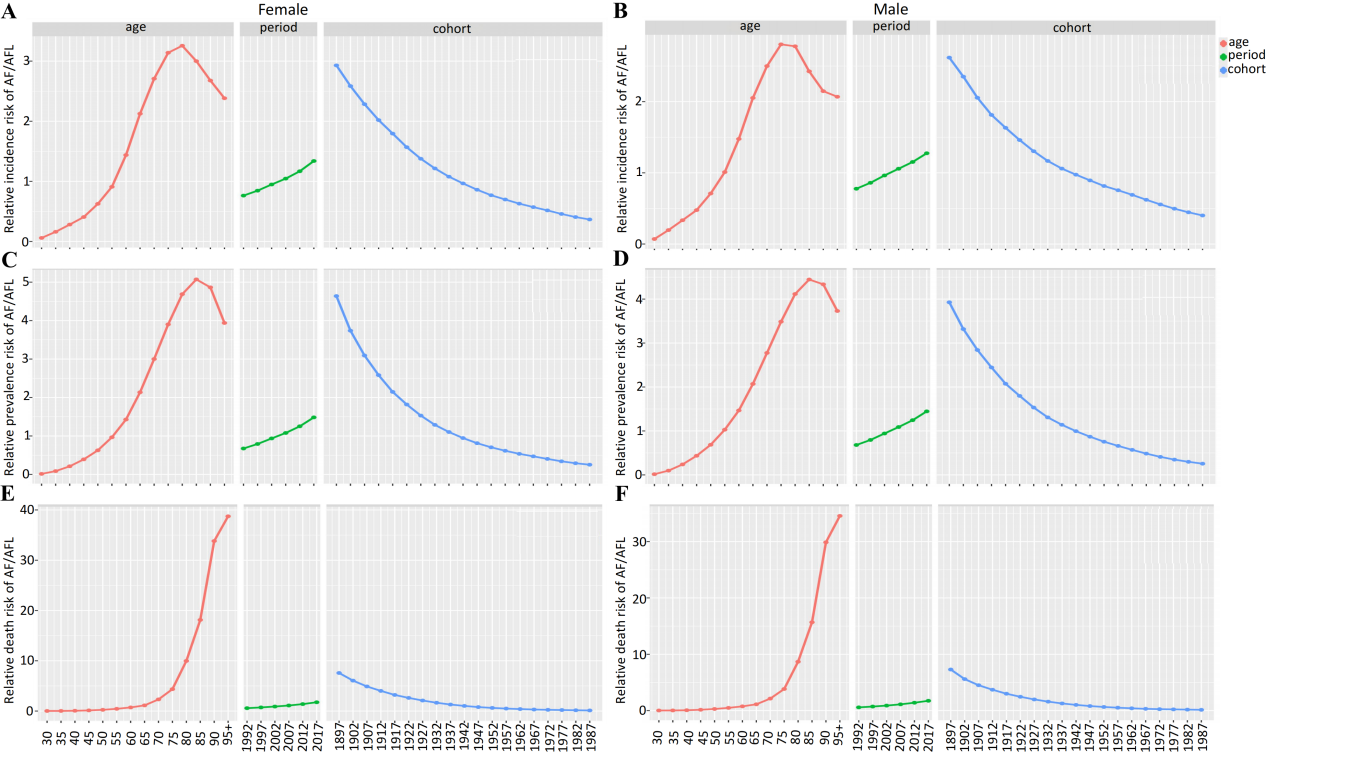


**Supplementary Fig. 3** The effects of age, period, and birth cohort on the relative risk of AF/AFL incidence, prevalence, and death stratified by sexes.(A) Incidence; (B) Prevalence; (C) Death. AF/AFL, Atrial fibrillation (AF)/atrial flutter (AFL).


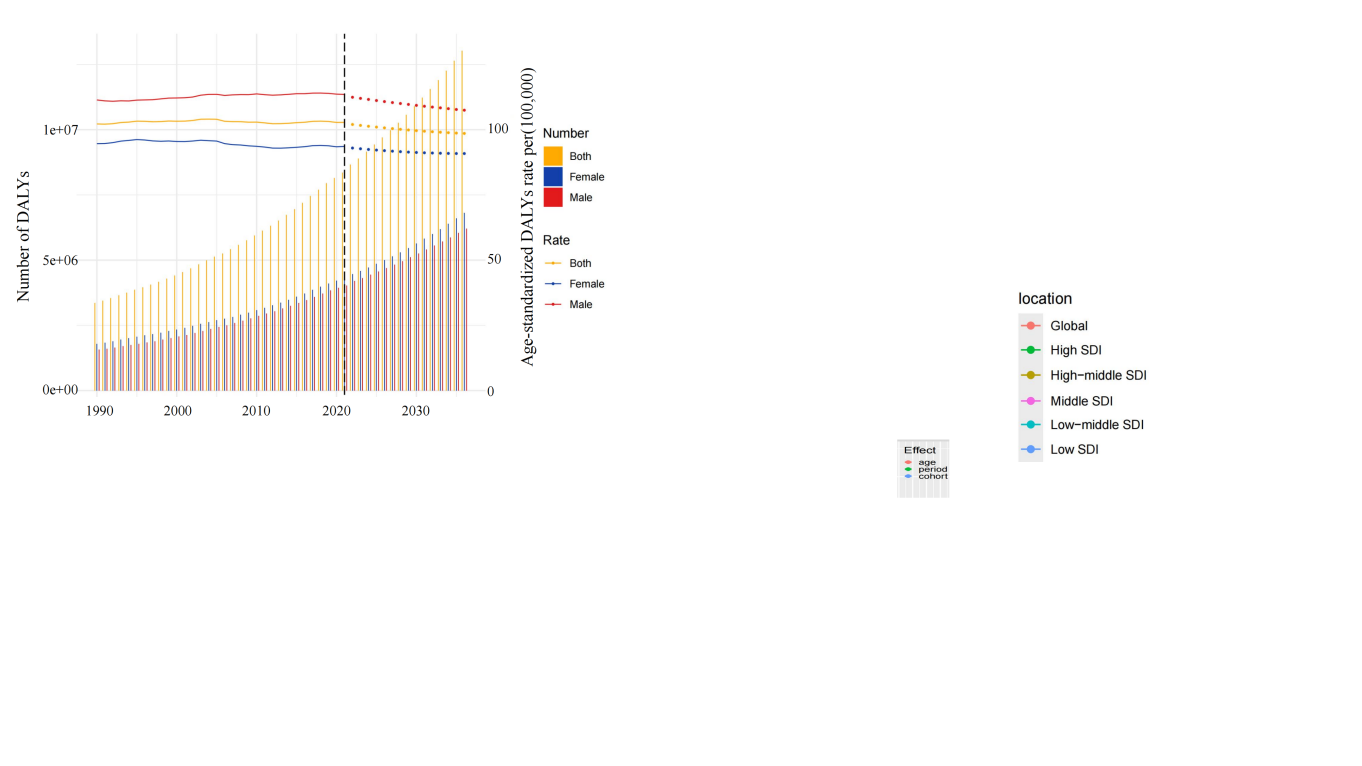


**Supplementary Fig. 4** The predicted case number and ASR of DALYs of AF/AFL to 2036 Projects the ASRs and numbers of AF/AFL DALYs by gender from 1990 to 2036 based on the BAPC model. ASRs, age-standardized rates; AF/AFL, Atrial fibrillation (AF)/atrial flutter (AFL); BAPC, Bayesian age–period–cohort.
