# Supplementary figures and images for: Global Epidemiological Transition of Atrial Fibrillation/Flutter (1990–2021): Multidimensional Burden Dynamics and Socioeconomic Health Gradients Across 204 Countries and Territories
Source: Rev Cardiovasc Med. 2025 Dec 18;26(12):45091. doi: 10.31083/RCM45091 (PMC12781011; doi:10.31083/RCM45091)

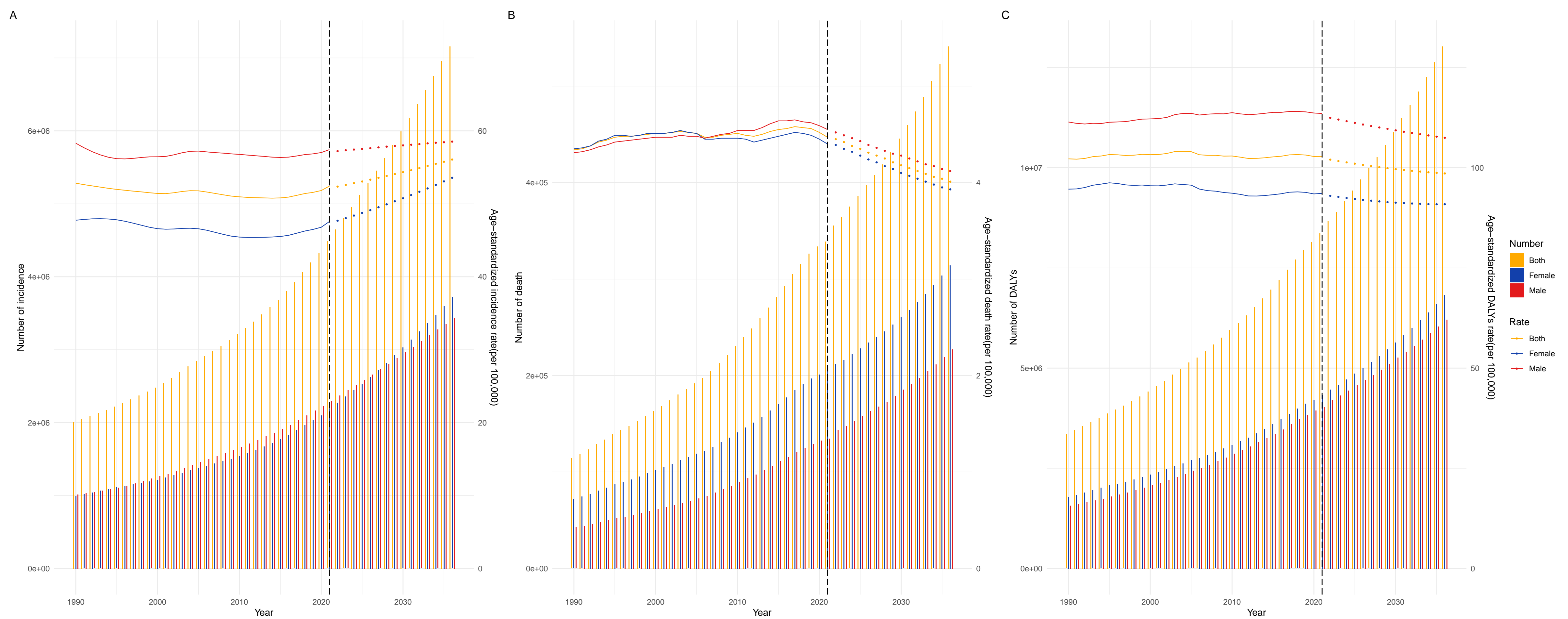

Supplement: Supplementary file 1 [file 2153-8174-26-12-45091-s1.zip › Fig. 9 and Supplementary Fig. 4.pdf]

A Number of incidence

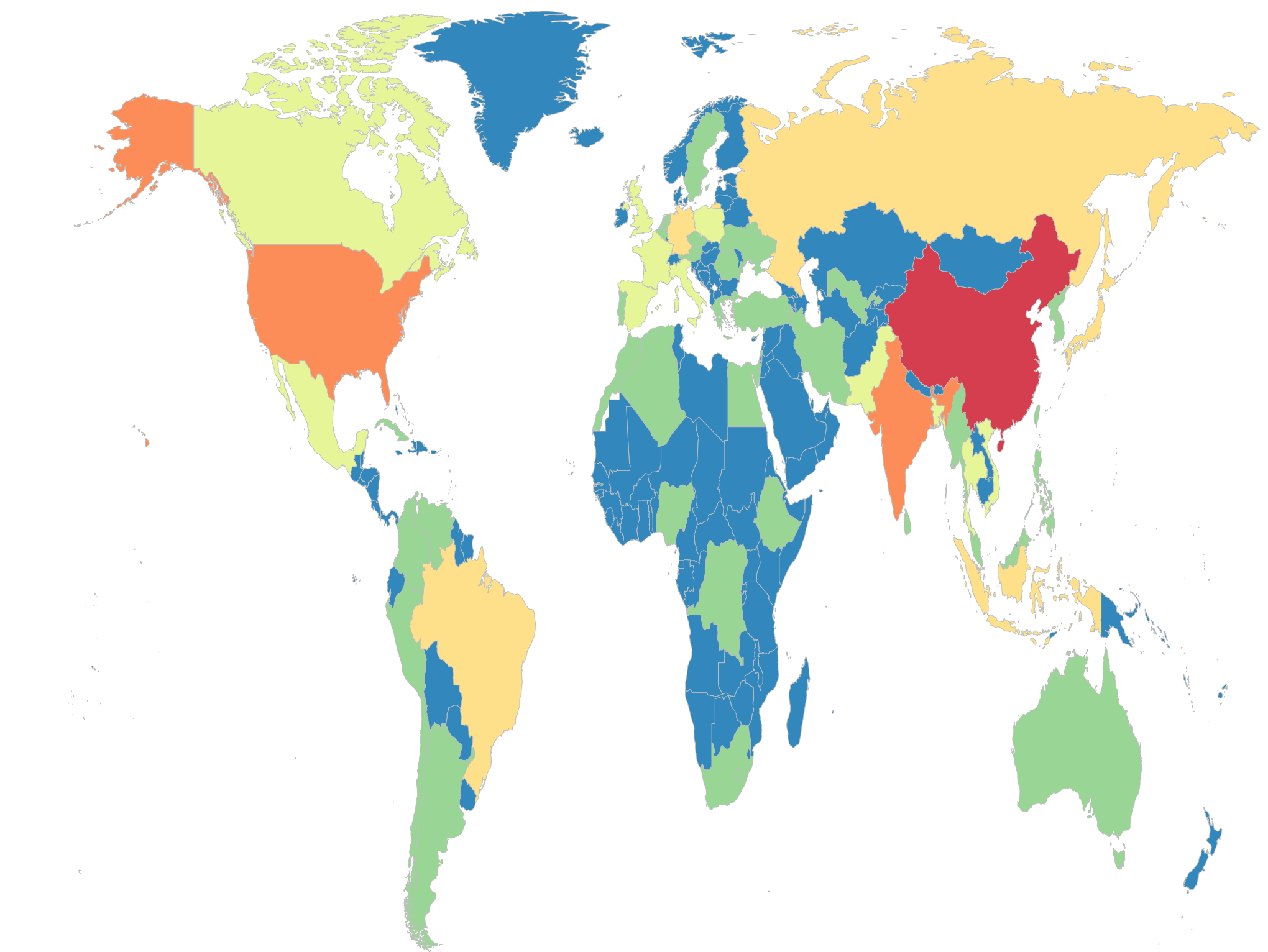

B Number of prevalence

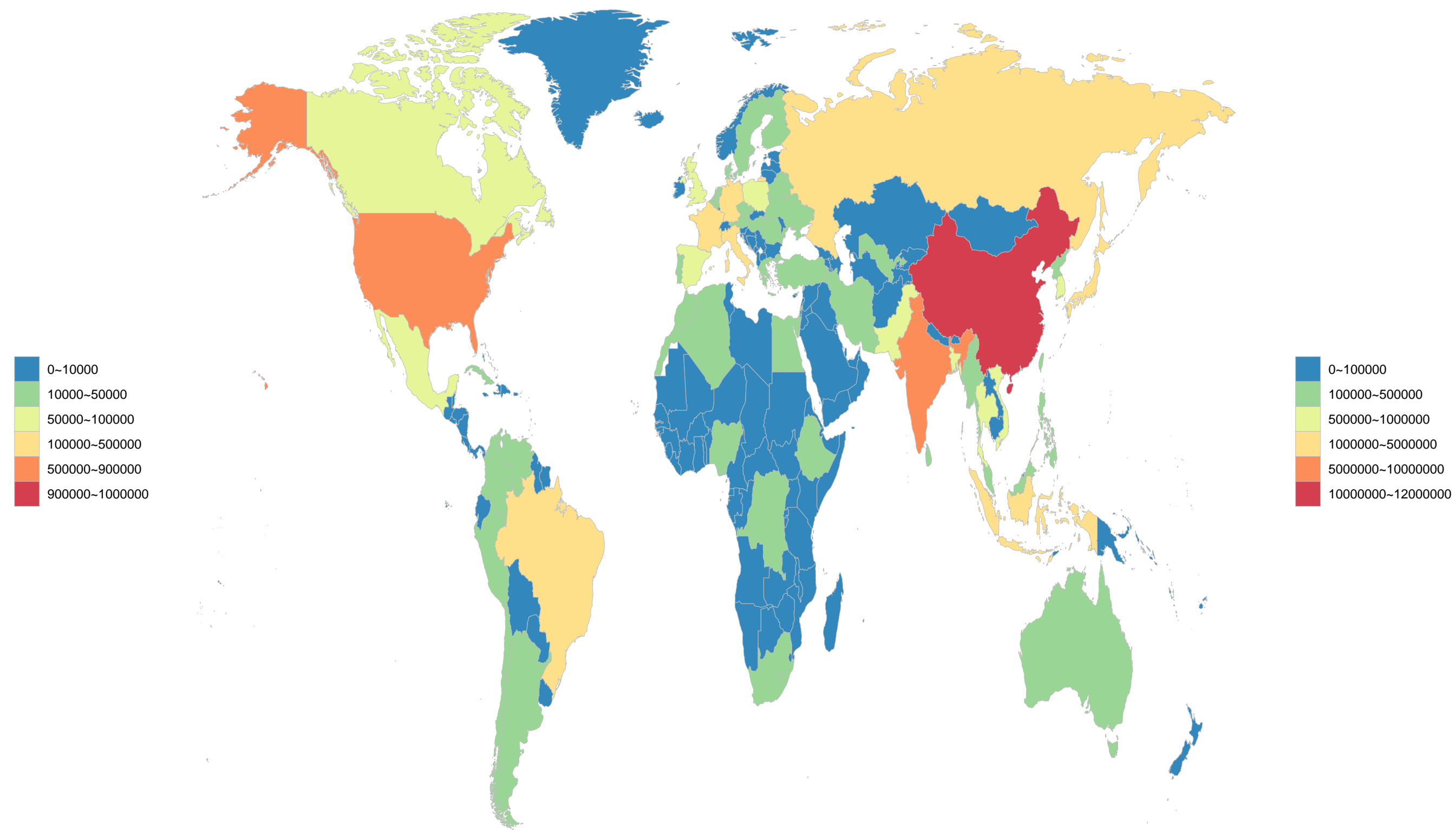

C Number of death

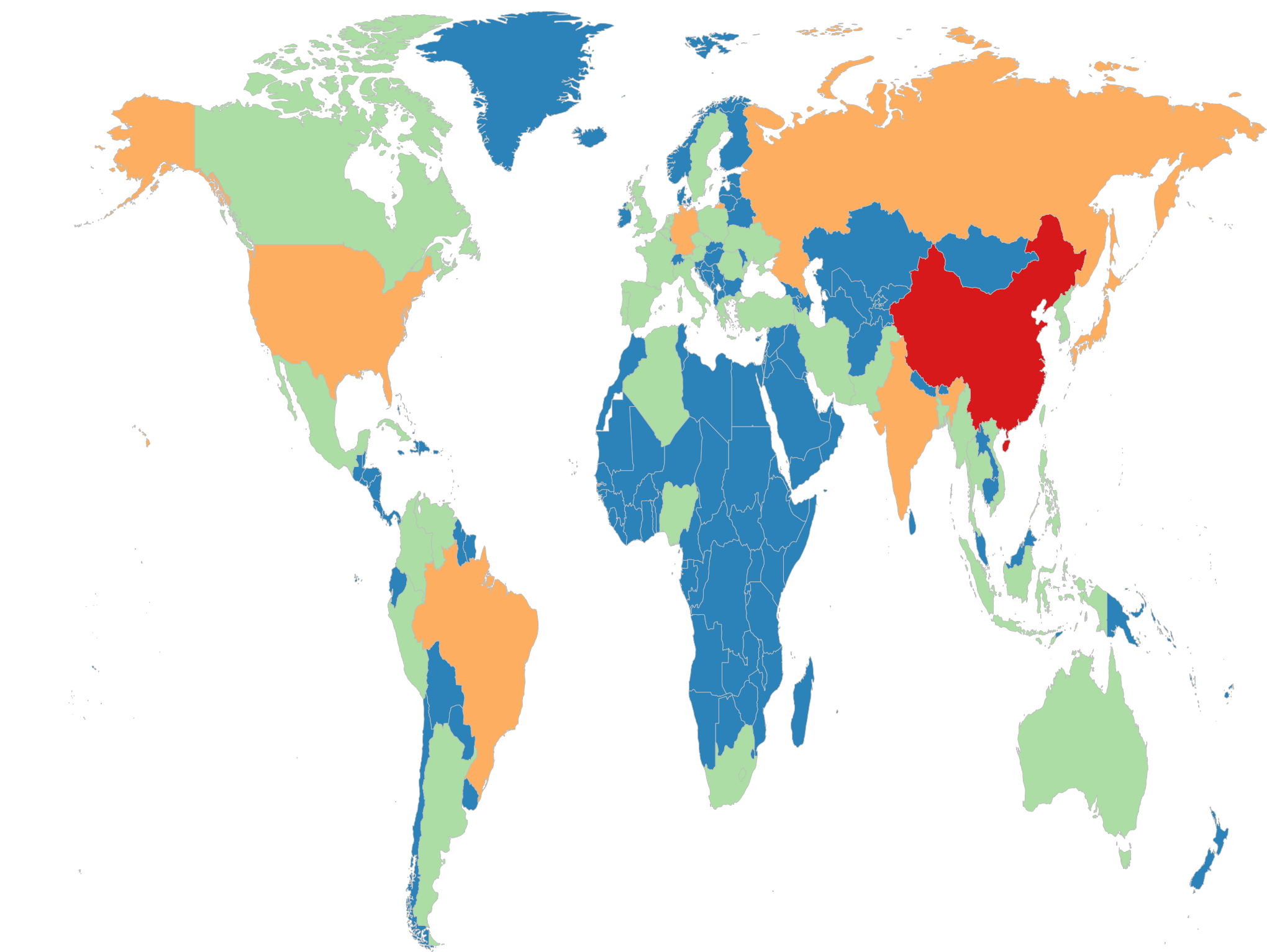

D Number of DALYs

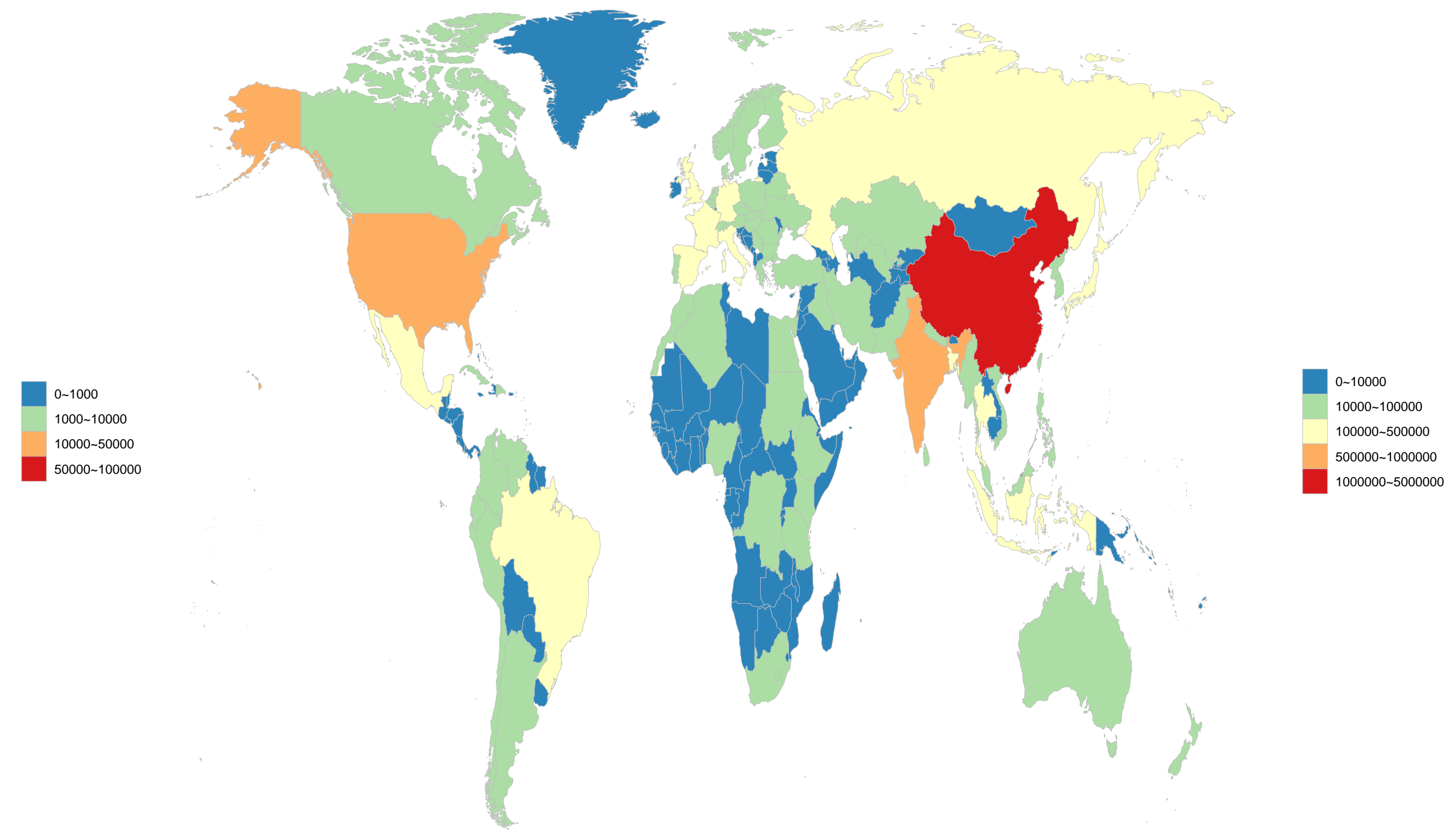

Supplement: Supplementary file 1 [file 2153-8174-26-12-45091-s1.zip › Supplementary Fig. 1.pdf]

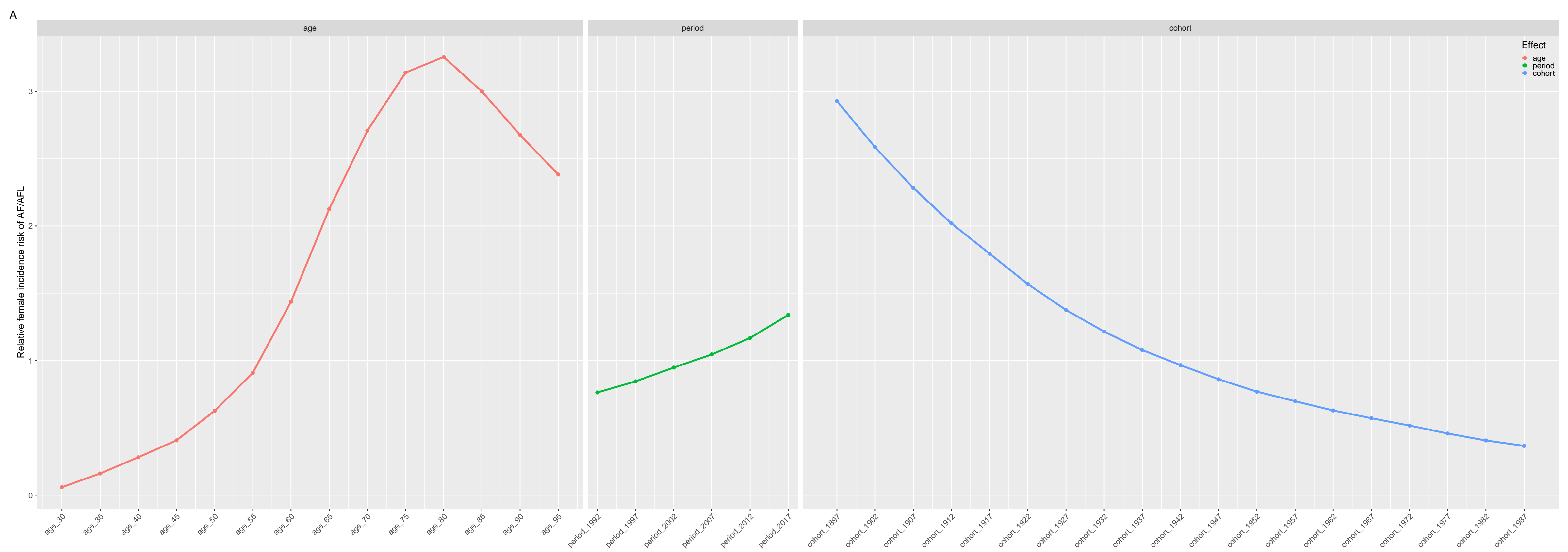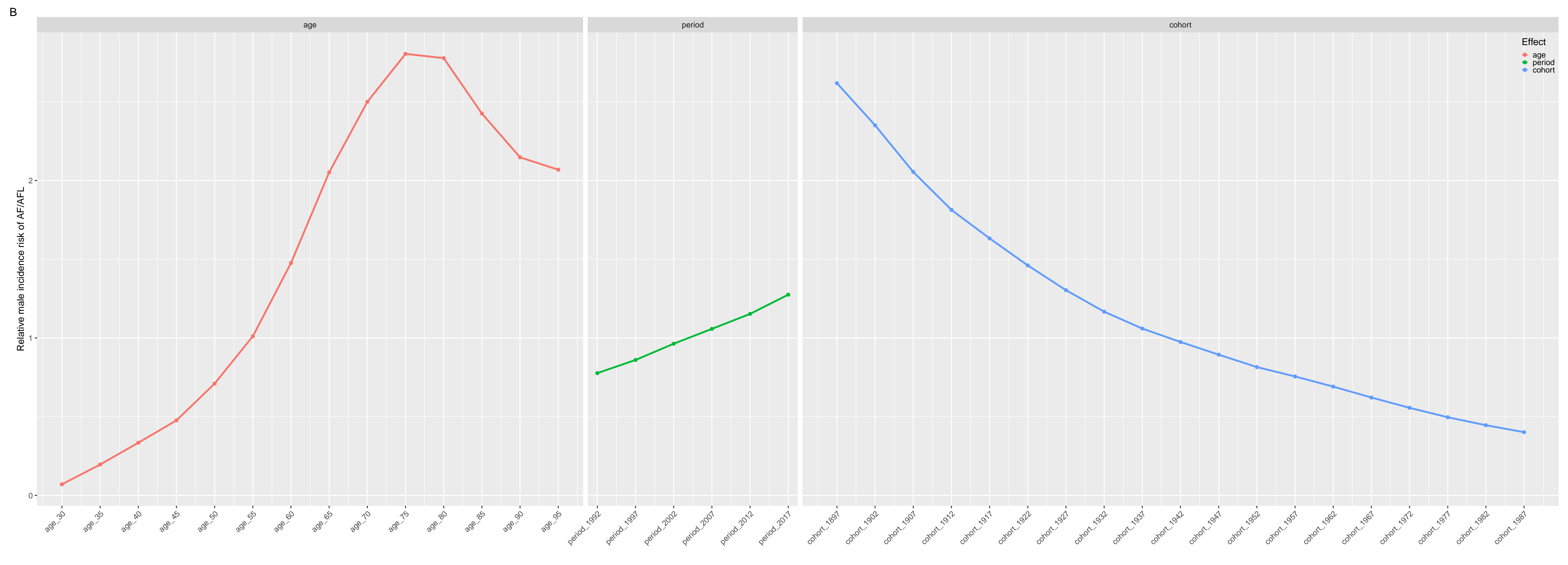

Supplement: Supplementary file 1 [file 2153-8174-26-12-45091-s1.zip › Supplementary Fig. 3/FigS3A.pdf]

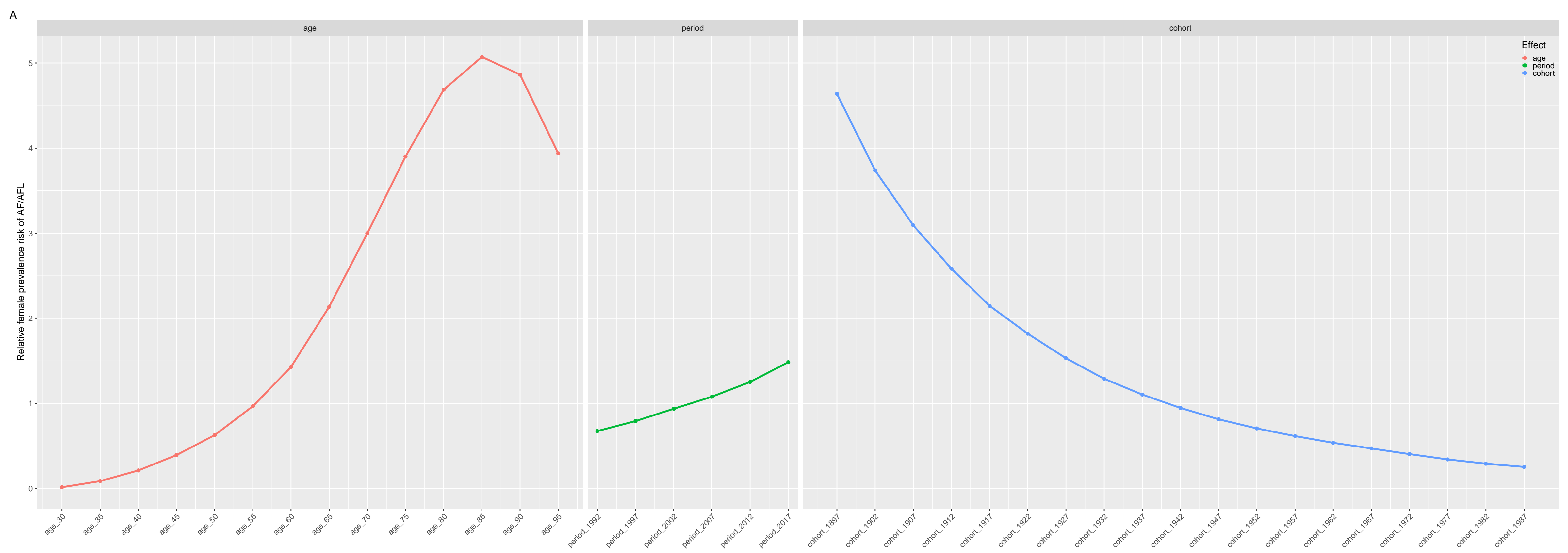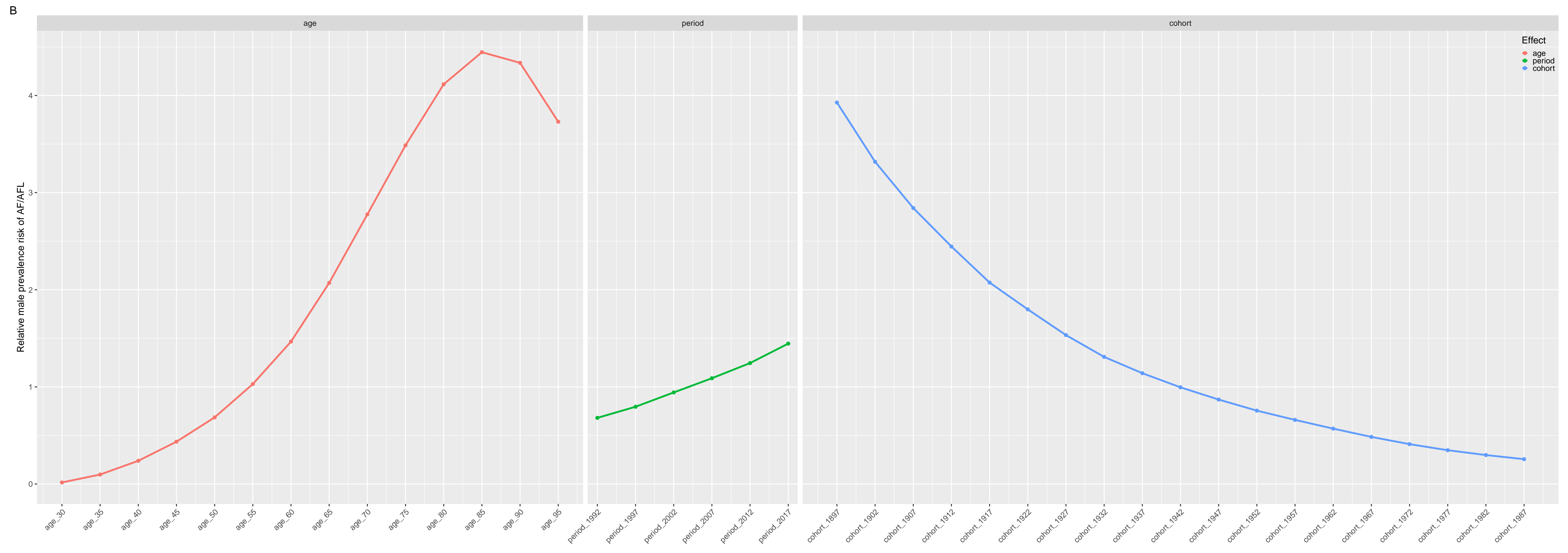

Supplement: Supplementary file 1 [file 2153-8174-26-12-45091-s1.zip › Supplementary Fig. 3/FigS3B.pdf]
