# Supplementary material for: Global Epidemiological Transition of Atrial Fibrillation/Flutter (1990–2021): Multidimensional Burden Dynamics and Socioeconomic Health Gradients Across 204 Countries and Territories
Source: Rev Cardiovasc Med. 2025 Dec 18;26(12):45091. doi: 10.31083/RCM45091 (PMC12781011; doi:10.31083/RCM45091)
Supplement: Supplementary file 1 [file FigS3C.pdf › Supplementary Fig. 3/FigS3C.pdf]

A

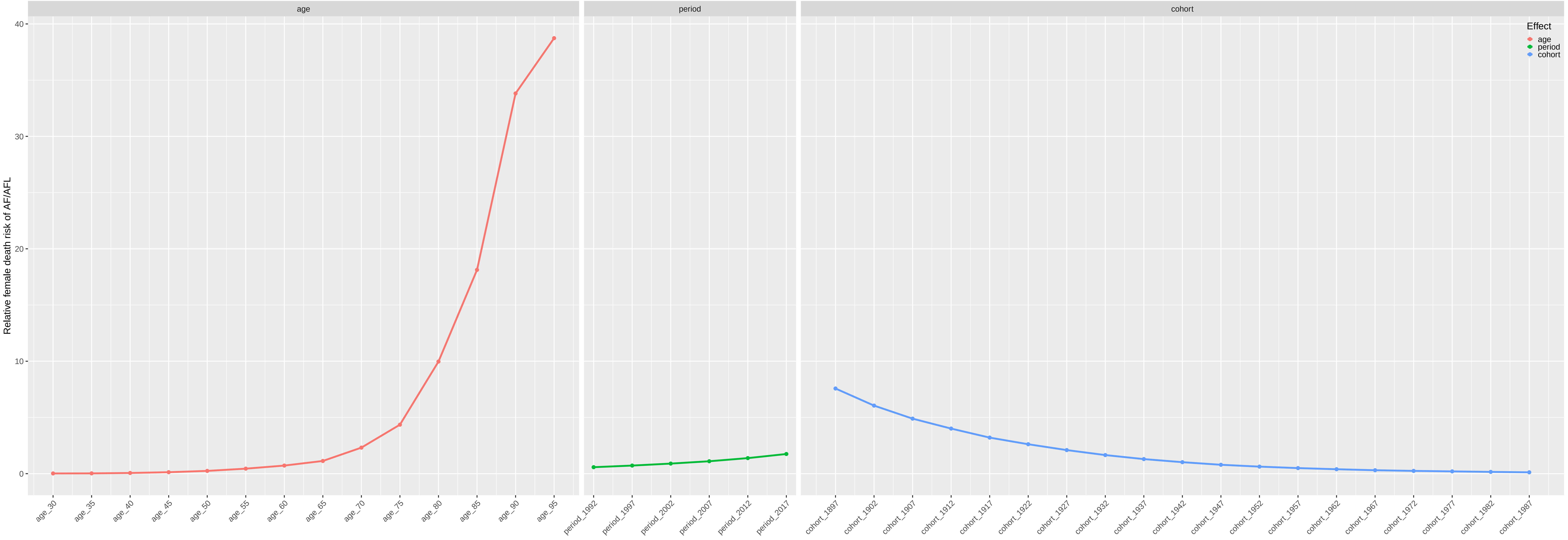
age
period
cohort
Relative female death risk of AF/AFL
0
10
20
30
40
Effect
age
period
cohort
age_30 age_35 age_40 age_45 age_50 age_55 age_60 age_65 age_70 age_75 age_80 age_85 age_90 age_95
period_1992 period_1997 period_2002 period_2007 period_2012 period_2017
cohort_1897 cohort_1902 cohort_1907 cohort_1912 cohort_1917 cohort_1922 cohort_1927 cohort_1932 cohort_1937 cohort_1942 cohort_1947 cohort_1952 cohort_1957 cohort_1962 cohort_1967 cohort_1972 cohort_1977 cohort_1982 cohort_1987

B

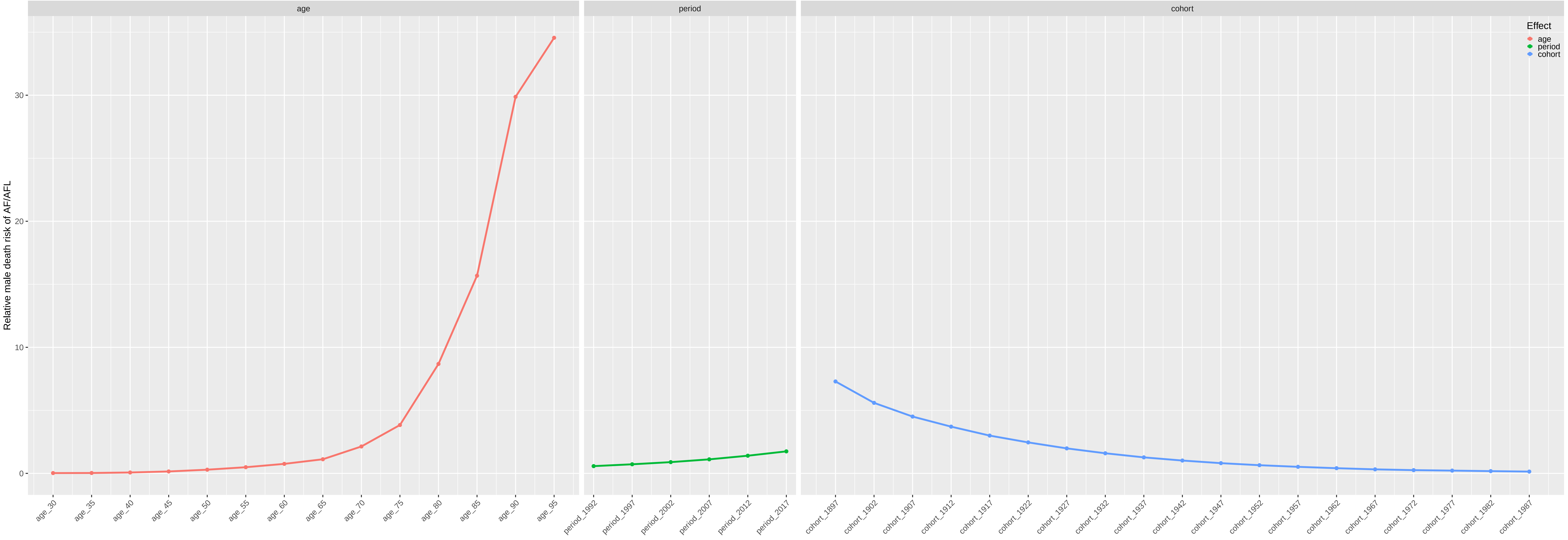
age
period
cohort
Relative male death risk of AF/AFL
0
10
20
30
Effect
age
period
cohort
age_30 age_35 age_40 age_45 age_50 age_55 age_60 age_65 age_70 age_75 age_80 age_85 age_90 age_95
period_1992 period_1997 period_2002 period_2007 period_2012 period_2017
cohort_1897 cohort_1902 cohort_1907 cohort_1912 cohort_1917 cohort_1922 cohort_1927 cohort_1932 cohort_1937 cohort_1942 cohort_1947 cohort_1952 cohort_1957 cohort_1962 cohort_1967 cohort_1972 cohort_1977 cohort_1982 cohort_1987
